# Long-term outcomes of psychological interventions on children and young people’s mental health: A systematic review and meta-analysis

**DOI:** 10.1371/journal.pone.0236525

**Published:** 2020-11-16

**Authors:** Stephen Pilling, Peter Fonagy, Elizabeth Allison, Phoebe Barnett, Chloe Campbell, Matthew Constantinou, Tessa Gardner, Nicolas Lorenzini, Hannah Matthews, Alana Ryan, Sofia Sacchetti, Alexandra Truscott, Tamara Ventura, Kate Watchorn, Craig Whittington, Tim Kendall

**Affiliations:** 1 Research Department of Clinical, Educational and Health Psychology, University College London, London, United Kingdom; 2 Royal College of Psychiatrists National Collaborating Centre for Mental Health, London, United Kingdom; 3 RWE Literature Synthesis and Biostatistics, Sanofi, Swiftwater, Pennsylvania, United States of America; Medical University of Vienna, AUSTRIA

## Abstract

**Background:**

Over 600 RCTs have demonstrated the effectiveness of psychosocial interventions for children and young people’s mental health, but little is known about the long-term outcomes. This systematic review sought to establish whether the effects of selective and indicated interventions were sustained at 12 months.

**Method:**

We conducted a systematic review and meta-analysis focusing on studies reporting medium term outcomes (12 months after end of intervention).

**Findings:**

We identified 138 trials with 12-month follow-up data, yielding 165 comparisons, 99 of which also reported outcomes at end of intervention, yielding 117 comparisons. We found evidence of effect relative to control at end of intervention (K = 115, g = 0.39; 95% CI: 0.30–0.47 I^2^ = 84.19%, N = 13,982) which was maintained at 12 months (K = 165, g = 0.31, CI: 0.25–0.37, I^2^ = 77.35%, N = 25,652) across a range of diagnostic groups. We explored the impact of potential moderators on outcome, including modality, format and intensity of intervention, selective or indicated intervention, site of delivery, professional/para-professional and fidelity of delivery. We assessed both risk of study bias and publication bias.

**Conclusions:**

Psychosocial interventions provided in a range of settings by professionals and paraprofessionals can deliver lasting benefits. High levels of heterogeneity, moderate to high risk of bias for most studies and evidence of publication bias require caution in interpreting the results. Lack of studies in diagnostic groups such as ADHD and self-harm limit the conclusions that can be drawn. Programmes that increase such interventions’ availability are justified by the benefits to children and young people and the decreased likelihood of disorder in adulthood.

## Introduction

The under-treatment of children and young people’s mental disorder is ubiquitous globally [1], yet problems at this age are harbingers of adult disorders. Fifty percent of all adult mental ill-health is diagnosable by 14 years of age, and 75% by 18–25 years [2, 3]. Many children and young people also experience significant sub-threshold symptoms which may be precursors to the development of a mental disorder [4–6]. Access to treatments associated with long-term benefits could both address the unmet need for children and young people and reduce adult rates of mental ill-health.

Universal prevention efforts to address children and young people’s mental health have not yet reached consensus on how to reduce the burden associated with mental health problems [7–9]. Despite considerable efforts, the evidence for universal programs is not robust and there is uncertainty about their long-term impact [10]. The challenge of universal prevention is addressing the wide range of interrelated risk factors (individual, family, school, community) which require comprehensive multilevel approaches [10].

In general, selective and indicated prevention programmes appear more clinically and cost-effective [11]. Given the complications of pharmacological interventions there is a natural preference for psychosocial treatments for children and young people [12]. Psychosocial interventions for mental disorders in children and young people are known to be efficacious [13, 14]. A recent comprehensive meta-analysis reported medium end-of-treatment effect sizes based on 447 studies (13). However, there are no existing systematic reviews which report long-term treatment outcomes across a broad range of disorders, which is of particular importance given that the majority of mental disorders are identifiable before the age of 18 years. Understanding whether the benefits of treatment are sustained can inform policy priorities for children and young people’s mental health services and this review was undertaken in response to a request from United Kingdom’s English Department of Health to examine the overall long-term effects of psychological interventions. Further, while these reviews have focused on treatment modality as a predictor of outcome, other important parameters have not been explored, including the level of training of those offering interventions, the setting in which interventions are provided and the dose required to achieve long-term outcomes.

## Materials and methods

### Protocol and registration

This systematic review and meta-analysis adhered to the Preferred Reporting Items for Systematic reviews and Meta-Analysis (PRISMA) guidelines. A protocol was developed and registered on PROSPERO (CRD42017081290). The protocol was adhered to except for the following deviations: (1) we undertook additional, exploratory subgroup analyses to explore heterogeneity in the data, and (2) we placed a stronger emphasis on long term outcomes, as end-of-treatment data has been comprehensively summarised in a recent report. All end of treatment data is presented as per protocol.

### Objective

This review was undertaken to guide a major UK policy initiative [15], in order to explore (1) whether the effects of selective and indicated interventions were sustained in the longer term, (2) what models of intervention for which disorders had the most promising long-term outcomes, (3) what level of training and support was required for effective provision of interventions (4), whether delivery site (school, community or health setting) moderates the impact of interventions, and (5) what conditions are required to ensure robust provision of evidence-based interventions.

### Eligibility criteria

All randomised controlled trials of psychological interventions for children or young people between 4 and 18 years old with or at risk of developing a mental health disorder, were potentially eligible for inclusion. Eligible mental health disorders comprised: anxiety disorders (including generalised anxiety disorder, obsessive compulsive disorder, panic disorder, social anxiety disorder and phobic disorders); conduct disorders (including oppositional defiant disorder and conduct disorder); depressive disorders (including depression and clinically significant sub-threshold symptoms); eating disorders (including anorexia nervosa, bulimia nervosa and binge eating disorder); post-traumatic stress disorder; substance misuse (including drug and alcohol misuse); self-harm; and attention deficit hyperactivity disorder. Studies eligible for inclusion were those where the mean age of the sample was between 4 and 18, interventions were compared against a no-treatment control, wait list, attentional control, treatment as usual or an active intervention control and reported outcomes at 9–18 months post-treatment. We chose this timeframe because (a) very few studies collect data beyond 18 months, (b) intercurrent treatments present a major challenge for interpreting outcomes beyond this and (c) the majority of relapse occurs within the first year following treatment completion [16, 17]. Studies were excluded if their participant sample were recruited from inpatient settings (as the severity of the disorders in in-patient populations were unlikely to initially treated in school or community settings), had only a solely pharmacological control arm (as we wanted these intervention to be deliverable in school settings where pharmacological interventions were not routinely available), evaluated universal preventive interventions (as evidence suggested they may not have lasting effects), were published only as dissertations, abstracts or conference proceedings or were from non-OECD countries (as we wanted to considered a range of contextual factors such which could only be explored in OECD countries).

### Information sources

The following data bases were searched: PsycINFO; EMBASE; MEDLINE; ERIC (Educational Resources Index); BEI (British Education Index); the Cochrane Library (all databases); Specialised Register of the Cochrane Common Mental Disorders Group (CCMD-CTR); Headspace Research Database (National Youth Mental Health Foundation, Australia. Searches were restricted to 1960–2017 and English language only. The date of the last search was 21st^st^ May 2019. Reference lists of all included studies were also hand-searched to identify further relevant studies.

### Search strategy

A comprehensive search strategy was developed and all relevant bibliographic databases were searched with terms modified for each specific database. Search strategies are included in S1 Fig.

### Study selection

Each paper was identified as eligible for inclusion by at least two reviewers. Three reviewers independently screened all abstracts identified in the initial search and excluded studies that did not meet inclusion criteria. Full-text articles were subsequently reviewed in duplicate, and in cases of disagreement consensus was achieved through referral to a senior reviewer (SP or PF).

### Data collection process

Seven categories of data were extracted using a standardized data extraction form. All data items were double extracted.

### Data items

The following data items were extracted: demographic and clinical characteristics of the sample; programme type (selective or indicated; we included treatment interventions in the indicated category because inclusion criteria for these two types of interventions are often very similar, e.g. scoring above a certain value on a symptom severity scale); programme content including manualization, mode of delivery, duration and intensity of the intervention (that is the time over which the intervention was provided and the total time spent in delivering the intervention); comparator type (treatment as usual/waitlist/attentional control/no treatment control or active comparator), content, mode of delivery and duration of the comparator; intervention location (US or non-US); intervention setting (school, community or clinic setting); intervention agent (teacher, professional or paraprofessional); and studies’ methodological characteristics (see quality assessment below). Based on expert consensus a hierarchy of preferred outcomes and a method for identifying outcomes in studies reporting multiple outcomes was specified for each disorder prior to data extraction of outcome measures (see S2 File). This determined the extraction of outcomes at baseline 12-month follow up, and at post-intervention where available.

### Risk of bias

The Cochrane Risk of Bias tool was used to assess the methodological quality of the eligible studies [18]. The impact of publication bias and heterogeneity was assessed by visual assessment and statistical analysis of funnel plots [19]. We also assessed the impact of date of publication on the study outcome. All methods were considered in the interpretation of the results.

### Summary measures

We calculated overall summary estimates and 95% CIs with a random-effects meta-analysis, which is to be preferred when there are high levels of heterogeneity [20], using Comprehensive Meta-Analysis software (CMA V3). Hedges’ g was used as a summary statistic to facilitate comparisons within and between disorders. The majority of trials reported continuous outcomes (123/138 at follow-up, 99/99 at end of intervention); where this was not the case dichotomous outcomes (odds ratios) were converted to Hedges’ g values.

### Data analysis

We conducted subgroup analyses by performing a series of separate meta-analyses to explore the associations between each of a range of moderators alone and by disorder, (see Table 2A for a complete list of all moderators) and ESs at post-intervention and 12-month follow-up. Subgroup analyses were conducted using a random-effects ANOVA, which partitions the variance (*Q*) into within-study (*Q*_*W*_) and between-study (*Q*_*B*_) components using random-effects weights, and is equivalent to the meta-regression approach with binary indictors (Ref: https://www.meta-analysis.com/downloads/Meta-analysis%20Subgroups%20analysis.pdf). We did not assume a common within-study variance across levels of the moderator/subgroups because of the likelihood of substantial heterogeneity. We used the *Q*_*B*_ variance component (equivalent to *Q*_*M*_ omnibus test in meta-regression) to determine whether the effect size was differentially associated with different levels of a moderator and compared the direction of significant between levels using confidence intervals.

We reported change scores (K = 111 from 99 studies at end of intervention and K = 165 from 138 studies at follow up) and adjusted for baseline scores inserting a correlation of 0.75. We considered CIs that did not overlap the line of no effect to be statistically significant and a Hedges’ g of 0.2 or greater to be of clinical importance [21]. The heterogeneity between studies was calculated using the heterogeneity I^2^ statistic where an estimate above 40% suggests presence of heterogeneity [22].

All analyses were done using CMA V3. We chose to use Egger’s test of bias rather than Orwin’s failsafe N because Orwin’s test is not available for a random effects meta-analysis in CMA V3.

## Results

### Study selection

**Fig 1:**
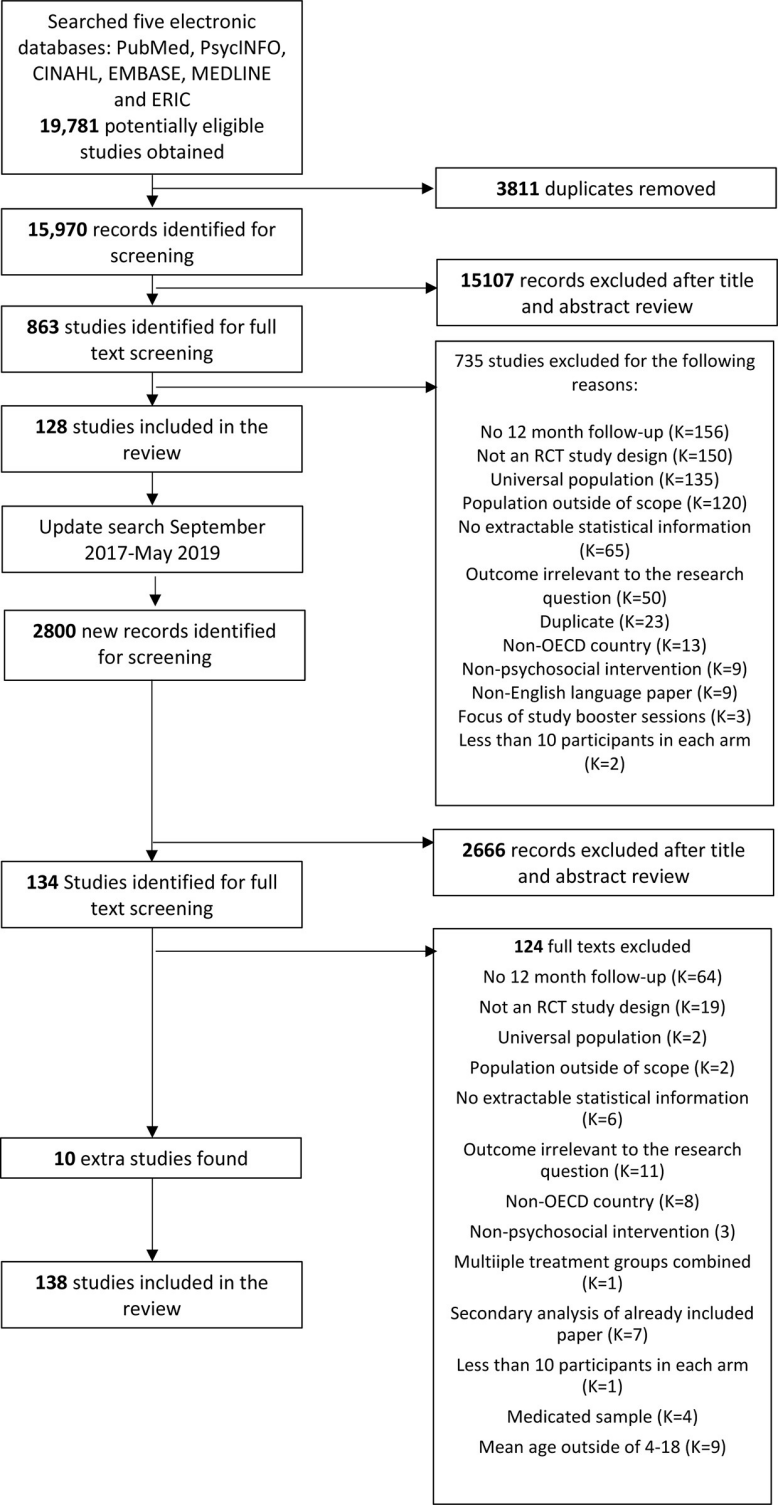
PRISMA diagram.

A total of 19,781 reports were identified in the initial search from which 3,811 were removed as duplicates. 15,970 titles and abstracts were then reviewed, identifying 863 potential studies for inclusion. The reviewers independently screened the full text of these and excluded 735 that did not meet inclusion or met exclusion criteria. This resulted in 128 treatment trials of psychosocial interventions where 12-month follow-up data were available. This search was supplemented with an update search conducted 21/05/19, which retrieved an additional 2800 records, of which 134 studies were screened, with ten additional studies meeting inclusion criteria, resulting in a total of 138 studies included in the review. The systematic review process is depicted in Fig 1.

### Study characteristics

Summary study characteristics are presented in Table 1. At baseline the studies included a total of 14,954 participants. Sample sizes varied widely (min 20, max 1,730). The 138 included studies yielded 165 comparisons containing 12-month follow-up data which were the focus of this analysis.

**Table 1:** Study characteristics.

**Study**	**Primary disorder(s)**	**Country**	**Setting**	**Age (M (range))**	**Diagnostic status**	**Control**	**Format (individual, group, mixed)**
Arnarson 2011/2009^a^	Depression	Iceland	School	NR (14–15)	Indicated	TAU	Group
Augimeri 2007^a^	Conduct Disorder	Canada	Community	8.9 (NR-12)	Indicated	Attentional	Mixed
August. 2004	Conduct Disorder	USA	Community	6.3 (5–7)	Indicated	Waitlist	Mixed
Barrett 1996	Anxiety	Australia	Clinic	9.3 (7–14)	Indicated	Active	Individual
Barrett 1998	Anxiety	Australia	Clinic	NR (7–14)	Indicated	Active	Group
Barrett 2005	Anxiety	Australia	Clinic	11.9 (7–17)	Indicated	Active	Individual
Barrington 2005	Anxiety	Australia	Clinic	10.0 (7–14)	Indicated	TAU	Mixed
Bayer 2018	Anxiety	Australia	Community	4.6 (4–5)	Selective	TAU	Group
Beardslee 2013^a^	Depression	USA	Clinic	14.8 (13–17)	Indicated	TAU	Group
Bernal 1980^a^	Conduct Disorder	USA	Clinic	8.4 (5–12)	Indicated	Active	Individual
Bernstein 2008	Anxiety	USA	School	NR (7–11)	Indicated	Waitlist	Group
Bjorseth 2016	Conduct Disorder	Norway	CAMHS	5.8 (2–8)	Indicated	TAU	Individual
Burke 2015^a^	Conduct Disorder	USA	Clinic	8.5 (NR)	Indicated	Attentional	Mixed
Butler 2011	Conduct Disorder	UK	Community	15.1 (NR)	Indicated	TAU	Individual
Cartwright-Hatton 2011	Anxiety	UK	Clinic	6.6 (3–9)	Indicated	Waitlist	Group
Cavell & Hughes 2000	Conduct Disorder	USA	School, Home	7.6 (NR)	Selective	Attentional	Mixed
Clark 1994^a^	Conduct Disorder	USA	Community	NR (7–15)	Selective	TAU	Individual
Clark 2010	Substance Misuse	USA	School	16.7 (NR)	Selective	TAU	Mixed
Clarke 1995	Depression	USA	School	15.3 (NR)	Selective	Attentional	Group
Clarke 2001	Depression	USA	Clinic	14.6 (13–18)	Indicated	TAU	Group
Clarke 2002	Depression	USA	Clinic	15.3 (13–18)	Indicated	TAU	Group
Clarke 2016	Depression	USA	Clinic	14.6 (12–18)	Indicated	TAU	Individual
Cobham 1998	Anxiety	Australia	Clinic	9.6 (7–14)	Indicated	Active	Group
Cohen 2005	PTSD	USA	Clinic	11.1 (8–15)	Indicated	Active	Individual
Conrod 2010	Substance Misuse	UK	School	14.0 (13–16)	Selective	Waitlist	Group
Conrod 2011/Conrod 2008	Substance Misuse	UK	School	14.0 (13–16)	Selective	Waitlist	Group
Creswell 2015.1^a^	Anxiety	UK	Clinic	10.2 (7–12)	Indicated	Active	Individual
Creswell 2015.2^a^	Anxiety	UK	Clinic	10.2 (7–12)	Indicated	Active	Individual
Cunningham 2012.1^a^	Conduct Disorder, Substance Misuse	USA	Clinic	16.8 (14–18)	Selective	Attentional	Individual
Cunningham 2012.2^a^	Conduct Disorder, Substance Misuse	USA	Clinic	16.8 (14–18)	Selective	Attentional	Individual
Dakof 2015	Substance Misuse	USA	Youth offending services	16.0 (13–18)	Indicated	Active	Individual
Damico 2018	Substance Misuse	USA	Clinic	16.0 (12–15)	Indicated	TAU	Individual
Deblinger 1999.1	PTSD	USA	Clinic	9.9 (7–13)	Indicated	TAU	Individual
Deblinger 1999.2	PTSD	USA	Clinic	9.9 (7–13)	Indicated	TAU	Individual
Deblinger 2006	PTSD	USA	Clinic	10.8 (8–14)	Indicated	Active	Individual
Dishion 1995	Conduct Disorder	USA	Clinic	12.0 (10–14)	Indicated	Active	Group
Duong 2016	Depression	USA	School	12.8 (12–14)	Indicated	Active	Group
Estrada 2019	Substance Misuse	USA	Online	13.6 (NR)	Selective	TAU	Group
Flannery-Schroeder 2005	Anxiety	USA	Clinic	NR (8–14)	Indicated	Active	Individual
Foa 2013	PTSD	USA	Clinic	15.3 (13–18)	Indicated	Active	Individual
Forgatch 1999	Conduct Disorder	USA	Clinic	7.8 (6–10)	Selective	Waitlist	Group
Garcia-Lopez 2014	Anxiety	Spain	School	15.4 (13–18)	Indicated	Active	Group
Ghaderi 2018	Conduct Disorder	Sweden	Online	NR (10–13)	Indicated	Active	Individual
Godley 2010^a^	Substance Misuse	USA	NR	15.9 (12–18)	Indicated	Active	Individual
Godley 2014	Substance Misuse	USA	Community	15.7 (12–18)	Indicated	TAU	Individual
Goodyer 2017.1	Depression	UK	Clinic	15 (11–17)	Indicated	Active	Individual
Goodyer 2017.2	Depression	UK	Clinic	15 (11–17)	Indicated	Active	Individual
Goossens 2016	Conduct Disorder	Netherlands	School	14.0 (NR)	Selective	Waitlist	Group
Gowers 2007	Eating Disorders	UK	Clinic	14.1 (12–18)	Indicated	TAU	Individual
Hagen 2011	Conduct Disorder	Norway	Community	8.4 (4–12)	Indicated	TAU	Individual
Halldorsdottir 2016	Anxiety	N America	Clinic	9.1 (7–16)	Indicated	Active	Individual
Hautmann 2018	Conduct Disorder	Germany	Home	7.7 (4–11)	Indicated	Active	Individual
Humayun 2017	Conduct Disorder	UK	Agency	15.0 (NR)	Indicated	TAU	Individual
Hurlbert 2013	Conduct Disorder	USA	Community	4.7 (NR)	Selective	TAU	Group
Jouriles 2009^a^	Conduct Disorder	USA	Community	NR (4–9)	Indicated	TAU	Individual
Kazdin 1992	Conduct Disorder	USA	Clinic	10.3 (7–13)	Indicated	Active	Individual
Kendall 2008.1	Anxiety	USA	Clinic	10.3 (7–14)	Indicated	Attentional	Individual
Kendall 2008.2	Anxiety	USA	Clinic	10.3 (7–14)	Indicated	Attentional	Individual
Lammers 2015^a^	Substance Misuse	Netherlands	School	14.0 (12–16)	Selective	Waitlist/no treatment	Group
Larsson 2009	Conduct Disorder	Norway	Clinic	6.6 (4–8)	Indicated	Active	Group
Le Grange 2015	Eating Disorders	USA	Clinic	15.8 (12–18)	Indicated	Active	Individual
Le Grange 2016	Eating Disorders	Australia	Clinic	15.5 (12–18)	Indicated	Active	Individual
Lee 2016	Anxiety	USA	School	9.0 (7–11)	Indicated	Waitlist	Group
Letourneau 2013	Conduct Disorder	USA	Community	14.7 (11–17)	Indicated	TAU	Individual
Lewinsohn 1990	Depression	USA	Clinic	16.2 (14–18)	Indicated	Active	Group
Liddle 2001	Substance Misuse	USA	Clinic	15.9 (13–18)	Indicated	Active	Individual
Liddle 2008	Substance Misuse	USA	Clinic	15.4 (12–18)	Indicated	Active	Individual
Lochman 2004.1	Conduct Disorder	USA	School	(9–11)	Indicated	No treatment control	Group
Lochman 2004.2	Conduct Disorder	USA	Community	(9–11)	Indicated	No treatment control	Group
Lochman 2014	Conduct Disorder	USA	School	10.7 (9–12)	Selective	TAU	Group
Lochman 2015	Conduct Disorder	USA	School	10.2 (9–12)	Selective	Active	Individual
Lock 2010	Eating Disorder	USA	Clinic	14.4 (12–18)	Indicated	Active	Individual
Mahu 2015	Substance Misuse	England	School	13.7 (0.33)	Selective	TAU	Group
Mannarino 2012/Deblinger 2011	PTSD	USA	Clinic	7.7 (4–11)	Indicated	Active	Individual
Mannassis 2010	Anxiety, Depression	Canada	School	NR (8–12)	Selective	Attentional	Group
McGrath 2011^a^	Conduct Disorder, Anxiety	Canada	Home	7.5 (3–12)	Indicated	TAU	Individual
Newton 2016	Substance Misuse	Australia	School	13.4 (13–14)	Selective	TAU	Group
Ogden 2006	Conduct Disorder	Norway	Community	15.1 (12–17)	Indicated	TAU	Individual
Olivares 2014	Anxiety	Spain	School	15.4 (14–18)	Indicated	Active	Group
Olivares-Olivares 2008	Anxiety	Spain	School	15.3 (14–18)	Indicated	Active	Mixed
Olthius 2018	Conduct Disorder	Canada	Home	8.5 (6–12)	Indicated	TAU	Individual
O'Shea 2015	Depression	Australia	Clinic or school counselling facilities	15.3 (13–19)	Indicated	Active	Individual
Ost 2001	Anxiety	Sweden	Community	11.7 (7–17)	Indicated	Active	Individual
Ost 2015	Anxiety	Sweden	NR	11.6 (8–14)	Indicated	Active	Mixed
Pella 2017	Anxiety	USA	Clinic	8.7 (6–13)	Selective	Attentional	Individual
Poppelaars 2016	Depression	Netherlands	School, Computer	13.4 (11–16)	Indicated	Waitlist	Mixed
Rasing 2018	Depression, Anxiety	Netherlands	NR	12.9 (11–15)	Indicated	Waitlist	Group
Robin 1995	Eating Disorder	USA	Clinic	14.1 (11–20)	Indicated	Active	Individual
Robin 1999	Eating Disorder	USA	Clinic	14.3 (12–19)	Indicated	Active	Individual
Rohde 2004	Depression, Conduct Disorder	USA	Clinic	15.1 (13–17)	Indicated	Active	Group
Rohde 2014	Depression, Substance Misuse	USA	Clinic	16.2 (13–18)	Indicated	Active	Mixed
Rohde 2015.1	Depression	USA	School	15.5 (13–19)	Indicated	Attentional	Group
Rohde 2015.2	Depression	USA	School	15.5 (13–19)	Indicated	Attentional	Individual
Ruggiero 2015	PTSD	USA	Computer	14.5 (12–17)	Indicated	Attentional	Individual
Salerno 2016	Eating Disorders	UK	Clinic	16.9 (12–21)	Indicated	TAU	Individual
Salloum 2012	PTSD	USA	School	9.6 (6–12)	Indicated	Active	Group
Salzer 2018	Anxiety	Germany	Clinic	17.4 (14–20)	Indicated	Active	Individual
Sandler 2019	Conduct Disorder, Depression	USA	Community	NR (3–18)	Selective	Active	Group
Santacruz 2006.1	Anxiety	Spain	Home	6.5 (4–8)	Indicated	Waitlist	Individual
Santacruz 2006.2	Anxiety	Spain	Home	6.5 (4–8)	Indicated	Waitlist	Individual
Saulsberry 2013	Depression	USA	Clinic	17.3 (14–21)	Indicated	Active	Individual
Schaeffer 2014	Substance Misuse	USA	Community	15.8 (15–18)	Selective	TAU	Group
Schneider 2013	Anxiety	Germany	Clinic	10.4 (8–13)	Indicated	Active	Individual
Scott 2010	Conduct Disorder	UK	School	5.5 (5–6)	Indicated	TAU	Group
Sheffield 2006	Depression	Australia	School	14.3 (13–15)	Indicated	TAU	Group
Silk 2018 ^a^	Anxiety	USA	NR	11.0 (9–14)	Indicated	Active	Individual
Silverman 1999.1	Anxiety	USA	Clinic	9.9 (6–16)	Indicated	Attentional	Individual
Silverman 1999.2	Anxiety	USA	Clinic	9.9 (6–16)	Indicated	Attentional	Individual
Silverman 2009	Anxiety	USA	Clinic	9.9 (7–16)	Indicated	Active	Individual
Simon 2011.1	Anxiety	Netherlands	School	9.9 (8–13)	Indicated	Waitlist	Group
Simon 2011.2	Anxiety	Netherlands	School	9.9 (8–13)	Indicated	Active	Group
Slesnick 2009.1	Substance Misuse	USA	Community	15.1 (12–17)	Indicated	TAU	Individual
Slesnick 2009.2	Substance Misuse	USA	Clinic	15.1 (12–17)	Indicated	TAU	Individual
Slesnick 2013	Substance Misuse	USA	Community	15.4 (12–17)	Indicated	Active	Individual
Solantaus 2010	Depression	Finland	Community	NR (8–16)	Selective	Active	Individual
Somech 2012	Conduct Disorder	Israel	Community	4.0 (NR)	Selective	TAU	Group
Sourander 2016	Conduct Disorder	Finland	Clinic	4 (4–4)	Indicated	Attentional	Individual
Spence 2000	Anxiety	Australia	Clinic	10.7 (7–14)	Indicated	Active	Group
Spence 2006	Anxiety	Australia	Clinic	10.0 (7–14)	Indicated	Active	Group
Spence 2011	Anxiety	Australia	Clinic	14.0 (12–18)	Indicated	Active	Individual
Spijkers 2013	Conduct Disorder	Netherlands	Clinic	10.6 (5–11)	Indicated	Active	Individual
Spirito 2004	Substance Misuse	USA	Emergency department	15.6 (NR)	Selective	TAU	Individual
Spirito 2011	Substance Misuse	USA	Emergency department	15.0 (13–17)	Selective	Active	Individual
Sportel 2013.1	Anxiety	Netherlands	School	14.1 (13–15)	Selective	Waitlist	Group
Sportel 2013.2	Anxiety	Netherlands	Internet	14.1 (13–15)	Selective	Waitlist	Individual
Stefini 2017^a^	Eating Disorders	Germany	Clinic	18.7 (14–20)	Indicated	Active	Individual
Stewart-Brown 2004	Conduct Disorder	UK	Community	4.6 (2–8)	Indicated	Waitlist	Group
Stice 2010.1	Depression	USA	Mixed (school and reading material)	15.6 (14–19)	Selective	Attentional	Group
Stice 2010.2	Depression	USA	Mixed (school and reading material)	15.6 (14–19)	Selective	Attentional	Group
Stice 2010.3	Depression	USA	Mixed (school and reading material)	15.6 (14–19)	Selective	Attentional	Individual (reading matter)
Stice 2009	Eating Disorders	USA	Clinic	15.7 (14–19)	Selective	Attentional	Group
Stice 2006.1	Eating Disorders	USA	Clinic	17.1 (14–19)	Selective	Waitlist	Group
Stice 2006.2	Eating Disorders	USA	Clinic	17.1 (14–19)	Selective	Waitlist	Group
Stolberg 1994	Anxiety, Depression	USA	School	9.8 (8–12)	Selective	Waitlist	Group
Sussman 2012^a^	Substance Misuse	USA	School	16.8 (14–21)	Selective	TAU	Group
Szapocznik 1989.1	Conduct Disorder	USA	Clinic	9.2 (6–12)	Selective	Attentional	Individual
Szapocznik 1989.2	Conduct Disorder	USA	Clinic	9.2 (6–12)	Selective	Attentional	Individual
Tanofsky-Kraff 2016^a^	Eating Disorders	USA	Clinic	14.5 (12–17)	Selective	Attentional	Mixed
Turner 2014	Anxiety	UK	Clinic	14.4 (11–18)	Indicated	Active	Individual
Van Manen 2004	Conduct Disorder	Netherlands	Clinic	11.2 (9–13)	Indicated	Active	Group
Walker 2016	Substance Misuse	USA	School	15.8 (14–17)	Indicated	Attentional	Individual
Walton 2013.1	Substance Misuse	USA	Clinic	16.3 (12–18)	Selective	TAU	Individual
Walton 2013.2	Substance Misuse	USA	Clinic	16.3 (12–18)	Selective	TAU	Individual
Waters 2009	Anxiety	Australia	Clinic	6.8 (4–8)	Indicated	Active	Group
Webster-Stratton 1984	Conduct Disorder	USA	Clinic	4.7 (NR)	Indicated	Active	Individual
Webster-Stratton 1997	Conduct Disorder	USA	Clinic, School	5.7 (4–7)	Indicated	Active	individual
Webster-Stratton 2004	Conduct Disorder	USA	Clinic	5.9 (4–8)	Indicated	Active	Group
Weiss 1999	Conduct Disorder, Depression	USA	School	10.3 (NR)	Indicated	Attentional	Individual
Weiss 2013	Conduct Disorder	USA	Home	14.5 (13–17)	Indicated	TAU	Individual
Wergeland 2014	Anxiety	Norway	Clinic	11.5 (8–15)	Indicated	Active	Individual
Winters 2014	Substance Misuse	USA	School	16.1 (13–17)	Indicated	Active	Individual
Wood 2009	Anxiety	USA	NR	10.0 (6–13)	Indicated	Active	Individual
Woods 2011	Depression	New Zealand	School	14.0 (N)	Indicated	TAU	Group
Young 2009	Depression	USA	School	13.4 (11–16)	Selective	TAU	Mixed
Young 2012	Depression	USA	School	14.0 (11–17)	Indicated	TAU	Mixed
**Study**	**Mode of delivery (digital, face to face, phone, reading matter)**	**Type of intervention**	**Manualised treatment**	**Fidelity check**	**N**	**Intervention delivery personnel**	**Number of sessions**
Arnarson 2011/2009^a^	Face to face	Group CBT	Yes	No	113	Professional	14/15
Augimeri 2007^a^	Face to face	Multiple interventions	Yes	Yes	24	Paraprofessional	12
August. 2004	Face to face	Multiple interventions	Yes	Yes	327	Paraprofessional	144
Barrett 1996	Face to face	Multiple interventions	Yes	Yes	53	Professional	12
Barrett 1998	Face to face	Multiple interventions	Yes	Yes	34	Professional	12
Barrett 2005	Face to face	Family intervention (CBT)	Yes	Yes	51	Professional	16
Barrington 2005	Face to face	Multiple interventions	Yes	Yes	48	Paraprofessional	12
Bayer 2018	Face to face	Parenting	Yes	No	545	Professional	4
Beardslee 2013^a^	Face to face	Group CBT	Yes	Yes	316	Professional	14
Bernal 1980^a^	Face to face	Parenting intervention	Yes	Yes	24	Professional	10
Bernstein 2008	Face to face	Group CBT	Yes	No	37	Professional	11
Bjorseth 2016	Face to face	Parenting intervention	Yes	Yes	65	Professional	21
Burke 2015^a^	Face to face	Multiple interventions	Yes	No	252	Professional	12
Butler 2011	Face to face	Parenting intervention	Yes	Yes	101	Professional	29
Cartwright-Hatton-2011	Face to face	Parenting intervention	Yes	Yes	67	Professional	10
Cavell & Hughes 2000	Face to face	Multiple interventions	Yes	No	60	Paraprofessional	69
Clark 1994^a^	Face to face	Other	No	No	132	Professional	78
Clark 2010	Face to face	Multiple interventions	Yes	No	1,730	Professional	7
Clarke 1995	Face to face	Group CBT	Yes	Yes	125	Professional	15
Clarke 2001	Face to face	Group CBT	Yes	Yes	94	Professional	15
Clarke 2002	Face to face	Group CBT	Yes	Yes	88	Professional	16
Clarke 2016	Face to face	Individual cognitive and behavioural treatments	Yes	Yes	212	Professional	6
Cobham 1998	Face to face	Multiple interventions	Yes	Yes	20	Professional	14
Cohen 2005	Face to face	Individual cognitive and behavioural treatments	Yes	Yes	82	Professional	12
Conrod 2010	Face to face	Other (personality targeted)	Yes	No	691	Professional	3
Conrod 2011/Conrod 2008	Face to face	Other (personality targeted)	Yes	No	347	Professional	3
Creswell 2015^a^	Face to face	Multiple interventions	Yes	Yes	140	Professional	32
Creswell 2015^a^	Face to face	Multiple interventions	Yes	Yes	140	Professional	32
Cunningham 2012.1^a^	Face to face	Other—motivational interviewing	Yes	No	727	Professional	1
Cunningham 2012.2^a^	Digital	Other—motivational interviewing	Yes	No	727	Professional	1
Dakof 2015	Face to face	Family intervention	Yes	Yes	112	Professional	43
Damico 2018	Face to face	Other: motivational interviewing	No	Yes	294	Paraprofessional	
Deblinger 1999.1	Face to face	Family intervention	Yes	No	33	Professional	12
Deblinger 1999.2	Face to face	Individual cognitive and behavioural treatments	Yes	No	33	Professional	12
Deblinger 2006	Face to face	Multiple interventions	Yes	Yes	180	Professional	12
Dishion 1995	Face to face	Family intervention	Yes	No	53	Professional	24
Duong 2016	Face to face	Group CBT	Yes	Yes	111	Paraprofessional	12
Estrada 2019	Digital	Family CBT	No	Yes		Paraprofessional	12
Flannery-Schroeder 2005	Face to face	Individual cognitive and behavioural treatments	Yes	No	25	Paraprofessional	18
Foa 2013	Face to face	Individual cognitive and behavioural treatments	Yes	Yes	61	Professional	11
Forgatch 1999	Face to face	Parenting intervention	Yes	Yes	168	Paraprofessional	14
Garcia-Lopez 2014	Face to face	Multiple interventions	Yes	Yes	60	Paraprofessional	17
Ghaderi 2018	Digital	Parenting	Yes	Yes		Professional	7
Godley 2010^a^	Face to face	Multiple interventions	Yes	Yes	161	Professional	7
Godley 2014	Face to face	Individual cognitive and behavioural treatments	Yes Yes	No No	223	Professional	10–14
Goodyer 2017.1	Face to face	Individual cognitive and behavioural treatments	Yes	Yes	465	Professional	24–28
Goodyer 2017.2	Face to face	Psychotherapy	Yes	Yes	465	Professional	24–28
Goossens 2016	Face to face	Other—personality-targeted	Yes	No	530	Professional	12
Gowers 2007	Face to face	Multiple interventions	Yes	Yes	102	Professional	26
Hagen 2011	Face to face	Parenting intervention	Yes	Yes	112	Professional	13
Halldorsdottir 2016	Face to face	Individual CBT	Yes	No	83	Professional	1
Hautmann 2018	Reading Material and Phone	Parenting	Yes	No	149	Professional	12
Humayun 2017	Face to face	Family intervention	No	No	111	Professional	12
Hurlbert 2013	Face to face	Parenting intervention	Yes	Yes	378	Paraprofessional	6
Jouriles 2009^a^	Face to face	Parenting intervention	Yes	No	66	Professional	20
Kazdin 1992	Face to face	Multiple interventions	Yes	No	50	Professional	41
Kendall 2008.1	Face to face	Family intervention	Yes	Yes	161	Professional	16
Kendall 2008.2	Face to face	Individual cognitive and behavioural treatments	Yes	Yes	161	Professional	16
Lammers 2015^a^	Face to face	Other—personality-targeted	Yes	No	696	Professional	2
Larsson 2009	Face to face	Multiple interventions	Yes	No	106	Professional	30
Le Grange 2015	Face to face	Family intervention	Yes	No	109	Professional	18
Le Grange 2016	Face to face	Parenting intervention	Yes	No	106	Professional	16
Lee 2016	Face to face	Group CBT	Yes	No	61	Professional	9
Letourneau 2013	Face to face	Parenting intervention	Yes	Yes	124	Professional	NR
Lewinsohn 1990	Face to face	Multiple interventions	Yes	Yes	40	Paraprofessional	7
Liddle 2001	Face to face	Family intervention	Yes	No	61	Professional	16
Liddle 2008	Face to face	Family intervention	Yes	Yes	224	Professional	17
Lochman 2004.1	Face to face	Group CBT	Yes	No	183	Paraprofessional	16–33 (depending on whether child only or child and parent)
Lochman 2004.2	Face to face	Multiple interventions	Yes	No	183	Paraprofessional	16–33 (depending on whether child only or child and parent)
Lochman 2014	Face to face	Group CBT	Yes	No	241	Paraprofessional	10
Lochman 2015	Face to face	Individual cognitive and behavioural treatments	Yes	No	360	Paraprofessional	32
Lock 2010	Face to face	Family intervention	Yes	No	121	Professional	24
Mahu 2015	Face to face	Other—personality-targeted	Yes	No	2401	Professional	2
Mannarino 2012/Deblinger 2011	Face to face	Individual cognitive and behavioural treatments	Yes	Yes	57	Professional	16
Mannassis 2010	Face to face	Group CBT	Yes	Yes	148	Professional	12
McGrath 2011^a^	Digital, phone and reading material	Parenting intervention	Yes	Yes	243	Paraprofessional	12
Newton 2016	Face to face	Other—personality-targeted	Yes	Yes	344	Professional	2
Ogden 2006	Face to face	Parenting intervention	Yes	Yes	75	Professional	24
Olivares 2014	Face to face	Group CBT	Yes	No	75	Professional	12
Olivares-Olivares 2008	Face to face	Multiple interventions	Yes	No	37	Professional	24
Olthuis 2018	Reading Material and Phone	Parenting	No	Yes	172	NR	14
O'Shea 2015	Face to face	Psychotherapy	Yes	Yes	39	Professional	16
Ost 2001	Face to face	Individual cognitive and behavioural treatments	Yes	Yes	60	Professional	1
Ost 2015	Face to face	Multiple interventions	Yes	No	52	Professional	22
Pella 2017	Face to face	Family intervention	Yes	Yes	136	Professional	8
Poppelaars 2016	Face to face and digital	Group CBT	Yes	Yes	152	Professional	8
Rasing 2018	Face to face	Group CBT	No	Yes	142	Professional	6
Robin 1995	Face to face	Family intervention	Yes	Yes	22	Professional	42
Robin 1999	Face to face	Family intervention	Yes	Yes	36	Professional	42
Rohde 2004	Face to face	Group CBT	Yes	Yes	93	Paraprofessional	16
Rohde 2014	Face to face	Multiple interventions	Yes	Yes	45	Professional	16
Rohde 2015.1	Face to face	Group CBT	Yes Yes	Yes Yes	378	Paraprofessional	6
Rohde 2015.2	Reading matter	Individual cognitive and behavioural	Yes	Yes	378	Paraprofessional	6
Ruggiero 2015	Digital	Psychoeducation skills training	Yes	Yes	496	Professional	NR
Salerno 2016	Digital, reading matter	Parenting	No	No	149	Professional	NR
Salloum 2012	Face to face	Group CBT	Yes	No	64	Professional	12
Salzer 2018	Face to face	Individual cognitive and behavioural treatment	Yes	Yes	108	Professional	25
Sandler 2019	Face to face	Parenting	Yes	Yes	830	Paraprofessional	12
Santacruz 2006	Reading matter	Individual cognitive and behavioural treatments	No	No	78	Paraprofessional	15
Santacruz 2006	Face to face	Individual cognitive and behavioural treatments	No	No	78	Paraprofessional	15
Saulsberry 2013	Face to face, digital	Other—Motivational Interviewing	No	No	83	Professional	1
Schaeffer 2014	Face to face	Other-Employment skills training	No	No	97	Paraprofessional	54
Schneider 2013	Face to face	Multiple interventions	Yes	Yes	42	Professional	16
Scott 2010	Face to face	Parenting intervention	Yes	Yes	172	Paraprofessional	NR
Sheffield 2006	Face to face	Group CBT	Yes	Yes	246	Paraprofessional	8
Silk 2018 ^a^	Face to face	Individual cognitive and behavioural treatments	Yes	Yes	133	Professional	9
Silverman 1999.1	Face to face	Individual cognitive and behavioural treatments	Yes	Yes	81	Professional	10
Silverman 1999.2	Face to face	Individual cognitive and behavioural treatments	Yes	Yes	81	Professional	10
Silverman 2009	Face to face	Individual cognitive and behavioural treatment s	Yes	Yes	70	Paraprofessional	13
Simon 2011.1	Face to face	Group CBT	Yes	Yes	183	Professional	8
Simon 2011.2	Face to face	Parenting	Yes	Yes	183	Professional	8
Slesnick 2009.1	Face to face	Family therapy	Yes	Yes	119	Professional	16
Slesnick 2009.2	Face to face	Family therapy	Yes	Yes	119	Professional	16
Slesnick 2013	Face to face	Individual CBT and behavioural treatment s	Yes	Yes	122	Professional	14
Solantaus 2010	Face to face	Family intervention	Yes	No	106	Professional	6
Somech 2012	Face to face	Parenting intervention	Yes	Yes	209	Professional	14
Sourander 2016	Digital	Parenting intervention	Yes	Yes	464	Paraprofessional	22
Spence 2000	Face to face	Multiple interventions	Yes	No	36	Professional	12
Spence 2006	Face to face	Group CBT	No	Yes	45	Professional	16
Spence 2011	Face to face	Individual CBT	Yes	Yes	88	Professional	10
Spijkers 2013	Face to face	Parenting intervention	Yes	No	67	Paraprofessional	4
Spirito 2004	Face to face	Other -motivational interviewing	Yes	Yes	124	Paraprofessional	1
Spirito 2011	Face to face	Other -motivational interviewing	No	Yes	97	Professional	3
Sportel 2013.1	Face to face	Group CBT	Yes	No	240	Paraprofessional	20
Sportel 2013.2	Digital	Individual cognitive and behavioural treatments	Yes	No	240	Paraprofessional	20
Stefini 2017^a^	Face to face	Individual CBT	Yes	Yes	81	Professional	60
Stewart-Brown 2004	Face to face	Parenting intervention	Yes	No	116	Paraprofessional	10
Stice 2010.1	Face to face	Group CBT	Yes	Yes	341	Professional	6
Stice 2010.2	Face to face	Psychotherapy	Yes	Yes	341	Professional	6
Stice 2010.3	Reading matter	Individual CBT (reading matter)	Yes	No (reading material)	341	Professional	6
Stice 2009	Face to face	Group CBT	No	Yes	306	Paraprofessional	4
Stice 2006.1	Face to face	Group CBT	Yes	Yes	358	Paraprofessional	3
Stice 2006.2	Face to face	Group CBT	Yes	Yes	358	Paraprofessional	3
Stolberg 1994	Face to face	Psychoeducation skills training	Yes	No	52	Paraprofessional	14
Sussman 2012^a^	Face to face	Psychoeducation skills training	Yes	Yes	791	Paraprofessional	12
Szapocznik 1989.1	Face to face	Family intervention	Yes	No	58	Professional	18–19
Szapocznik 1989.2	Face to face	Psychotherapy	Yes	No	58	Professional	18–19
Tanofsky-Kraff 2016^a^	Face to face	Psychotherapy	Yes	Yes	88	Professional	13
Turner 2014	Face to face	Individual CBT	Yes	Yes	72	Professional	14
Van Manen 2004	Face to face	Individual cognitive and behavioural treatments	Yes	No	82	Professional	11
Walker 2016	Face to face	Other—motivational interviewing	No	Yes	231	Professional	24
Walton 2013.1	Face to face	Other—motivational interviewing	Yes	No	338	Paraprofessional	1
Walton 2013.2	Digital	Other—motivational interviewing	Yes	No	338	Paraprofessional	1
Waters 2009	Face to face	Multiple interventions	Yes	Yes	69	Professional	20
Webster-Stratton 1984	Face to face	Parenting intervention	Yes	No	31	Professional	9
Webster-Stratton 1997	Face to face	Parenting intervention	Yes	Yes	48	Professional	23
Webster-Stratton 2004	Face to face	Multiple interventions	Yes	Yes	56	Professional	56
Weiss 1999	Face to face	Psychotherapy	Yes	No	160	Professional	90
Weiss 2013	Face to face	Parenting intervention	Yes	Yes	164	Professional	10
Wergeland 2014	Face to face	Individual cognitive and behavioural treatments	Yes	Yes	178	Professional	10
Winters 2014	Face to face	Multiple interventions	Yes	Yes	236	Professional	3
Wood 2009	Face to face	Family intervention	Yes	Yes	35	Professional	14
Woods 2011	Face to face	Group CBT	Yes	No	24	Paraprofessional	8
Young 2009	Face to face	Psychotherapy	Yes	No	41	Paraprofessional	10
Young 2012	Face to face	Psychotherapy	Yes	No	98	Paraprofessional	8

Note. TAU = treatment as usual

^a^ no change scores available

58 (35%) interventions had a significant CBT component, 48 (29%) were family or parenting based, 12 (7%) were psychoeducation or psychotherapeutic, 28 (17%) were combined interventions, and 19 (11%) were ‘other’. 113 (68%) were led by mental health professionals, 51 (31%) by paraprofessionals (school professionals or non-mental health professionals with intervention-specific training). Length of programmes varied from 1 to 144 sessions (median 12). Over 80% of outcomes measures were either self or parental report. 101 (61%) studies reported a method for assessing treatment fidelity. The most common disorders were conduct disorder (44 studies or 27%) and anxiety disorders (43 studies or 26%). Depressive disorders (29 studies or 18%) and substance misuse (27 studies or 16%) were also relatively common. Less common were eating disorders (12 studies or 7%) and PTSD (9 studies or 5%). The distribution of each study variable differed across disorders (see S1 Table).

### Risk of bias within studies

The methodological quality of the studies as assessed by the Cochrane Risk of Bias tool varied considerably (see S2 Table). Generally, there was a high risk of bias, only 28 studies (20%) had relatively low risk of bias (i.e. high risk of bias in no more than one domain) though a further 70 had high risk of bias estimates in 2 domains. Almost half (47%) of all studies achieved low risk of bias ratings in only 2 or less domains.

### Results of individual studies

**Fig 2:**
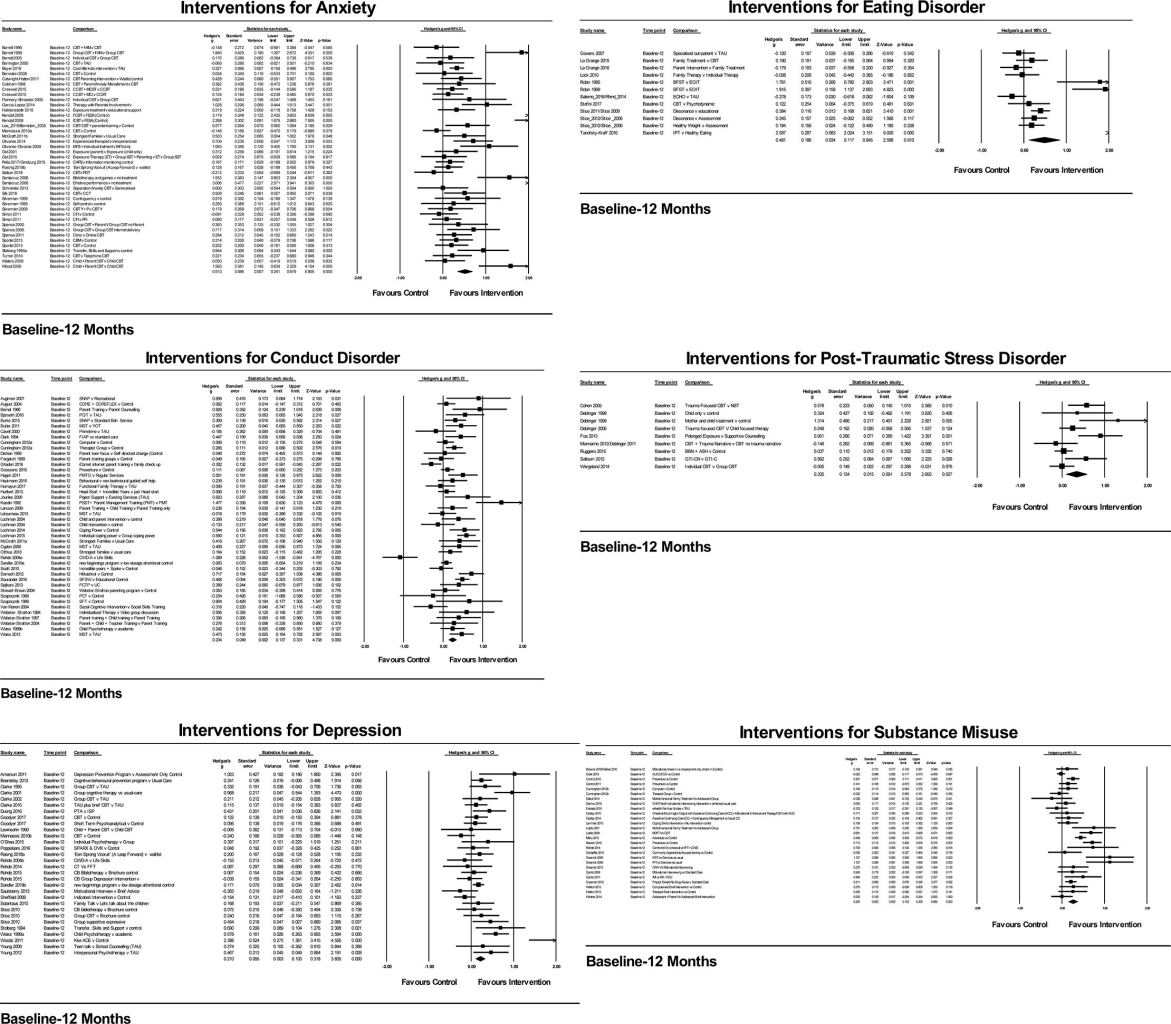
Effects of interventions for each disorder at 12-month follow-up. Where data was nominal, event counts have been added to the change score columns. Where only effect sizes were available, standardized mean differences (d) or odds ratios (OR) were added to the change score columns.

Fig 2 presents the forest plots for each disorder, showing Hedges’ g with 95% confidence intervals for the intervention and control groups at 12-month follow-up.

### Synthesis of results

Meta-analyses were conducted to compare intervention and control groups across all disorders at post-intervention and 12-month follow-up. Overall effect size (ES) post-intervention was moderate (K = 115, g = 0.39; 95% CI: 0.30–0.47 I^2^ = 84.19%, N = 13,982). The overall ES was small to medium at 12 months follow-up (K = 165, g = 0.31, CI: 0.25–0.37, I^2^ = 77.31%, N = 25,652) (see Table 2 and Fig 2). A number of studies only reported 12-month follow-up data (K = 39). Excluding these studies, the ES at 12-month follow-up was slightly but not significantly higher (K = 115, g = 0.36, CI: 0.28–0.43 I^2^ = 78.88%). Across diagnostic groups there were small to medium statistically and clinically important effects at end of intervention (range from g = 0.19, 95% CI:0.01–0.38, I^2^ = 78.47% for substance misuse to g = 0.66, CI: 0.28–1.03 I^2^ = 67.75% for PTSD). These effects were largely maintained at 12-month follow-up with no overall statistically significant decline (range from g = 0.21 CI:0.10–0.32, I^2^ = 61.36% for depression to g = 0.51 CI: 0.34–0.68 I^2^ = 81.29% for anxiety disorders) although there was a more marked decline in the case of depression and PTSD. An overall effect of date of publication was identified with ESs declining for more recent publications for end of intervention (Q = 10.08, df = 2, p = 0.006) and follow-up (Q = 16.92, df = 2, p<0.001; see Table 2). It should also be noted that the I^2^ statistic was generally high throughout these analyses which probably reflects heterogeneity in trial populations and interventions types and supports the exploratory approach we took to sub-group analyses in this review. We also explored whether the heterogeneity in the analyses could be explained by risk of bias by comparing low risk of bias studies (that is, those with 2 or less ratings of “high risk of bias”) with those with higher risk of bias. Across disorders heterogeneity generally remained high, between 60% and 86% in analyses of low risk of bias studies which suggests that risk of bias is not a substantial contributor to heterogeneity in this review. We identified 5 potential studies which might include data on self-harm, of which only 2 reported relevant outcomes at 12 months. These studies were however excluded as the populations in the studies were outside the scope of the review.

**Table 2:** Subgroup analysis at end of treatment and follow-up across all disorders.

		End of Intervention				Follow-up			
		K	G (95% CI)	I^2^	Q(df)	K	G (95% CI)	I^2^	Q(df)
**Population**									
Age									
	Under 12	54	0.45 (0.28–0.63)	87.63		76	0.40 (0.29–0.50)	77.58	
	Over 12	61	0.35 (0.25–0.44)	79.48	1.11 (df = 1) 0.292	89	0.25 (0.18–0.32)	76.41	5.43 (df = 1)* 0.020
Nationality									
	US	71	0.44 (0.33–0.55)	85.36		99	0.35 (0.28–0.43)	79.68	
	Non-US	44	0.30 (0.15–0.44)	81.96	2.39 (df = 1) 0.122	66	0.24 (0.16–0.33)	72.98	3.41 (df = 1) 0.065
Severity									
	Selected	28	0.21 (0.09–0.32)	77.62		44	0.25 (0.16–0.33)	76.49	
	Indicated	87	0.47 (0.35–0.59)	85.14	9.45 (df = 1)** 0.002	121	0.35 (0.27–0.42)	77.43	2.96 (df = 1) 0.085
Disorder									
	Anxiety	36	0.61 (0.34–0.89)	89.71		43	0.51 (0.34–0.68)	81.29	
	Conduct	23	0.20 (0.05–0.35)	76.54		44	0.23 (0.14–0.33)	71.16	
	Depressive	28	0.38 (0.24–0.53)	77.67		30	0.21 (0.10–0.32)	61.36	
	Eating	8	0.49 (0.15–0.84)	84.73		12	0.48 (0.12–0.85)	90.38	
	Post-traumatic stress	6	0.66 (0.28–1.03)	67.75		9	0.34 (0.09–0.58)	66.15	
	Substance	14	0.19 (0.01–0.38)	78.47	13.48 (df = 5)* 0.019	27	0.26 (0.15–0.36)	77.72	11.06 (df = 5)* 0.050
**Intervention**									
Modality									
	Individual CBT / BT	23	0.54 (0.25–0.82)	89.98		31	0.37 (0.21–0.52)	77.62	
	Group CBT	25	0.26 (0.11–0.42)	76.54		27	0.23 (0.07–0.38)	75.85	
	Family-based	18	0.78 (0.41–1.16)	91.21		19	0.68 (0.37–1.00)	88.40	
	Parent training	13	0.33 (0.13–0.52)	83.69		29	0.24 (0.14–0.34)	65.17	
	Psychoeducation/skills	3	0.18 (-0.15–0.51)	0		4	0.49 (0.12–0.86)	61.14	
	Psychotherapy	7	0.44 (0.08–0.79)	81.67		8	0.56 (0.11–1.01)	89.59	
	Multiple intervention	20	0.40 (0.24–0.57)	65.77		28	0.23 (0.10–0.36)	65.18	
	Other	6	-0.12 (-0.27–0.04)	3.34	38.65 (df = 7)** < .0001	19	0.21 (0.12–0.31)	64.74	13.54 (df = 7) 0.060
Format									
	Group or mixed	54	0.33 (0.23–0.43)	77.40		77	0.26 (0.18–0.33)	76.88	
	Individual	61	0.46 (0.30–0.61)	87.64	1.80 (df = 1) 0.180	88	0.36 (0.27–0.45)	77.59	3.19 (df = 1) 0.074
Intensity									
	Low	38	0.28 (0.16–0.40)	79.76		60	0.20 (0.13–0.26)	62.24	
	Moderate	57	0.52 (0.36–0.68)	87.96		74	0.45 (0.34–0.56)	83.93	
	High	20	0.27 (0.12–0.42)	65.28	6.51 (df = 2)* 0.041	31	0.28 (0.15–0.40)	69.40	14.56 (df = 2)**0.001
Manualisation									
	Manualised	99	0.39 (0.30–0.49)	84.16		146	0.31 (0.25–0.37)	77.36	
	Not manualized	16	0.36 (0.10–0.63)	85.07	0.04 (df = 1) 0.847	19	0.30 (0.13–0.46)	78.17	0.02 (df = 1) 0.882
Fidelity Check									
	Absent	44	0.30 (0.16–0.43)	79.14		64	0.30 (0.22–0.38)	72.04	
	Present	71	0.44 (0.32–0.55)	85.69	2.34 (df = 1) 0.126	101	0.31 (0.23–0.39)	79.90	0.04 (df = 1) 0.845
**Design and setting**									
Control type									
	Active	55	0.31 (0.20–0.43)	75.63		64	0.29 (0.19–0.40)	73.88	
	Attentional	21	0.68 (0.34–1.02)	92.60		32	0.53 (0.35–0.70)	88.49	
	TAU	23	0.40 (0.23–0.56)	84.78		44	0.22 (0.14–0.30)	67.20	
	Waitlist/no treatment	16	0.32 (0.09–0.56)	81.43	4.39 (df = 3) 0.222	25	0.26 (0.14–0.38)	66.21	10.00 (df = 3)* 0.019
Setting									
	Clinic	58	0.50 (0.34–0.67)	86.28		78	0.38 (0.27–0.49)	81.98	
	Community	25	0.30 (0.13–0.43)	82.53		41	0.23 (0.14–0.33)	66.62	
	School	30	0.32 (0.18–0.46)	79.58	9.29 (df = 2) 0.026*	43	0.28 (0.19–0.38)	74.96	4.28 (df = 2) 0.233
Agent									
	Professional	80	0.40 (0.28–0.53)	86.33		113	0.35 (0.27–0.42)	78.83	
	Paraprofessional	34	0.35 (0.23–0.47)	76.25	1.64 (df = 1) 0.440	51	0.23 (0.14–0.32)	73.62	4.65 (df = 1) 0.098
Date									
	1985–1999	20	0.46 (0.25–0.67)	61.69		23	0.58 (0.37–0.80)	70.07	
	2000–2009	37	0.62 (0.39–0.84)	90.16		44	0.48 (0.3–0.65)	85.48	
	2010–2018	58	0.25 (0.16–0.35)	79.50	10.07 (df = 2)** 0.006	98	0.22 (0.16–0.27)	69.73	16.92 (df = 2)** 0.000

Analyses of between-group differences identified a number of potential associations (see Table 2). In particular, at follow-up interventions for under 12 years of age, anxiety and eating disorders and interventions of moderate intensity had higher, but not significantly so, ESs.

Greater specificity was achieved when studies of specific diagnostic groups were analysed separately. Analyses at 12-month follow-up are shown in Tables 3 and 4. Analyses at end of treatment are provided in S3A and S3B Table. For conduct disorders outcomes were maintained at follow-up (g = 0.23 95% CI 0.14–0.33, see Table 2). Group-based CBT was associated with negative outcomes (g = -0.27, 95% CI -1.87–1.33) and mixed group and individual interventions were somewhat worse than individual treatments Q_B_(1) = 6.93, *p* = .008). For CD professionals may do better, although not significantly, than paraprofessionals (professional: g = 0.32, 95% CI 0.18–0.47) paraprofessional: g = 0.15, 95% CI 0.01–0.37; Q_B_(1) = 3.03, *p* = .220)).

**Table 3:** Subgroup analysis at follow-up for conduct and substance disorders.

		Conduct Disorder				Substance Misuse			
		K (N = 7,728)	G (95% CI)	I^2^	Q(df)	K (N = 10,546)	G (95% CI)	I^2^	Q(df)
**Population**									
Age									
	Under 12	31	0.31 (0.20–0.42)	61.07					
	Over 12	11	0.12 (-0.08–0.32)	77.48	5.89 (df = 1)	27	0.26 (0.15–0.36)	77.72	0 (df = 0)
Nationality									
	US	28	0.25 (0.12–0.38)	71.84		22	0.23 (0.11–0.34)	72.31	
	Non-US	16	0.22 (0.07–0.37)	71.79	0.09 (df = 1)	5	0.34 (0.16–0.53)	80.01	1.04 (df = 1)
Severity									
	Selected	11	0.22 (0.10–0.35)	62.39		14	0.18 (0.07–0.30)	74.85	
	Indicated	33	0.24 (0.11–0.37)	73.74	0.03 (df = 1)	13	0.36 (0.18–0.54)	75.78	2.62 (df = 1)
**Intervention**									
Modality									
	Individual CBT / BT	3	0.24 (-0.3–0.77)	84.61		2	0.2 (-0.03–0.44)	0	
	Group CBT	2	-0.27 (-1.87–1.33)	96.62					
	Family-based	3	0.07 (-0.27–0.4)	17.71		6	0.53 (0.06–1.00)	87.90	
	Parenting training	22	0.28 (0.15–0.40)	67.00					
	Psychoeducation/skills					1	0.21 (0.08–0.35)	0	
	Psychotherapy	2	0.15 (-0.23–0.52)	16.12					
	Multiple intervention	8	0.29 (0.01–0.57)	70.87		4	0.07 (-0.12–0.27)	50.80	
	Other	4	0.18 (0.04–0.31)	33.19	2.62 (df = 6)	14	0.24 (0.13–0.35)	66.78	3.88 (df = 4)
Format									
	Group or mixed	19	0.10 (-0.04–0.23)	71.38		11	0.23 (0.08–0.38)	84.46	
	Individual	25	0.35 (0.22–0.47)	64.88	6.93 (df = 1)**	16	0.29 (0.14–0.44)	71.09	0.34 (df = 1)
Intensity									
	Low	10	0.24 (0.05–0.42)	70.09		15	0.21 (0.09–0.33)	78.46	
	Moderate	18	0.16 (-0.05–0.32)	74.44		8	0.53 (0.25–0.82)	82.99	
	High	16	0.32 (0.17–0.47)	62.16	2.02 (df = 2)	4	0.06 (-0.12–0.24)	0	7.58 (df = 2)*
Manualisation									
	Manualised	38	0.22 (0.12–0.33)	72.45		24	0.29 (0.18–0.40)	78.92	
	Not manualized	6	0.30 (0.12–0.49)	46.00	0.52 (df = 1)	3	0.12 (-0.11–0.18)	7.17	7.15 (df = 1)**
Fidelity Check									
	Absent	23	0.25 (0.13–0.37)	58.66		11	0.19 (0.06–0.33)	76.33	
	Present	21	0.22 (0.07–0.37)	78.88	0.08 (df = 1)	16	0.31 (0.16–0.47)	78.22	1.32 (df = 1)
**Design and setting**									
Control type									
	Active	13	0.20 (-0.07–0.47)	85.64		8	0.33 (0.08–0.59)	71.18	
	Attentional	7	0.31 (0.12–0.49)	54.74		8	0.41 (0.14–0.67)	83.96	
	TAU	18	0.30 (0.18–0.43)	49.88		9	0.14 (0.00–0.28)	77.74	
	Waitlist/no treatment	6	0.08 (-0.03–0.19)	0	8.29 (df = 3)*	2	0.23 (0.10–0.36)	0	3.95 (df = 3)
Setting									
	Clinic	18	0.29 (0.08–0.49)	79.77		11	0.32 (0.12–0.52)	74.63	
	Community	19	0.16 (0.06–0.27)	47.93		7	0.25 (0.00–0.50)	67.89	
	School	7	0.26 (0.06–0.47)	71.82	1.49 (df = 2)	9	0.22 (0.07–0.37)	86.09	0.55 (df = 2)
Agent									
	Professional	24	0.32 (0.18–0.47)	67.71		19	0.32 (0.18–0.46)	83.39	
	Paraprofessional	19	0.15 (0.01–0.29)	76.16	3.03 (df = 1)	8	0.14 (0.06–0.23)	0	4.53 (df = 1)*
Date									
	1985–1999	10	0.41 (0.14–0.69)	64.17					
	2000–2009	12	0.07 (-0.18–0.32)	74.56		5	0.73 (0.42–1.05)	62.87	
	2010–2018	22	0.26 (0.15–0.37)	70.43	3.37 (df = 2)	22	0.18 (0.09–0.28)	72.72	10.95 (df = 1)**

**Table 4:** Subgroup analysis at follow-up for depressive and anxiety disorders.

		Depressive Disorders				Anxiety Disorders			
		K (N = 6,783)	G (95% CI)	I^2^	Q(df)	K (N = 3,788)	G (95% CI)	I^2^	Q(df)
**Population**									
Age									
	Under 12	3	0.32 (-0.29–0.94)	86.70		34	0.56 (0.35–0.76)	83.79	
	Over 12	26	0.21 (0.09–0.32)	57.97	0.31 (df = 1)	9	0.36 (0.13–0.60)	60.69	1.41 (df = 1)
Nationality									
	US	20	0.23 (0.12–0.35)	50.18		13	0.86 (0.40–1.33)	88.14	
	Non-US	10	0.20 (-0.03–0.43)	72.78	0.07 (df = 1)	30	0.35 (0.20–0.50)	70.51	4.23 (df = 1)*
Severity									
	Selected	9	0.19 (0.04–0.34	33.04		5	0.32 (0.15–0.49)	24.44	
	Indicated	21	0.22 (0.08–0.37)	68.30	0.10 (df = 1)	38	0.54 (0.34–0.75)	83.07	2.74 (df = 1)
**Intervention**									
Modality									
	Individual CBT / BT	4	0.10 (-0.05–0.25)	0		14	0.67 (0.30–1.05)	84.78	
	Group CBT	14	0.26 (0.05–0.47)	76.42		8	0.23 (-0.00–0.47)	55.65	
	Family-based	1	0.17 (-0.21–0.55)	0		4	1.24 (-0.06–2.54)	95.49	
	Parent training	1	0.17 (0.03–0.31)	0		4	0.31 (0.17–0.45)	0	
	Psychoeducation/skills	2	0.55 (0.13–0.98)	0		1	0.94 (0.34–1.54)	0	
	Psychotherapy	5	0.36 (0.15–0.57)	33.20					
	Multiple intervention	2	-0.05 (-0.50–0.40)	0		12	0.35 (0.07–0.62)	66.25	
	Other	1	-0.27 (-0.69–0.16)	0	12.75 (df = 7)				9.62 (df = 5)
Format									
	Group or mixed	21	0.25 (0.11–0.40)	68.13		21	0.35 (0.19–0.51)	62.17	
	Individual	9	0.15 (0.01–0.29)	33.25	1.07 (df = 1)	22	0.67 (0.35–0.99)	87.29	3.18 (df = 1)
Intensity									
	Low	15	0.15 (-0.01–0.31)	58.16		14	0.27 (0.17–0.37)	0	
	Moderate	12	0.28 (0.09–0.46)	65.66		26	0.71 (0.41–1.01)	87.17	
	High	3	0.26 (-0.04–0.55)	67.92	1.13 (df = 2)	3	0.26 (-0.45–0.97)	79.77	7.66 (df = 2)*
Manualisation									
	Manualised	25	0.20 (0.08–0.32)	61.57		39	0.46 (0.30–0.63)	78.92	
	Not manualized	5	0.26 (-0.03–0.56)	62.37	0.16 (df = 1)	4	1.11 (0.05–2.16)	93.11	1.39 (df = 1)
Fidelity Check									
	Absent	10	0.42 (0.14–0.71)	72.28		13	0.65 (0.36–0.94)	77.99	
	Present	20	0.14 (0.04–0.25)	47.97	3.30 (df = 1)	30	0.45 (0.24–0.67)	82.66	1.16 (df = 1)
**Design and setting**									
Control type									
	Active	8	0.11 (-0.04–0.26)	22.40		23	0.39 (0.22–0.56)	59.93	
	Attentional	8	0.35 (0.08–0.63)	74.46		6	1.03 (0.06–2.02)	95.45	
	TAU	10	0.22 (0.01–0.42)	72.34		3	0.31 (0.11–0.50)	12.32	
	Waitlist/no treatment	4	0.22 (0.01–0.43)	8.64	2.50 (df = 3)	11	0.55 (0.21–0.90)	81.68	3.14 (df = 3)
Setting									
	Clinic	9	0.21 (0.05–0.37)	47.53		23	0.54 (0.24–0.84)	84.85	
	Community	4	0.02 (-0.19–0.23)	43.43		8	0.63 (0.27–0.98)	83.78	
	School	16	0.32 (0.12–0.51)	71.39	4.28 (df = 2)	10	0.40 (0.12–0.68)	71.90	2.28 (df = 2)
Agent									
	Professional	19	0.21 (0.09–0.34)	53.78		34	0.44 (0.26–0.62)	80.68	
	Paraprofessional	11	0.22 (0.01–0.43)	71.67	0.01 (df = 1)	9	0.83 (0.36–1.30)	82.16	2.36 (df = 1)
Date									
	1985–1999	4	0.46 (0.23–0.68)	7.23		5	0.66 (0.01–1.31)	77.89	
	2000–2009	5	0.22 (-0.21–0.64)	81.66		16	0.95 (0.48–1.42)	89.13	
	2010–2018	21	0.17 (0.06–0.28)	52.63	5.03 (df = 2)	22	0.23 (0.12–0.34)	28.76	10.04 (df = 2)**

The outcome at follow-up for substance abuse interventions appears promising as there is no observed decline in ES (g = 0.19, 95% CI 0.01–0.38 at end of intervention and g = 0.26, 95% CI 0.15–0.36 at follow up). In substance misuse disorders; family-based interventions (g = 0.53, 95% CI 0.06–1.00) appear to be most effective and effects also appear somewhat stronger for those of moderate intensity (g = 0.53 95% CI 0.25–0.82) and those delivered by professionals (g = 0.32 95% CI 0.18–0.46).

Interventions for anxiety disorders hold up well from end of treatment (g = 0.61 95% CI 0.34–0.89) to follow-up (g = 0.51 95% CI 0.34–0.68) (Table 4). At follow-up individual CBT/BT (g = 0.67 95% CI 0.30–1.05) appears to be associated with larger effects. Moderate intensity interventions (g = 0.71 95% CI 0.41–1.01) appear more effective than interventions of low or high intensity. Effects for interventions delivered by paraprofessionals (g = 0.83 95% CI 0.36–1.30) had a greater but not significant than those delivered by professionals (g = 0.44, 95% CI 0.26–0.62).

For depressive disorders ESs declined post intervention (g = 0.38 95% CI 0.24–0.53) to follow-up (g = 0.21 95% CI 0.10–0.32) but a clinically important effect was still present. With regard to setting, interventions provided in schools (g = 0.32, 95% CI 0.12–0.51) and clinic settings (g = 0.21, 95%CI 0.05–0.37) may be more effective than community settings (g = 0.02, 95%CI -0.19–0.23).

No sub-group analyses were performed for eating disorders or PTSD due to limited study numbers.

### Publication bias

**Fig 3:**
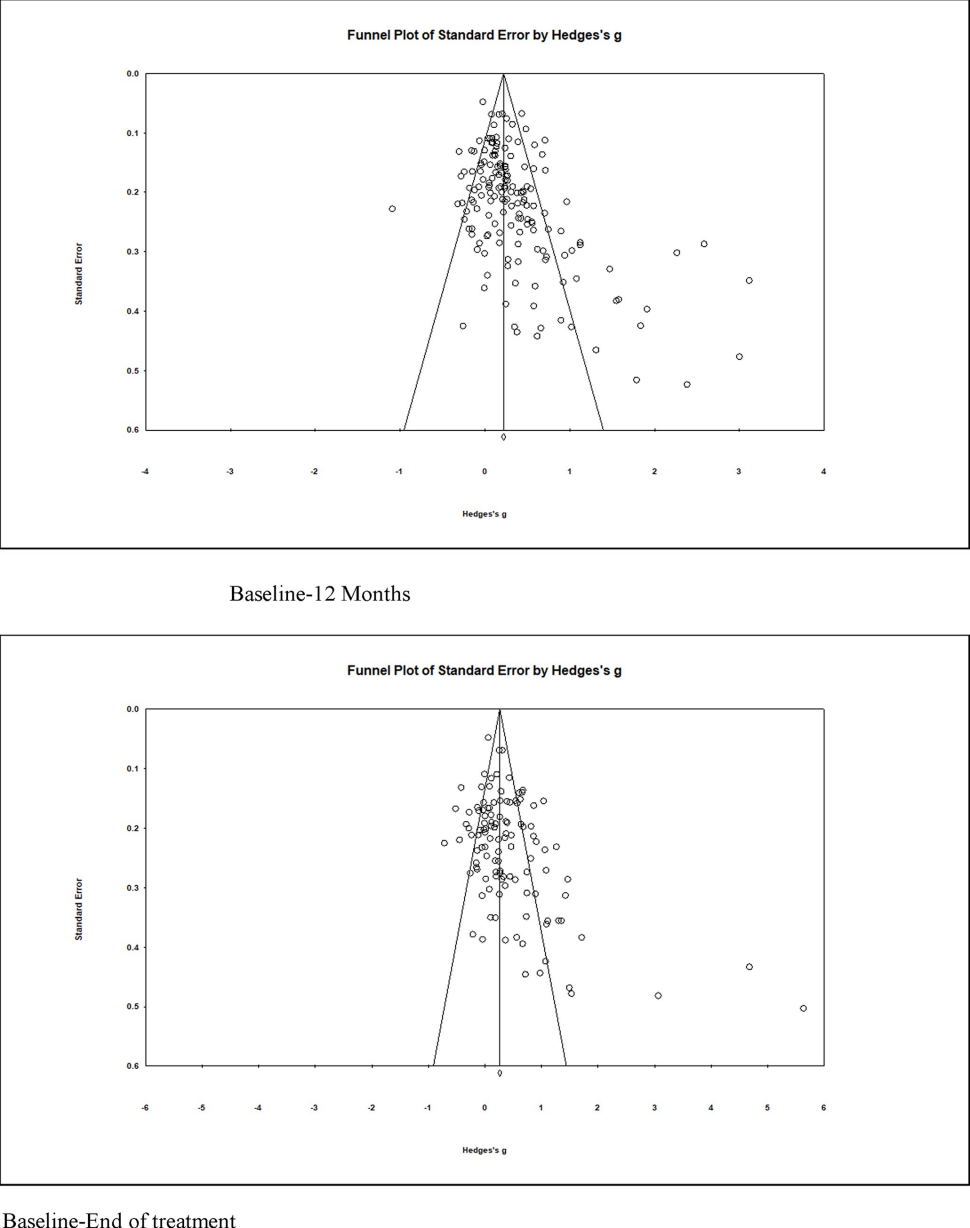
Funnel plots.

The funnel plot for all disorders at follow-up showed evidence of considerable asymmetry indicating publication bias (see Fig 3), which was confirmed by an Egger’s test of bias [23] (1.65, *p* < .001, 95% CI [0.99, 2.30]). It should be noted that the considerable heterogeneity in our analyses may also be a major contributing factor to the asymmetry [24]. When we produced funnel plots for each disorder separately, the asymmetry was less pronounced (Egger’s range: 0.34–1.57, all *p* > .05), with the exception of anxiety (2.74, 95%CI 0.99–4.50, p = 0.003) and depressive disorders (1.40, 95%CI -0.06–2.86, p = 0.060;). Correction for this bias using the trim-and-fill method did not alter the estimates.

## Discussion

This is the first meta-analysis to examine the long-term outcomes of psychosocial interventions for children and young people across most common mental health disorders. The meta-analysis included 138 studies representing 165 comparisons with 12-month follow-up continuous data on psychological interventions. The benefits we identified were typically obtained against standard care or other active treatments and therefore represent additional benefits over that gained from no care, which remains the experience of many children and young people with common mental disorders [25].

Notwithstanding the variability in ES, the heterogeneity in outcomes and the limited number of studies, a broadly consistent picture emerged of sustained, longer-term, and generally small to medium-size benefits against active control interventions. Younger children (under 12) may obtain greater benefit than older children at follow up. There is some indication that interventions delivered by paraprofessionals may be more effective in anxiety disorders equivalent for depression but less effective than those delivered by professionals for conduct disorder and substance misuse. Paraprofessional effectiveness is likely to be enhanced when training programmes are focused on specific interventions, targeted on less severe disorders and supported by appropriate training, continuing supervision and outcome monitoring [26]. Parent training for conduct disorders and family-based interventions for substance misuse appeared effective. There was some evidence to suggest that both family and parenting interventions might be effective in depression and anxiety disorders; given the preponderance of CBT interventions for these disorders consideration should be given to further research and development of these interventions for children and young people with depression and anxiety disorders. Group-based approaches may be effective for depressive and anxiety disorders but may be contra-indicated for conduct disorders. Moderate intensity of intervention appears to be associated with larger effects across all disorders. This resonates with Mulley and colleagues’ view that more care does not necessarily mean better care [27]. Like previous investigations [11], we found that in the school setting indicated interventions appeared as effective as other settings across all disorders. Unlike Brunwasser and colleagues [28] we found no evidence to suggest there may be consistent differences between programmes delivered in schools and those delivered in other settings. The lack of relationship between intervention fidelity to predefined protocols and outcome may be due to the fact that such measures are common to more recent studies, which also have lower ESs associated with improved design. It should also be noted that over 80% of studies included a supervision component which is seen as an essential part of effective psychological practice [29].

This review’s positive picture of long-term benefits is supported by Kodal and colleagues’ recent cohort studies [30] which assessed young men with a range of anxiety disorders for a mean of 3.9 years post treatment and demonstrated maintenance of treatment effects. Some of our included studies reported outcomes beyond 12 months, suggesting that effects were maintained beyond this point, but there were too few to incorporate in the meta-analysis and the likely increased use of intercurrent treatments beyond 12 months complicates both the design and interpretation of long-term follow up studies. Here there is a contrast with psychological and pharmacological interventions for a number of adult disorders, where the effectiveness of treatments across a range of disorders (e.g. depression [31]) show a relapsing and remitting course which is evident at 12-month follow-up.

This review suggests that a modest, persistent effect likely reflects meaningful improvements at population level in ameliorating and preventing the onset of disorders in young people and adults. Meta-analytic studies of prevention programmes support this view [32]. Whilst we know of no other studies that explore the long-term outcome of selective or indicated interventions, the ESs observed are broadly comparable to those in similar reviews focused on short-term outcomes for depression and anxiety [11, 32–34]. This review reinforces the importance of providing effective interventions for children and young people; doing so offers potential long-term benefits which may reduce the burden of mental disorders in adulthood and better enable children and young people in their educational and social worlds which are important in ensuring better mental and physical health. The potential long-term benefits identified by this review provided support for a major national initiative to increase the availability of psychological interventions for children and young people in the English National Health Service (15).

The review has a number of limitations. The high level of heterogeneity in most analyses is a limitation that reflects variability in populations and methods that our exploration of intervention parameters did not capture. It may also reflect some studies’ use of less robust diagnostic measures and inclusion of participants with comorbid disorders. These factors, along with the moderate to high risk of bias characterizing most studies and the evidence of potential publication bias, mandate caution in interpreting the results and greater rigour in the design and reporting of future studies. Baseline severity could not be established due to the wide range of measures and in some cases lack of standardization and again limits the interpretation of these studies. The exclusion of drug interventions led to the exclusion of ADHD and studies for other diagnostic groups which only included drugs as the active comparator. The limitation of studies to those from OECD countries warrants some caution in the interpretation of the results particularly those concerning service delivery systems which might be differently configured in low- and middle-income countries.

Our analyses identify a number of important findings which could be the focus of further research. These include that the interventions could be provided in varying settings, including schools, and that interventions for anxiety and depression may be delivered by professionals or paraprofessionals without diminishing the magnitude of effect, although this may not hold true for substance use and conduct disorders. Importantly, our review suggests that younger children may obtain a greater benefit and that effective parent and family involvement is an important component of effective care. However, it should be noted that these interventions have been provided in the context of protocol-driven and well-supported and supervised care. These are essential aspects of any future research or implementation programme. We did not review any health economic outcomes but further research, and in particular any implementation studies, should consider cost-effectiveness. The absence of sufficient long-term data on self-harm is of particular concern given the high prevalence of this problem in young people, high-quality studies with long-term outcomes are urgently needed. The findings of our review suggests interventions should be provided early, under 12 if possible. It is also important to follow a well-described manual as was the case for most of the studies in this review. As almost all of the studies included supervision of implementers, ensuring effective support and supervision for the interventions may be necessary to achieve the outcomes observed. Future research across all disorders should report long-term outcomes (at least 1 year), including for self-harm and suicide prevention, and given that the effectiveness at end of treatment and follow-up has been established the use of waitlist controls should be discouraged.

Few, if any, systems with these characteristics commonly exist in routine practice and none have been robustly tested. Establishing new models of care and testing these models in large-scale implementation studies would be an important first step.

## Supporting information

S1 Checklist PRISMA 2009 checklist. (DOC)

S1 File Search strategies. (DOCX)

S2 File Extraction and data analysis guidelines. (DOCX)

S3 File Data. (PDF)

S4 File List of reports of studies included in the review. (DOCX)

S1 Table Observed frequencies for each study variable by disorder and associated chi-squared tests. (DOCX)

S2 Table Risk of bias for studies included in the meta-analysis. (DOCX)

S3 Table a. Subgroup analysis at end of intervention for conduct and substance disorders. b. Subgroup analysis at end of intervention for depressive and anxiety disorders.
(DOCX)

S1 Fig Random effects funnel plot for each diagnostic group. (DOCX)

## References

[1] Costello EJ, Copeland W, Angold A. Trends in psychopathology across the adolescent years: what changes when children become adolescents, and when adolescents become adults? Journal of Child Psychology & Psychiatry. 2014;52(10):1015–25. 10.1111/j.1469-7610.2011.02446.x PMC3204367 21815892

[2] Kessler R, Berglund P, Demler O, Jin R, Merikangas K, Walters E. Lifetime Prevalence and Age-of-Onset Distributions of DSM-IV Disorders in the National Comorbidity Survey Replication. Archives of General Psychiatry. 2005;62(6):593–602. 10.1001/archpsyc.62.6.593 15939837

[3] Jones PB. Adult mental health disorders and their age at onset. British Journal of Psychiatry. 2013;202:5–10. 10.1192/bjp.bp.112.119164 23288502

[4] Briggs-Gowan MJ, Owens PL, Schwab-Stone ME, Leventhal JM, Leaf PJ, Horwitz SM. Persistence of psychiatric disorders in pediatric settings. Journal of the American Academy of Child & Adolescent Psychiatry. 2003;42(11):1360–9. 10.1097/01.CHI.0000084834.67701.8a 14566174

[5] Barkmann C, Schulte-Markwort M. Prevalence of emotional and behavioural disorders in German children and adolescents: a meta-analysis. Journal of Epidemiology and Community Health. 2012;66:194–203. 10.1136/jech.2009.102467 20889591

[6] Merikangas KR, He JP, Burstein M. Lifetime prevalence of mental disorders in U.S. adolescents: results from the National Comorbidity Survey Replication—Adolescent Supplement (NCS-A). Journal of the American Academy of Child and Adolescent Psychiatry. 2010;49:980–90. 10.1016/j.jaac.2010.05.017 20855043 PMC2946114

[7] Ahlen J, Lenhard F, Ghaderi A. Universal Prevention for Anxiety and Depressive Symptoms in Children: A Meta-analysis of Randomized and Cluster-Randomized Trials. J Prim Prev. 2015;36(6):387–403. 10.1007/s10935-015-0405-4 26502085

[8] Hodder RK, Freund M, Wolfenden L, Bowman J, Nepal S, Dray J, et al Systematic review of universal school-based 'resilience' interventions targeting adolescent tobacco, alcohol or illicit substance use: A meta-analysis. Prev Med. 2017;100:248–68. 10.1016/j.ypmed.2017.04.003 28390835

[9] Le LK, Barendregt JJ, Hay P, Mihalopoulos C. Prevention of eating disorders: A systematic review and meta-analysis. Clin Psychol Rev. 2017;53:46–58. 10.1016/j.cpr.2017.02.001 28214633

[10] Dray J, Bowman J, Campbell E, Freund M, Wolfenden L, Hodder RK, et al Systematic Review of Universal Resilience-Focused Interventions Targeting Child and Adolescent Mental Health in the School Setting. J Am Acad Child Adolesc Psychiatry. 2017;56(10):813–24. 10.1016/j.jaac.2017.07.780 28942803

[11] Werner-Seidler A, Perry Y, Calear AL, Newby JM, Christensen H. School-based depression and anxiety prevention programs for young people: A systematic review and meta-analysis. Clin Psychol Rev. 2017;51:30–47. 10.1016/j.cpr.2016.10.005 27821267

[12] Neufeld SA, Jones PB, Goodyer IM. Child and adolescent mental health services: longitudinal data sheds light on current policy for psychological interventions in the community. Journal of Public Mental Health. 2017;16(3):96–9. 10.1108/JPMH-03-2017-0013 29721033 PMC5868550

[13] Weisz JR, Kuppens S, Ng MY, Eckshtain D, Ugueto AM, Vaughn-Coaxum R, et al What five decades of research tells us about the effects of youth psychological therapy: A multilevel meta-analysis and implications for science and practice. Am Psychol. 2017;72(2):79–117. 10.1037/a0040360 28221063

[14] Fonagy P, Cottrell D, Phillips J, Bevington D, Glaser D, Allison E. What Works for Whom? A Critical Review of Treatments for Children and Adolescents. New York: Guilford Press; 2014.

[15] Department of Health, Department of Education. Transforming Children and Young People’s Mental Health Provision: a Green Paper: Crown copyright; 2017.

[16] Dunn V, Goodyer IM. Longitudinal investigation into childhood‐and adolescence‐onset depression: Psychiatric outcome in early adulthood. British Journal of Psychiatry. 2006;188:216–22. 10.1192/bjp.188.3.216 16507961

[17] Ginsburg GS, Becker-Haimes E, Keeton C, Kendall PC, Iyengar S, Sakolsky D, et al Results From the Child/Adolescent Anxiety Extended Long-Term Study (CAMELS): Primary Anxiety Outcomes. J Am Acad Child Adolesc Psychiatry. in press. 10.1016/j.jaac.2018.03.017 29960692

[18] Higgins JP, Green S. Cochrane Handbook for Systematic Reviews of Interventions. Chichester: Wiley; 2008.

[19] Duval S, Tweedie R. Trim and Fill: A Simple Funnel-Plot–Based Method of Testing and Adjusting for Publication Bias in Meta-Analysis. Biometrics. 2000;56:455–63. 10.1111/j.0006-341x.2000.00455.x 10877304

[20] Hedges LV, Vevea JL. Fixed-and random-effects models in meta-analysis. Psychological Methods. 1998;3(4):486.

[21] Hedges LV. Distribution theory for Glass' estimator of effect size and related estimators. Journal of Educational Statistics. 1981;6(2):107–28.

[22] Higgins JPT, Thompson SG, Deeks JJ, Altman DG. Measuring inconsistency in meta‐analyses. British Medical Journal. 2003;327(7414):557–60. 10.1136/bmj.327.7414.557 12958120 PMC192859

[23] Egger M, Smith GD, Schneider M, Minder C. Bias in meta-analysis detected by a simple, graphical test. Bmj. 1997;315(7109):629–34. 10.1136/bmj.315.7109.629 9310563 PMC2127453

[24] Sterne JAC, Sutton AJ, Ioannidis JPA, Terrin N, Jones DR, Lau J. Recommendations for examining and interpreting funnel plot asymmetry in meta-analyses of randomised controlled trials. Bmj. 2011;343:d4002 10.1136/bmj.d4002 21784880

[25] Costello EJ, He JP, Sampson NA, Kessler RC, Merikangas KR. Services for adolescents with psychiatric disorders: 12-month data from the National Comorbidity Survey–Adolescent.. Psychiatric Services. 2014;65(3):359–66. 10.1176/appi.ps.201100518 24233052 PMC4123755

[26] Mulley A, Coulter A, Wolpert M, Richards T, Abbasi K. New approaches to measurement and management for high integrity health systems. British Medical Journal. 2017;356:j1401 10.1136/bmj.j1401 28360140

[27] Clark DM. Realizing the mass public benefit of evidence-based psychological therapies: the IAPT program. Annual Review of Clinical Psychology. 2018 5 7;14 10.1146/annurev-clinpsy-050817-084833 29350997 PMC5942544

[28] Brunwasser SM, Garber J. Programs for the Prevention of Youth Depression: Evaluation of Efficacy, Effectiveness, and Readiness for Dissemination. J Clin Child Adolesc Psychol. 2016;45(6):763–83. 10.1080/15374416.2015.1020541 25933173 PMC5176361

[29] Herschell AD, Kolko DJ, Baumann BL, Davis AC. The Role of Therapist Training in the Implementation of Psychosocial Treatments: A Review and Critique with Recommendations. Clinical Psychology Review. 2010;30(4):448–66. 10.1016/j.cpr.2010.02.005 20304542 PMC2872187

[30] Kodal A, Fjermestad K, Bjelland I, Gjestad R, Ost LG, Bjaastad JF, et al Long-term effectiveness of cognitive behavioral therapy for youth with anxiety disorders. J Anxiety Disord. 2018;53:58–67. 10.1016/j.janxdis.2017.11.003 29195188

[31] Vittengl JR, Clark LA, Dunn TW, Jarrett RB. Reducing relapse and recurrence in unipolar depression: a comparative meta-analysis of cognitive-behavioral therapy's effects. Journal of Consulting and Clinical Psychology. 2007;75(3):475–88. 10.1037/0022-006X.75.3.475 17563164 PMC2630051

[32] Stockings EA, Degenhardt L, Dobbins T, Lee YY, Erskine HE, Whiteford HA, et al Preventing depression and anxiety in young people: a review of the joint efficacy of universal, selective and indicated prevention. Psychol Med. 2016;46(1):11–26. 10.1017/S0033291715001725 26315536

[33] Merry SN, Hetrick SE, Cox GR, Brudevold-Iversen T, Bir JJ, McDowell H. Psychological and educational interventions for preventing depression in children and adolescents. Cochrane Database of Systematic Reviews. 2011;7(12):CD003380 10.1002/14651858.CD003380.pub3 22161377

[34] Fisak BJ Jr., Richard D, Mann A. The prevention of child and adolescent anxiety: a meta-analytic review. Prev Sci. 2011;12(3):255–68. 10.1007/s11121-011-0210-0 21437675

