# Supplementary material for: Long-term outcomes of psychological interventions on children and young people’s mental health: A systematic review and meta-analysis
Source: PLoS One. 2020 Nov 16;15(11):e0236525. doi: 10.1371/journal.pone.0236525 (PMC7668611; doi:10.1371/journal.pone.0236525)
Supplement: S1 File — (DOCX) [file pone.0236525.s002.docx]

**S1 Fig: Search strategies**

**(An update search was carried out on the 21^st^ May 2019- see PRISMA)**

**The Cochrane Library**, Issue 10 of 12, October 2017

#1 MeSH descriptor: [Feeding and Eating Disorders] this term only

#2 MeSH descriptor: [Anorexia Nervosa] this term only

#3 MeSH descriptor: [Bulimia Nervosa] this term only

#4 MeSH descriptor: [Binge-Eating Disorder] this term only

#5 MeSH descriptor: [Bulimia] this term only

#6 ("eating disorder*" or ` (eat* near/3 mood*) or EDNOS or anorexi* or orthorexi* or bulimi* or diabulimi* or (bing* near/2 (eat* or pure*))):ti,ab,kw

#7 MeSH descriptor: [Mood Disorders] this term only

#8 MeSH descriptor: [Depressive Disorder] this term only

#9 MeSH descriptor: [Depressive Disorder, Major] this term only

#10 MeSH descriptor: [Seasonal Affective Disorder] this term only

#11 MeSH descriptor: [Dysthymic Disorder] this term only

#12 MeSH descriptor: [Depression] this term only

#13 (mood* or depress* or dysthymi* or "affective disorder*" or "affective symptom*"):ti,ab,kw

#14 MeSH descriptor: [Anxiety Disorders] explode all trees

#15 ((child* or adolesc* or teen* or youth or general*) near/2 anxi*):ti,ab,kw

#16 anxiety:ti

#17 ("anxiety disorder*" or "social* anxiety" or phobi* or agoraphobi* or ADNOS or "health anxiety" or hypochondri* or obsess* or compulsi* or panic or PTSD or "post traumatic stress" or "posttraumatic stress" or "stress disorder*" or ((acute or chronic) near/2 stress*) or neurosis or neuroses or neurotic):ti,ab,kw

#18 ((psychological or emotional) near/2 (debrief* or stress* or trauma*)):ti,kw

#19 (school near/2 (refusal or dropout or drop-out)):ti,ab,kw

#20 ((selective or elective) next mutism):ti,ab,kw

#21 MeSH descriptor: [Adjustment Disorders] explode all trees

#22 MeSH descriptor: [Adaptation, Psychological] explode all trees

#23 wellbeing:ti,kw

#24 ((mental* or psychologic*) next (health or well*)):ti,ab,kw

#25 ((psychologic* or school or social) near/2 (adapt* or adjust*)):ti,ab,kw

#26 MeSH descriptor: [Disruptive, Impulse Control, and Conduct Disorders] explode all trees

#27 (gambling or gambler* or trichotillomani* or firesetting or "fire setting" or ((addicti* or impuls* or compulsi*) near/2 (behavi* or disorder*))):ti,ab,kw

#28 MeSH descriptor: [Obsessive Behavior] this term only

#29 MeSH descriptor: [Self-Injurious Behavior] explode all trees

#30 ((self next (injur* or mutilat*)) or suicide* or suicidal or parasuicid* or para-suicid*):ti,ab,kw

#31 MeSH descriptor: [Somatoform Disorders] explode all trees

#32 (somatoform or somatization or somatisation or "medical* unexplained" or MUPS or "body dysmorphi*"):ti,ab,kw

#33 MeSH descriptor: [Attention Deficit and Disruptive Behavior Disorders] explode all trees

#34 MeSH descriptor: [Attention Deficit Disorder with Hyperactivity] this term only

#35 MeSH descriptor: [Conduct Disorder] this term only

#36 (ADHD or ("attention defici*" near/2 disorder*) or "minimal brain dysfunction"):ti,ab,kw

#37 MeSH descriptor: [Child Behavior Disorders] this term only

#38 MeSH descriptor: [Problem Behavior] this term only

#39 MeSH descriptor: [Child Reactive Disorders] this term only

#40 (oppositional near/3 (defian* or disorder*)):ti,ab,kw

#41 ((conduct or behavi* or antisocial or anti-social or dyssocial or emotional* or internalizing or internalising or externalizing or externalising) near/2 (disorder* or problem* or difficult* or disturb* or psychopath*)):ti,ab,kw

#42 ((conduct or behavi* or personalit*) near/2 (aggressi* or agressi* or antisocial or anti-social or dyssocial or defian* or deliquen* or disturb* or disrupt* or internalizing or internalising or externalizing or externalising or problem*)):ti,ab,kw

#43 MeSH descriptor: [Personality Disorders] explode all trees

#44 (BPD or personality disorder*):ti,ab,kw

#45 MeSH descriptor: [Child Development Disorders, Pervasive] this term only

#46 MeSH descriptor: [Autism Spectrum Disorder] this term only

#47 MeSH descriptor: [Autistic Disorder] this term only

#48 MeSH descriptor: [Asperger Syndrome] this term only

#49 (asperger* or autis* or ((kanners or rett or pervasive development) near/2 (child* or disorder? or syndrom* or symptom*)) or "childhood schizophrenia"):ti,ab,kw

#50 MeSH descriptor: [Substance-Related Disorders] explode all trees

#51 addict*:ti,kw

#52 ((alcohol or drug* or substance) near/2 (abus* or misus* or use or user* or depend* or disorder)):ti,ab,kw

#53 ((addict* or dependen* or abuse or abuser or misuse*) near (adinazolam or aerosol* or alcohol* or alprazolam or amphetamin* or anthramycin or anxiolytic* or ativan or barbituat* or bentazepam or benzodiazepin* or bromazepan or brotizolam or buprenorphin* or camazepam or cannabi* or chlordiazepoxid* or cinolazepam or clobazam or clonazepam or clorazepam or clotiazepam or cloxazolam or cocaine* or codeine or crack or crystal or cyprazepam or depressant* or diacetylmorphin* or diazepam* or doxefazepam or ecstasy or estazolam or etizolam or fentanyl or flunitrazepam or flurazepam or flutazoram or flutoprazepam or fosazepam or gases or GHB or girisopam or halazepam or hallucinogen* or haloxazepam or heroin* or hydromorphone or hydroquinone or hypnotic* or inhalant* or ketamin* or ketazolam or librium or loflazepate or loprazolam or lorazepam or lormetazepam or LSD or marihuana* or marijuana* or MDMA or meclonazepam or medazepam or meperidine or mephedrone or mescalin* or metaclazepam or methadone or methamphetamin* or methaqualone or mexazolam or midazepam or midazolam or morphine* or narcotic* or nerisopam or nimetazepam or nitrazepam or nitrites or "nitrous oxide" or "n-methyl-3,4-methylenedioxyamphetamine" or nordazepam or opiate* or opiod* or opium or oxazepam or oxazolam or oxazypam or oxycodone or oxzepam or painkiller* or "pain killer*" or PCP or pethidin* or phencyclidin* or pinasepam or prazepam or propazepam or propoxyphene or psilocybin or psychedelic* or psychoactive* or psychostimulant* or quinazolinone or ripazepam or ritalin or sedative* or serazepin* or solvent* or steroid* or stimulant* or temazepam or tetrazepam or tofisopam or tramadol or triazolam or triflubazam or valium or vicodin)):ti,ab

#54 (("legal high*" or recreational or party or illicit*) next (drug* or substance*)):ti,ab,kw (Word variations have been searched)

#55 MeSH descriptor: [Bereavement] explode all trees

#56 MeSH descriptor: [Divorce] explode all trees

#57 (divorce* or bereav* or grief or grieving):ti,ab,kw

#58 MeSH descriptor: [Social Problems] explode all trees

#59 MeSH descriptor: [Violence] this term only

#60 MeSH descriptor: [Aggression] explode all trees

#61 MeSH descriptor: [Anger] explode all trees

#62 ("disruptive behavi*" or "problem behavi*" or violence or violent or aggression or "aggressive behavi*" or conflict or conflicts or delinquen* or offend* or offence or convict* or criminal or recidivis* or probation or court-adjudicated):ti,ab,kw

#63 MeSH descriptor: [Criminals] this term only

#64 MeSH descriptor: [Criminal Behavior] explode all trees

#65 (((risk or risky) near/2 (youth* or adolesc*)) or "risk taking" or "risk* behavi*"):ti,ab,kw

#66 ((ED or ((accident or emergency) next (department or ward))) and ((drinking or alcohol or drugs or crime or violence) and brief)):ti,ab,kw

#67 ("high risk" near/3 (population* or communit*)):ti,ab,kw

#68 ("Project Head Start" or "Incredible Years" or "Sure Start"):ti,ab,kw

#69 #1 or #2 or #3 or #4 or #5 or #6 or #7 or #8 or #9 or #10 or #11 or #12 or #13 or #14 or #15 or #16 or #17 or #18 or #19 or #20 or #21 or #22 or #23 or #24 or #25 or #26 or #27 or #28 or #29 or #30 or #31 or #32 or #33 or #34 or #35 or #36 or #37 or #38 or #39 or #40 or #41 or #42 or #43 or #44 or #45 or #46 or #47 or #48 or #49 or #50 or #51 or #52 or #53 or #54 or #55 or #56 or #57 or #58 or #59 or #60 or #61 or #62 or #63 or #64 or #65 or #66 or #67

#70 MeSH descriptor: [Child] explode all trees

#71 (child* or boy* or girl* or kids or juvenil* or minors or pediatric* or paediatric* or adolesc* or preadolesc* or pre-adolesc* or pubert* or pubescen* or prepube* or pre-pube* or teen* or (young next (people or patient* or male* or female* or survivor* or offender* or minorit*)) or youth*):ti

#72 ((child* or boy* or girl* or kids or juvenil* or minors or pediatric* or paediatric* or adolesc* or preadolesc* or pre-adolesc* or pubert* or pubescen* or prepube* or pre-pube* or teen* or (young next (people or patient* or male* or female* or survivor* or offender* or minorit*)) or youth*) near (assign* or allocat* or divid* or random* or control* or group or groups)):ab

#73 (child* or adolescen* or pediatric* or paediatric*):so (Word variations have been searched)

#74 MeSH descriptor: [Schools] this term only

#75 (school* or classroom*):ti,kw

#76 ((youth or young) near/2 (camp* or club* or detention or detainee* or group* or refugee* or immigrant*)):ti,ab,kw

#77 MeSH descriptor: [Child, Orphaned] this term only

#78 MeSH descriptor: [Foster Home Care] this term only

#79 (orphans or orphaned or orphanage* or (foster next (care or home*))):ti,ab,kw

#80 (runaway or run-away or truant* or truancy):ti,ab,kw

#81 MeSH descriptor: [Minors] this term only

#82 MeSH descriptor: [Child of Impaired Parents] this term only

#83 #70 or #71 or #72 or #73 or #74 or #75 or #76 or #77 or #78 or #79 or #80 or #81 or #82

#84 #69 and #83

#85 ("Beat* the Blues" or "Blues Begone" or CBT* or bCBT or b-CBT or cCBT or c-CBT or iCBT or i-CBT or BluesBegone or BounceBackNow or "Bounce Back Now" or BrainMaster or "Brain Master" or BrainGame or "Brain Game" or BRAVEOnline or BRAVE-online or "Brave for Teen*" or "Brave for Child*" or caCCBT or CAVE or CaptainsLog or "Captains Log" or CatchIT or Catch-IT or CATTS or "Camp Cope-A-Lot" or CogMed or "Cool Teens" or ecompared or e-compared or FindMe or "Glasgow Steps" or GlasgowSteps or GripOpJeDip or "Grip Op Je Dip" or Interapy or MasterYourMoodOnline or "Master Your Mood" or Mindcheck* or MindReading or "Mind Reading" or "MobileType" or Mobilyze or MoodGym or "Mood Gym" or Moodhelper or "Mood Helper" or NetCope or "Net Cope" or OCFighter or OC-Fighter or PlayAttention or "Play Attention" or Pratenonline or "Praten Online" or RoboMemo or SALUDBN or "SALUD BN" or SmartBrain or "Smart Brain" or SPARX or StressBusters or "Stress Busters" or Stresspac or StudentBodies or "Student Bodies" or "The Journey" or ThisWayUp or This-Way-Up):ti,ab,kw

#86 ("Think Feel Do" or Bebo or Chatbot or Chat-bot or "Club Penguin" or Franktown or Friendster or Habbo or Jabbersmack or hi5 or iTwixie or Orkut or Sweety High or Kidzworld or "Sina Weibo"):ti,ab,kw

#87 MeSH descriptor: [Psychotherapy] explode all trees

#88 MeSH descriptor: [Psychosocial Support Systems] explode all trees

#89 (psychotherap* or psychoeducat* or psycho-educat* or psychosocial or psycho-social or psychoynamic or psychodrama or ((cognitive or behavio*) near/2 (activation or intervention* or contracting or modification or program* or therap* or train* or treat*)) or metacognitive or meta-cognitive or "coping skills" or ((brief or early or short-term or group) near/2 (intervention or therap*)) or counsel* or ((dialectical or interpersonal) near/2 (intervention* or program* or therap* or train* or treat*)) or "dissonance based" or ((aversion or exposure or implosive or distraction or deactivation) next therap*) or "acceptance and commitment" or (motivation* near/2 (interview* or intervention* or program* or therap*)) or remediat* or multisystemic or multi-systemic or multimodal or multi-modal or multidimension* or multi-dimension* or "combined modalit*" or "collaborative care" or ((family or parent or school* or classroom) near/2 (based or cent* or focus* or intervention* or program* or skill* or therap* or train*)) or "family empowerment" or mindfulness or neurofeedback or bibliotherap* or "role play" or music or dance or drama or ((art or play) next therap*) or (problem next (focus or sol*)) or ((support* or nondirective or non-directive or rational emotive) next therap*) or "transactional analysis" or "trauma focus*" or "resilience training" or imagery or relaxation or "attention training" or ((interaction or reciprocity) next (therapy or training)) or (social next training)):ti,ab,kw

#90 (prevent* or promot* or intervention* or program*) .ti.

#91 #85 or #86 or #87 or #88 or #89 or #90

#92 (#84 and #91) or #68

#93 ((antidepress* or anti-depress* or "anti depress*" or MAOI* or RIMA* or "monoamine oxidase inhibit*" or ((serotonin or norepinephrine or noradrenaline or neurotransmitter* or dopamin*) near (uptake or reuptake or re-uptake or "re uptake")) or SSRI* or SNRI* or NARI* or SARI* or NDRI* or TCA* or tricyclic* or tetracyclic* or pharmacotherap* or pharmacolog* or psychotropic* or "drug therapy") not (psychotherap* or psychological or combin*)):ti

#94 ((acupuncture or aromatherap* or homeopath* or diet or ((dietary or oral) near/2 supplement*) or omega-3 or PUFA*) not (psychotherap* or psychological or combin*)):ti

#95 ((schizo* not (childhood)) or mania or manic or pyschosis or psychotic* or bipolar or antipsych* or anti-psycho* or antiepileptic* or anti-epileptic* or beta-blocker*):ti

#96 (("brain injur*" or epilep* or seizure* or "cerebral palsy" or "cystic fibrosis" or "otitis media" or "whooping cough" or "abdominal pain" or "recurrent pain" or "pain management" or ((cancer or non-cancer) near/2 pain) or enuresis or incontinen* or diarrhoea or "urinary tract") not (depress* or anxiety or psychotherap* or psychological)):ti

#97 ((gestational or antenatal or perinatal or postnatal or postpartum or ante-natal or peri-natal or post-natal or post-partum or childbirth or "child birth" or stillbirth or "still birth*" or newborn or baby or babies or caesarean or miscarriage or pregnan* or gravida* or "preterm labour") not (adolesc* or teen*)):ti

#98 (surgery or surgical or sedation or anaesthe* or anesthe* or "needle related" or postoperative or "post operative" or dental or teeth or "oral device*" or appliances):ti,so

#99 (bacteri* or microb* or antimicrob* or anti-microb* or antibiotic* or virus or viral* or antiviral* or anti-viral* or vaccine* or vaccination* or "HIV transmit*" or immunoglobulin* or immunosupressant* or "growth hormone*" or chelation or "oxygen therapy" or corticosteroid* or "oral steroid*"):ti

#100 #93 or #94 or #95 or #96 or #97 or #98 or #99

#101 (#92 not #100) or #68 (n=8483)

CENTRAL (Trials) = 7942

CDSR (Cochrane Reviews) =181

HTA (Technology Assessments) = 36

DARE (Other Reviews) = 257

NHS-EED (Economic Analyses) = 48

Methods Studies = 19

**MEDLINE-Search-Strategy**

Database: Ovid MEDLINE(R) Epub Ahead of Print, In-Process & Other Non-Indexed Citations, Ovid MEDLINE(R) Daily and Ovid MEDLINE(R) <1946 to 3-Nov-2017>

--------------------------------------------------------------------------------

1 randomized controlled trial.pt. (498311)

2 (randomi#ed or randomi#ation or randomi#ing).ti,ab,kf. (571646)

3 (RCT or "at random" or (random* adj3 (assign* or allocat* or control* or crossover or cross-over or design* or divide* or division or number))).ti,ab,kf. (443428)

4 ((control* adj2 (trial or study or group)) and (placebo or waitlist* or wait* list* or ((treatment or care) adj2 usual) or (no intervention* or non intervention* or non-intervention* or without any intervention*))).ti,ab,kf,hw. (164343)

5 ((single or double) adj blind*).ti,ab,kf,hw. (229598)

6 or/1-5 (917302)

7 (((observational or cross-sectional or case) adj2 study) or case series or case report? or retrospective).ti. (387469)

8 (exp Animals/ or Animal Experimentation/ or exp Models, Animal/) not (exp Human Experimentation/ or Humans/) (4686648)

9 ((afrikaans or albanian or arabic or bulgarian or catalan or chinese or croatian or czech or danish or dutch or estonian or farsi iranian or finnish or French or georgian or German or greek or hebrew or hindi or hungarian or italian or japanese or korean or lithuanian or malaysian or nonenglish or norwegian or polish or portuguese or romanian or russian or serbian or serbo croatian or slovak or slovene or spanish or swedish or turkish or ukrainian or urdu) not (english and (afrikaans or albanian or arabic or bulgarian or catalan or chinese or croatian or czech or danish or dutch or estonian or farsi iranian or finnish or French or georgian or German or greek or hebrew or hindi or hungarian or italian or japanese or korean or lithuanian or malaysian or nonenglish or norwegian or polish or portuguese or romanian or russian or serbian or serbo croatian or slovak or slovene or spanish or swedish or turkish or ukrainian or urdu))).lg. (4427971)

10 (editorial or comment or letter).pt. (1675043)

11 (review.ab. and review.pt.) not trial.ti. (717342)

12 (systematic review not (trial or study)).ti. (79305)

13 (meta analysis not randomized controlled trial).pt. (91885)

14 6 not (or/7-13) (696872)

15 CAMH?.ti,ab,kf. (442)

16 (Project Head Start or Incredible Years or Sure Start).ti,ab,kf. (300)

17 (15 or 16) and 14 (124)

18 exp Child/ (1856539)

19 minors/ (2541)

20 (child* or boy* or girl* or kids or juvenil* or minors or p?ediatric* or adolesc* or preadolesc* or pre-adolesc* or pubert* or pubescen* or prepube* or pre-pube* or teen* or (young adj (people or patient* or male* or female* or survivor* or offender* or minorit*)) or youth*).ti,kf. (1149292)

21 (child* or adolescen* or p?ediatric*).jw. (626220)

22 child psychiatry/ or adolescent psychiatry/ (6784)

23 (school* or classroom?).ti. (101927)

24 schools/ (33332)

25 exp Education, Special/ (16109)

26 student dropouts/ or homeless youth/ (2904)

27 adolescent/ and students/ (23961)

28 ((male or female) adj adolescent?).ab. (5746)

29 ((youth or young) adj2 (camp* or club? or detention or detainee? or group? or refugee? or immigrant?)).ti,ab,kf. (10938)

30 child, orphaned/ or orphanages/ or foster home care/ (4421)

31 (orphan? or (foster adj (care or home?))).ti,kf. (5805)

32 (truant? or truancy).ti,ab,kf,hw. (497)

33 or/18-32 (2520184)

34 "feeding and eating disorders"/ or anorexia nervosa/ or binge-eating disorder/ or bulimia nervosa/ or bulimia/ (29774)

35 (eating disorder* or (eat* adj3 mood*) or EDNOS or anorexi* or orthorexi* or bulimi* or diabulimi* or (bing* adj2 (eat* or purg*))).ti,kf. (24580)

36 mood disorders/ or depressive disorder/ or depressive disorder, major/ or dysthymic disorder/ or seasonal affective disorder/ (110198)

37 Depression/ (104218)

38 (mood* or depress* or dysthymi* or affective disorder* or affective symptom*).ti,kf. (165661)

39 anxiety disorders/ or agoraphobia/ or anxiety, separation/ or neurocirculatory asthenia/ or neurotic disorders/ or obsessive-compulsive disorder/ or hoarding disorder/ or panic disorder/ or phobic disorders/ or phobia, social/ (76889)

40 ((child* or adolesc* or teen* or youth or general*) adj2 anxi*).ti,kf. (4608)

41 anxiety.ti. (43811)

42 (anxiety or depress* or mood or phobi*).ab. /freq=3 (180689)

43 (anxiety disorder* or social* anxiety or phobi* or agoraphobi* or ADNOS or health anxiety or hypochondri* or obsess* or compulsi* or panic or PTSD or post traumatic stress or posttraumatic stress or stress disorder* or ((acute or chronic) adj2 stress*) or neurosis or neuroses or neurotic).ti,kf. (69383)

44 ((psychological or emotional) adj2 (debrief* or stress* or trauma*)).ti,kf. (4780)

45 (school adj2 (refusal or dropout or drop-out)).ti,ab,kf. (850)

46 ((selective or elective) adj mutism).ti,ab,kf. (290)

47 "trauma and stressor related disorders"/ or adjustment disorders/ or stress disorders, traumatic/ or battered child syndrome/ or combat disorders/ or psychological trauma/ or stress disorders, post-traumatic/ or stress disorders, traumatic, acute/ (35803)

48 adaptation, psychological/ or emotional adjustment/ or feedback, psychological/ (93665)

49 *Stress, Psychological/ or *Stress, Psychological/th (68113)

50 wellbeing.ti,kf. and (psycholog* or mental).mp. (1289)

51 ((mental* or psychologic*) and (health or well*)).ti,kf. (66837)

52 ((psychologic* or school or social) adj2 (adapt* or adjust*)).ti,kf. (2892)

53 "disruptive, impulse control, and conduct disorders"/ or firesetting behavior/ or gambling/ or trichotillomania/ (8004)

54 impulsive behavior/ or compulsive behavior/ or behavior, addictive/ (17778)

55 (gambling or gambler* or trichotillomani* or firesetting or fire setting or ((addicti* or impuls* or compulsi*) adj2 (behavi* or disorder*))).ti,kf. (14830)

56 Obsessive Behavior/ (1174)

57 self-injurious behavior/ or self mutilation/ or suicide/ or suicidal ideation/ or suicide, attempted/ (61162)

58 ((self adj (injur* or mutilat*)) or suicide* or suicidal or parasuicid* or para-suicid*).ti,kf. (42566)

59 somatoform disorders/ or body dysmorphic disorders/ or conversion disorder/ or factitious disorders/ or hypochondriasis/ or neurasthenia/ (17298)

60 Psychophysiologic Disorders/ (20204)

61 (somatoform or somatization or somatisation or medical* unexplained or MUPS or body dysmorphi*).ti,kf. (3506)

62 *Mental Disorders/pc, th [Prevention & Control, Therapy] (24822)

63 "attention deficit and disruptive behavior disorders"/ or attention deficit disorder with hyperactivity/ or conduct disorder/ or child behavior disorders/ or child development disorders, pervasive/ or asperger syndrome/ or autism spectrum disorder/ or autistic disorder/ or mutism/ or schizophrenia, childhood/ (79849)

64 (ADHD or (attention adj2 (defici* or disorder*)) or hyperactivity or minimal brain dysfunction).ti,kf. (24135)

65 Problem Behavior/ (871)

66 (oppositional adj3 (defian* or disorder*)).ti,kf. (529)

67 ((conduct or behavi* or antisocial or anti-social or dyssocial or emotional* or internali#ing or externali#ing) adj3 (disorder* or problem* or difficult* or disturb* or psychopath*)).ti,kf. (18819)

68 ((conduct or behavi* or personalit*) adj2 (aggressi* or agressi* or antisocial or anti-social or dyssocial or defian* or deliquen* or disturb* or disrupt* or internali#ing or externali#ing or problem* or repetitive)).ti,kf. (14511)

69 personality disorders/ or antisocial personality disorder/ or borderline personality disorder/ or compulsive personality disorder/ or dependent personality disorder/ or passive-aggressive personality disorder/ (34099)

70 (BPD or personality disorder*).ti,kf. (9458)

71 (asperger* or autis* or kanners or rett or pervasive development or childhood schizophrenia or maladjust*).ti,kf. (33341)

72 exp Substance-Related Disorders/ed, rh, th [Education, Rehabilitation, Therapy] (47965)

73 exp *Substance-Related Disorders/ (202050)

74 addict*.ti,kf,hw. (29196)

75 ((alcohol or drug* or substance) adj2 (abus* or misus* or "use" or user* or depend* or disorder)).ti,kf. (68166)

76 ((addict* or dependen* or abuse or abuser or misuse*) adj2 (adinazolam or aerosol* or alcohol* or alprazolam or amphetamin* or anthramycin or anxiolytic* or ativan or barbituat* or bentazepam or benzodiazepin* or bromazepan or brotizolam or buprenorphin* or camazepam or cannabi* or chlordiazepoxid* or cinolazepam or clobazam or clonazepam or clorazepam or clotiazepam or cloxazolam or cocaine* or codeine or crack or crystal or cyprazepam or depressant* or diacetylmorphin* or diazepam* or doxefazepam or ecstasy or estazolam or etizolam or fentanyl or flunitrazepam or flurazepam or flutazoram or flutoprazepam or fosazepam or gases or GHB or girisopam or halazepam or hallucinogen* or haloxazepam or heroin* or hydromorphone or hydroquinone or hypnotic* or inhalant* or ketamin* or ketazolam or librium or loflazepate or loprazolam or lorazepam or lormetazepam or LSD or marihuana* or marijuana* or MDMA or meclonazepam or medazepam or meperidine or mephedrone or mescalin* or metaclazepam or methadone or methamphetamin* or methaqualone or mexazolam or midazepam or midazolam or morphine* or narcotic* or nerisopam or nimetazepam or nitrazepam or nitrites or "nitrous oxide" or n-methyl-3,4-methylenedioxyamphetamine or nordazepam or opiate* or opiod* or opium or oxazepam or oxazolam or oxazypam or oxycodone or oxzepam or painkiller* or pain killer* or PCP or pethidin* or phencyclidin* or pinasepam or prazepam or propazepam or propoxyphene or psilocybin or psychedelic* or psychoactive* or psychostimulant* or quinazolinone or ripazepam or ritalin or sedative* or serazepin* or solvent* or steroid* or stimulant* or temazepam or tetrazepam or tofisopam or tramadol or triazolam or triflubazam or valium or vicodin)).ti. (22182)

77 ((legal high* or recreational or party or street or illicit*) and (drug* or substance*)).ti,kf. (3559)

78 bereavement/ or grief/ (12890)

79 Divorce/ (4765)

80 (divorce* or bereav* or grief or grieving or death).ti,kf. (140668)

81 *social problems/ or *bullying/ or *civil disorders/ or *riots/ or *crime/ or *defamation/ or *theft/ or *dangerous behavior/ or *driving under the influence/ or *homicide/ or *human rights abuses/ or *human trafficking/ or *slavery/ or *illegitimacy/ or *incest/ or *juvenile delinquency/ or *parental death/ or *maternal death/ or *poverty/ or *sex work/ or *runaway behavior/ or *social behavior disorders/ or *general adaptation syndrome/ or *social segregation/ or *race relations/ or *apartheid/ or *desegregation/ or *racism/ or *underage drinking/ or *violence/ or *domestic violence/ or *ethnic violence/ or *ethnic cleansing/ or *physical abuse/ or *rape/ or *terrorism/ or *mass casualty incidents/ or *torture/ or *exposure to violence/ or *"warfare and armed conflicts"/ or *armed conflicts/ or *warfare/ or *psychological warfare/ or *war exposure/ (105933)

82 social problems/th, rh or bullying/th, rh or civil disorders/th, rh or riots/th, rh or crime/th, rh or defamation/th, rh or theft/th, rh or dangerous behavior/th, rh or driving under the influence/th, rh or homicide/th, rh or human rights abuses/th, rh or human trafficking/th, rh or slavery/th, rh or illegitimacy/th, rh or incest/th, rh or juvenile delinquency/th, rh or parental death/th, rh or maternal death/th, rh or poverty/th, rh or sex work/th, rh or runaway behavior/th, rh or social behavior disorders/th, rh or general adaptation syndrome/th, rh or social segregation/th, rh or race relations/th, rh or apartheid/th, rh or desegregation/th, rh or racism/th, rh or underage drinking/th, rh or violence/th, rh or domestic violence/th, rh or ethnic violence/th, rh or ethnic cleansing/th, rh or physical abuse/th, rh or rape/th, rh or terrorism/th, rh or mass casualty incidents/th, rh or torture/th, rh or exposure to violence/th, rh or "warfare and armed conflicts"/th, rh or armed conflicts/th, rh or warfare/th, rh or psychological warfare/th, rh or war exposure/th, rh (2092)

83 psychosocial problems.ti,kf. (543)

84 *Vulnerable Populations/ or Vulnerable Populations/th, rh (3892)

85 aggression/ or agonistic behavior/ or child reactive disorders/ or dangerous behavior/ or escape reaction/ or risk reduction behavior/ or risk-taking/ or social isolation/ (89802)

86 anger/ or rage/ or hate/ or hostility/ (12569)

87 (violence or violent or aggression or aggressive behavi* or anger or hostil* or conflict or conflicts or delinquen* or offend* or offence or convict* or criminal or recidivis* or probation or court-adjudicated).ti,kf. (76970)

88 Criminal Behavior/ (133)

89 (((risk or risky) adj2 (youth* or adolesc*)) or risk taking or risk* behavi*).ti,kf. (10018)

90 ((ED or ((accident or emergency) adj (department or ward))) and (drinking or alcohol or drugs or crime or violence)).ti,kf. (1057)

91 or/34-90 (1352321)

92 (Beat* the Blues or Blues Begone or CBT* or bCBT or b-CBT or cCBT or c-CBT or iCBT or i-CBT or BluesBegone or BounceBackNow or Bounce Back Now or BrainMaster or Brain Master or BrainGame or Brain Game or BRAVEOnline or BRAVE-online or Brave for Teen* or Brave for Child* or caCCBT or CAVE or CaptainsLog or Captains Log or CatchIT or Catch-IT or CATTS or Camp Cope-A-Lot or CogMed or Cool Teens or ecompared or e-compared or FindMe or Glasgow Steps or GlasgowSteps or GripOpJeDip or Grip Op Je Dip or Interapy or MasterYourMoodOnline or Master Your Mood or Mindcheck* or MindReading or Mind Reading or MobileType or Mobilyze or MoodGym or Mood Gym or Moodhelper or Mood Helper or NetCope or Net Cope or OCFighter or OC-Fighter or PlayAttention or Play Attention or Pratenonline or Praten Online or RoboMemo or SALUDBN or SALUD BN or SmartBrain or Smart Brain or SPARX or StressBusters or Stress Busters or Stresspac or StudentBodies or Student Bodies or The Journey or ThisWayUp or This-Way-Up).ti,ab,kf. (25552)

93 (Think Feel Do or Bebo or Chatbot or Chat-bot or Club Penguin or Franktown or Friendster or Habbo or Jabbersmack or hi5 or iTwixie or Orkut or Sweety High or Kidzworld or Sina Weibo).ti,ab,kf. (121)

94 exp psychotherapy/ (188666)

95 psychosocial support systems/ (70)

96 Psychiatric Rehabilitation/ (152)

97 therapy.fs. and treatment outcome.sh. (224627)

98 Combined Modality Therapy/ (172757)

99 Program Evaluation/ (58498)

100 Secondary Prevention/ (19099)

101 (prevent* or promot* or intervention* or program*).ti. (710274)

102 (psychotherap* or psychoeducat* or psycho-educat* or psychosocial or psycho-social or psychoynamic or psychodrama or ((cognitive or behavio*) adj2 (activation or intervention* or contracting or modification or program* or skill? or therap* or train* or treat*)) or metacognitive or meta-cognitive or coping skills or ((brief or early or short-term or group) adj2 (intervention or therap*)) or counsel* or ((dialectical or interpersonal) adj2 (intervention* or program* or therap* or train* or treat*)) or dissonance based or ((aversion or exposure or implosive or distraction or deactivation) adj therap*) or (acceptance and commitment) or (motivation* adj2 (interview* or intervention* or program* or therap*)) or remediat* or multisystemic or multi-systemic or multimodal or multi-modal or multidimension* or multi-dimension* or combined modalit* or collaborative care or ((family or parent or school* or classroom) adj2 (based or cent* or focus* or intervention* or program* or skill* or therap* or train*)) or family empowerment or mindfulness or neurofeedback or bibliotherap* or role play or music or dance or drama or ((art or play) adj therap*) or (problem adj (focus or sol*)) or ((support* or nondirective or non-directive or rational emotive) adj therap*) or transactional analysis or trauma focus* or resilience training or imagery or relaxation or attention training or ((interaction or reciprocity) adj (therapy or training)) or (social adj training)).ti,kf,hw. (538458)

103 "Play and Playthings"/ (8606)

104 *Alcohol Drinking/th [Therapy] (355)

105 or/92-104 (1488864)

106 14 and 33 and 91 and 105 (6518)

107 ((antidepress* or anti-depress* or anti depress* or MAOI* or RIMA* or monoamine oxidase inhibit* or ((serotonin or norepinephrine or noradrenaline or neurotransmitter* or dopamin*) adj3 (uptake or reuptake or re-uptake or re uptake)) or SSRI* or SNRI* or NARI* or SARI* or NDRI* or TCA* or tricyclic* or tetracyclic* or pharmacotherap* or pharmacolog* or psychotropic* or antipsychotic* or antiepileptic or anti-epileptic* or beta-blocker* or drug therapy) not (psychotherap* or psychological or psychoeducat* or psycho-educat* or educational or cognitive or behavio?ral or CBT or combin* or resistan*)).ti. (150338)

108 ((acupuncture or aromatherap* or homeopath* or diet or ((dietary or oral) adj2 supplement*) or omega-3 or PUFA*) not (psychotherap* or psychological or psychoeducat* or psycho-educat* or educational or behavio?ral or combin*)).ti. (90863)

109 ((brain injur* or epilep* or seizure* or cerebral palsy or cystic fibrosis or otitis media or whooping cough or abdominal pain or recurrent pain or pain management or ((cancer or non-cancer) adj2 pain) or enuresis or incontinen* or diarrhoea or urinary tract) not (depress* or anxiety or psychotherap* or psychological or psychoeducat* or psycho-educat* or educational or behavio?ral)).ti. (256187)

110 ((gestational or antenatal or perinatal or postnatal or postpartum or ante-natal or peri-natal or post-natal or post-partum or childbirth or child birth or stillbirth or still birth* or newborn or baby or babies or caesarean or miscarriage or pregnan* or gravida* or preterm labour) not (adolesc* or teen*)).ti. (382118)

111 (surgery or surgical or sedation or anaesthe* or anesthe* or needle related or postoperative or post operative or dental or teeth or oral device* or appliances).ti. (902777)

112 (bacteri* or microb* or antimicrob* or anti-microb* or antibiotic* or virus or viral* or antiviral* or anti-viral* or vaccine* or vaccination* or HIV transmit* or immunoglobulin* or immunosupressant* or growth hormone* or chelation or oxygen therapy or corticosteroid* or oral steroid*).ti. (1070578)

113 or/107-112 (2784819)

114 17 or (106 not 113) (6279)

115 Follow-Up Studies/ (628333)

116 (followup? or follow* up?).ti,ab,kf. (948133)

117 Longitudinal Studies/ (120508)

118 ((treatment adj2 time) or (time adj (effect? or segment?))).ti,ab,kf. (28835)

119 ((result? or outcome? or measur* or effect? or time point? or timepoint? or treatment? or posttreatment? or therap* or baseline) adj3 (12 month? or twelve month? or 13 month? or thirteen month? or 14 month? or fourteen month? or 15 month? or fifteen month? or 16 month? or sixteen month? or 17 month? or seventeen month? or 18 month? or eighteen month? or 19 month? or nineteen month? or 20 month? or twenty month? or 24 month? or twenty four month? or twentyfour month? or 36 month? or thirtysix month? or thirty six month? or 48 month? or fortyeight month? or forty eight month? or 60 month? or sixty month?)).ti,ab,kf. (46329)

120 ((result? or outcome? or measur* or effect? or time point? or timepoint? or treatment? or posttreatment? or therap*) adj2 (1 year or one year? or 2 year? or two year? or 3 year? or three year? or 4 year? or four year? or 5 year? or five year? or 6 year? or six year? or 7 year? or seven year? or 8 year? or eight year? or 9 year? or nine year? or 10 year? or ten year?)).ti,ab,kf. (72223)

121 (("at" or after or later or later than or longer than) adj1 (12 month? or twelve month? or 13 month? or thirteen month? or 14 month? or fourteen month? or 15 month? or fifteen month? or 16 month? or sixteen month? or 17 month? or seventeen month? or 18 month? or eighteen month? or 19 month? or nineteen month? or 20 month? or twenty month? or 24 month? or twenty four month? or twentyfour month? or 36 month? or thirtysix month? or thirty six month? or 48 month? or fortyeight month? or forty eight month? or 60 month? or sixty month? or 1 year or one year? or 2 year? or two year? or 3 year? or three year? or 4 year? or four year? or 5 year? or five year? or 6 year? or six year? or 7 year? or seven year? or 8 year? or eight year? or 9 year? or nine year? or 10 year? or ten year?)).ti,ab,kf. (381579)

122 year? later.ti,ab,kf. (41372)

123 (long* term or maintenance or time or trajector? or posttreatment? or post-treatment?).ti,kf. (473040)

124 ((sustain* adj3 (effect* or impact)) or ((longterm or long* term?) adj3 (asses* or effect* or impact? or outcome?))).ti,ab,kf. (160932)

125 (sessions adj3 (9 month? or nine month? or 10 month? or 10 month? or 11 month? or eleven month? or 12 month? or twelve month? or 18 month? or eighteen month? or 24 month? or twenty four month? or twentyfour month? or 36 month? or thirtysix month? or thirty six month? or 48 month? or fortyeight month? or forty eight month? or 60 month? or sixty month?)).ti,ab,kf. (205)

126 (post intervention? or postvention?).ti,ab,kf. (10874)

127 or/115-126 (2085155)

128 114 and 127 (3239)

129 remove duplicates from 128 (2847)

***************************

**Ovid PsycINFO** <1960 to September Week 4 2017>

Search Strategy (5-Oct-2017):

--------------------------------------------------------------------------------

*[RCT Filter]*

1     (randomi#ed or randomi#ation or randomi#ing).ti,ab,id. (69938)

2     (RCT or "at random" or (random* adj3 (assign* or allocat* or control* or crossover or cross-over or design* or divide* or division or number))).ti,ab,id. (79427)

3     ((control* adj2 (trial or study or group)) and (placebo or waitlist* or wait* list* or ((treatment or care) adj2 usual) or (no intervention* or non intervention* or non-intervention* or without any intervention*))).ti,ab,id,hw. (16319)

4     ((single or double or triple or treble) adj2 (blind* or mask* or dummy)).ti,ab,id. (23677)

5     treatment outcome.md. and "3300".cc. (1961)

6     or/1-5 (119790)

*[Document/Publication Type]*

7     (journal article or retraction).dt. or (journal or peer reviewed journal).pt. (3424077)

8     6 and 7 (108014)

*[Filter to remove unwanted types of study/publication type]*

9     (comment reply or editorial or letter).dt. (179760)

10     (literature review or meta analysis or metasynthesis or systematic review or retrospective study or longitudinal study).md,sh. (290606)

11     ((systematic* adj2 review*) or meta-analys* or metasynthes* or meta-synthes* or ((literature or scoping) adj2 review?)).ti,id. (50983)

12     (observational or cross-sectional or case control* or case series or case stud* or ((retrospective or longitudinal) adj study)).ti,id. (58425)

13     (randomi#ed adj (controlled or treatment or clinical) adj (trials or studies)).ab. (10304)

14     (survey or prevalence or incidence).ti. (45778)

15     (animal not (human and animal)).po. (336688)

16     ((afrikaans or albanian or arabic or bulgarian or catalan or chinese or croatian or czech or danish or dutch or estonian or farsi iranian or finnish or French or georgian or German or greek or hebrew or hindi or hungarian or italian or japanese or korean or lithuanian or malaysian or nonenglish or norwegian or polish or portuguese or romanian or russian or serbian or serbo croatian or slovak or slovene or spanish or swedish or turkish or ukrainian or urdu) not

(english and (afrikaans or albanian or arabic or bulgarian or catalan or chinese or croatian or czech or danish or dutch or estonian or farsi iranian or finnish or French or georgian or German or greek or hebrew or hindi or hungarian or italian or japanese or korean or lithuanian or malaysian or nonenglish or norwegian or polish or portuguese or romanian or russian or serbian or serbo croatian or slovak or slovene or spanish or swedish or turkish or ukrainian or urdu))).lg. (307784)

**17     8 not (or/9-16) (77002)**

*[Age Group: Children or Adolescents]*

18     (child* or boy* or girl* or kids or juvenil* or minors or p?ediatric* or adolesc* or preadolesc* or pre-adolesc* or pubert* or pubescen* or prepube* or pre-pube* or teen* or (young adj (people or patient* or male* or female* or survivor* or offender* or minorit*)) or youth*).ti,id. (579766)

19     (child* or adolescen* or p?ediatric*).jw. (144274)

20     ("100" or "180" or "200").ag. (714770)

21     child psychiatry/ or adolescent psychiatry/ (7852)

22     (school* or classroom?).ti. (134412)

23     schools/ or classrooms/ or elementary schools/ or high schools/ or institutional schools/ or junior high schools/ or middle schools/ (56274)

24     school dropouts/ or runaway behavior/ (2902)

25     "summer camps (recreation)"/ (293)

26     exp extracurricular activities/ or exp after school programs/ (2695)

27     ((youth or young) adj2 (camp* or club? or detention or detainee? or group? or refugee? or immigrant?)).ti,ab,id. (6050)

28     orphans/ or orphanages/ or foster home care/ (1085)

29     (orphan? or (foster adj (care or home?))).ti,id. (4939)

30     or/18-29 (978261)

*[Filter to remove unwanted age groups]*

31     infant development/ or early childhood development/ or neonatal development/ (30375)

32     (early adj (years or child*)).ti,id,hw. (17522)

33     (neonat* or baby or babies or infant or infants or toddler* or nursery or preschool* or pre-school* or kindergarten*).ti,id,hw. (99709)

34     nursery schools/ or nursery school students/ or preschool students/ or kindergartens/ or kindergarten students/ (17669)

35     (undergraduate* or under-graduate* or postgraduate* or post-graduate* or (university adj2 student*)).ti,id,hw. (29199)

36     GRADUATE STUDENTS/ or DENTAL STUDENTS/ or NURSING STUDENTS/ or MEDICAL STUDENTS/ or EDUCATION STUDENTS/ or POSTGRADUATE STUDENTS/ or BUSINESS STUDENTS/ or STUDENTS/ or LAW STUDENTS/ (48474)

37     (("300" or "320") not (("100" or "180" or "200") and ("300" or "320"))).ag. (1415505)

38     ("300" or "320").ag. not (child* or adolescen* or p?ediatric or school*).ti,jw. (1504608)

39     ((adult? or parent* or mother? or father?) not ((depress* adj1 parent*) or (parent* adj (training or program*)) or ((adult? or parent* or mother? or father?) and (family or families or teacher? or death? or counsel* or school? or child* or boy* or girl* or kids or juvenil* or minors or paediatric* or pediatric* or adolesc* or preadolesc* or pre-adolesc* or pubert* or pubescen* or prepube* or pre-pube* or teen* or (young adj (people or patient* or male* or female* or survivor* or offender* or minorit*)) or youth*)))).ti. (110639)

40     (parents of children with or parents of adolescents with).ti. (1291)

41     or/31-40 (1716989)

**42     30 not 41 (640271)**

*[Condition: Common Mental Disorders]*

43     eating disorders/ or anorexia nervosa/ or bulimia/ or hyperphagia/ or kleine levin syndrome/ or pica/ or “purging (eating disorders)"/ (26797)

44     binge eating/ not obes*.ti. (2192)

45     (eating disorder* or EDNOS or anorexi* or orthorexi* or bulimi* or diabulimi* or ((binge and (eat* or purg*)) not (obes* or metabolic syndrome))).ti,id. (26385)

46     (school refusal or ((selective or elective) adj mutism)).ti,ab. (1075)

47     self destructive behavior/ or attempted suicide/ or head banging/ or self inflicted wounds/ or self injurious behavior/ or self mutilation/ or suicide/ (36150)

48     suicide prevention/ (4029)

49     suicidal ideation/ (7291)

50     affective disorders/ (13047)

51     affective psychosis/ (553)

52     bipolar disorder/ or cyclothymic personality/ (24293)

53     major depression/ or anaclitic depression/ or dysthymic disorder/ or endogenous depression/ or postpartum depression/ or reactive depression/ or recurrent depression/ or treatment resistant depression/ (114633)

54     atypical depression/ (188)

55     "depression (emotion)"/ (23897)

56     seasonal affective disorder/ (1013)

57     anxiety disorders/ or acute stress disorder/ or castration anxiety/ or death anxiety/ or generalized anxiety disorder/ or obsessive compulsive disorder/ or panic disorder/ or posttraumatic stress disorder/ or separation anxiety/ (66519)

58     phobias/ or acrophobia/ or agoraphobia/ or claustrophobia/ or ophidiophobia/ or school phobia/ or social phobia/ (12191)

59     "debriefing (psychological)"/ (272)

60     adjustment disorders/ (613)

61     coping behavior/ (43722)

62     adjustment/ or exp emotional adjustment/ or occupational adjustment/ or school adjustment/ or social adjustment/ (48364)

63     emotional trauma/ (14815)

64     chronic stress/ or environmental stress/ or psychological stress/ or social stress/ or stress reactions/ (22693)

65     anxiety/ or computer anxiety/ or mathematics anxiety/ or performance anxiety/ or social anxiety/ or speech anxiety/ or test anxiety/ (62668)

66     Panic Attack/ or Panic/ or Panic Disorder/ (9228)

67     somatoform disorders/ or body dysmorphic disorder/ or hypochondriasis/ or neurasthenia/ or neurodermatitis/ or somatization disorder/ or somatoform pain disorder/ (10881)

68     conversion disorder/ or hysterical paralysis/ or hysterical vision disturbances/ or pseudocyesis/ (1190)

69     somatization/ (2139)

70     factitious disorders/ or Munchausen Syndrome by Proxy/ or Munchausen Syndrome/ (781)

71     compulsions/ or repetition compulsion/ (2311)

72     obsessions/ (1667)

73     obsessive compulsive personality disorder/ (550)

74     Trichotillomania/ (828)

75     gambling/ or pathological gambling/ (6801)

76     neurosis/ or childhood neurosis/ or traumatic neurosis/ (7486)

77     ((self adj (injur* or mutilat*)) or suicide* or suicidal or parasuicid* or para-suicid* or mood disorder* or affective disorder* or (bipolar adj2 (affective or disorder*)) or cyclothymi* or depression or depressive or dysthymi* or neurotic or neurosis or adjustment disorder* or antidepress* or anti-depress* or anxiety disorder* or ADNOS or health anxiety or agoraphobia or obsess* or compulsi* or panic or phobi* or ptsd or posttrauma* or post trauma* or combat or somatoform or somati#ation or medical* unexplained or body dysmorphi* or conversion disorder or hypochondria* or

neurastheni* or hysteria or munchausen or gambling or trichotillomania or anhedoni* or affective symptoms or ((mental* or psychologic*) adj (health or well*))).ti,id. (375069)

78     ((or/43-46) not 41) or ((or/47-77) and 42) (100397)

*[Other Mental Health Conditions]*

79     attention deficit disorder with hyperactivity/ or oppositional defiant disorder/ (19889)

80     (ADHD or (attention defici* adj2 disorder?) or minimal brain dysfunction).ti,id. (23017)

81     (oppositional adj3 (defian* or disorder?)).ti,id. (1426)

82     conduct disorder/ or explosive disorder/ (4221)

83     personality disorders/ or borderline personality disorder/ (16377)

84     (or/79-83) not 41 (28607)

85     ((conduct or behavi* or antisocial or anti-social or dyssocial or emotional* or internali#ing or externali#ing) adj1 (problem? or difficult* or psychopath*)).ti,id. (25899)

86     ((conduct or behavi* or personalit*) adj1 (aggressi* or agressi* or antisocial or anti-social or dyssocial or defian* or deliquen* or disturb* or disrupt* or internali#ing or externali#ing or problem*)).ti,id. (40435)

87     personality disorders/ or borderline personality disorder/ (16377)

88     (BPD or personality disorder?).ti,id. (18714)

89     (or/85-88) and 42 (23696) (23679)

90     autism spectrum disorders/ or autistic thinking/ or rett syndrome/ (36988)

91     (asperger? or autism or autistic or ((kanners or rett or pervasive development) adj (disorder? or syndrom*))).ti,id. (37050)

92     (or/90-91) not 41 (27777)

93     **17 and (78 or 84 or 89 or 92)** (3781)

*[Psychotherapy or Psychopharmacology (broad terms)]*

94     school psychologists/ or school counselors/ (7120)

95     ((((child* or adolescent*) and psychotherapy) or ((cognitive or behavi*) adj2 therapy*) or CBT) and (treat* or prevent* or intervention or effect? or efficacy or effectiveness or compar* or versus)).ti. (4570)

96     child psychotherapy/ or adolescent psychotherapy/ (7226)

97     ("100" or "180" or "200").ag. and ("3310" or "3311" or "3312" or "3313" or "3314" or "3315" or “3340").cc. (35975)

98     (Beat* the Blues or Blues Begone or blended CBT or bCBT or b-CBT or BluesBegone or BounceBackNow or Bounce Back Now or BrainMaster or Brain Master or BrainGame or Brain Game or BRAVEOnline or BRAVE-online or Brave for Teen* or Brave for Child* or caCCBT or CAVE or CaptainsLog or Captains Log or CatchIT or Catch-IT or CATTS or Camp Cope-A-Lot or CogMed or Cool Teens or ecompared or e-compared or FindMe or Glasgow Steps or GlasgowSteps or GripOpJeDip or Grip Op Je Dip or Interapy or MasterYourMoodOnline or Master Your Mood or Mindcheck* or MindReading or Mind Reading or MobileType or Mobilyze or MoodGym or Mood Gym or Moodhelper or Mood Helper or NetCope or Net Cope or OCFighter or OC-Fighter or PlayAttention or Play Attention or Pratenonline or Praten Online or RoboMemo or SALUDBN or SALUD BN or SmartBrain or Smart Brain or SPARX or StressBusters or Stress Busters or Stresspac or StudentBodies or Student Bodies or The Journey or ThisWayUp or This-Way-Up).ti,id. (3591)

99     (The Journey or Think Feel Do or Bebo or Chatbot or Chat-bot or Club Penguin or Franktown or Friendster or Habbo or Jabbersmack or hi5 or iTwixie or Orkut or Sweety High or Kidzworld or Sina Weibo).ti,id. (2860)

100     17 and ((or/94-99) not 41) (2293)

101     **93 or 100** (4432)

*[Filter to remove unwanted concepts]*

102     (((universal* or primary) adj prevent*) not (((universal* or primary) and (select* or indicated or targeted or secondary or tertiary)) adj3 prevent*)).ti. (993)

103     (health promotion not (health promotion and treat*)).ti. (3230)

104     "3233".cc. (56453)

105     (substance adj ("use" or abuse)).ti. (17352)

106     exp alcoholism/ or alcohol abuse/ or exp binge drinking/ or alcohol intoxication/ or alcohol withdrawal/ or underage drinking/ (48429)

107     smoking cessation/ or exp tobacco smoking/ (32221)

108     condoms/ or safe sex/ or exp sexually transmitted diseases/ (44061)

109     exp Sexual Risk Taking/ (7556)

110     (sex* adj (health or behavi* or risk)).ti. (9273)

111     (smoking or tobacco or nicotine or alcohol*).ti. (81764)

112     ((antenatal or ante-natal or postnatal or post-natal or postpartum or post-partum) not (adolescen* or teen*)).ti. (7949)

113     or/102-112 (202896)

114     101 not 113 (4269)

115     limit 114 to yr="1960 - 2018" **(4263)**

***************************

PsycINFO Concept Codes/ Age Field Tags:

“3300”.cc. Health & Mental Health Treatment & Prevention

“3310”.cc. Psychotherapy & Psychotherapeutic Counseling

“3311”.cc. Cognitive Therapy

“3312”.cc. Behavior Therapy & Behavior Modification

“3313”.cc. Group & Family Therapy

“3314”.cc. Interpersonal & Client Centered & Humanistic Therapy

“3315”.cc. Psychoanalytic Therapy

“3340”.cc. Clinical Psychopharmacology

“3233".cc. Substance Abuse & Addiction

“100".ag. Childhood, from birth to 12 yrs

“180".ag. School Age (6 to 12 yrs)

“200”.ag. Adolescence (13 to 17 yrs)

“300”.ag. Adulthood (18 yrs & older)

“320”.ag. Young Adulthood (18 to 29 yrs)

.ti. Title; .ab. Abstract; .id. Key Cocepts; .jw. Journal Word

|  | EBSCO Education Databases (10-Nov-2017): Education Resource Information Center (ERIC) and the British Education Index (BEI (bri)) |
| --- | --- |
| # | Query |
|  | Total (ERIC/BEI de-duplicated) = 1501 |
| S92 | S83 AND S91 |
| S91 | S89 OR S90 |
| S90 | S84 AND S88 |
| S89 | S85 AND S86 AND S87 AND S88 |
| S88 | S69 OR S70 OR S71 OR S72 OR S73 OR S74 OR S75 OR S76 OR S77 OR S78 OR S79 OR S80 OR S81 OR S82 |
| S87 | S65 OR S66 OR S67 OR S68 |
| S86 | S57 OR S58 OR S59 OR S60 OR S61 OR S62 OR S63 OR S64 |
| S85 | (S1 OR S2 OR S3 OR S4 OR S5 OR S6 OR S7 OR S8 OR S9 OR S10 OR S11 OR S12 OR S13 OR S14 OR S15 OR S16 OR S17 OR S18 OR S19 OR S20 OR S21 OR S22 OR S23 OR S24 OR S25 OR S26 OR S27 OR S28 OR S29 OR S30 OR S31 OR S32 OR S33 OR S34 OR S35 OR S36 OR S37 OR S38 OR S39 OR S40 OR S41 OR S42 OR S43 OR S44 OR S45 OR S46 OR S47 OR S48 OR S49 OR S50 OR S51 OR S52 OR S53 OR S54 OR S55 OR S56) |
| S84 | ("Project Head Start" or "Incredible Years" or "Sure Start") |
| S83 | (ZT "journal article*") or (ZT "article") |
| S82 | DE "Program Effectiveness" OR DE "Sustainability" |
| S81 | ("post intervention*" or postvention*) |
| S80 | (sessions N3 (“12 month*” or “twelve month*” or "365 days" or “13 month*” or “thirteen month*” or “14 month*” or “fourteen month*” or “15 month*” or “fifteen month*” or “16 month*” or “sixteen month*” or “17 month*” or “seventeen month*” or “18 month*” or “eighteen month*” or “19 month*” or “nineteen month*” or “20 month*” or “twenty month*” or “24 month*” or “twenty four month*” or “twentyfour month*” or “36 month*” or “thirtysix month*” or “thirty six month*” or “48 month*” or “fortyeight month*” or “forty eight month*” or “60 month*” or “sixty month*” or “1 year” or “one year” or “365 days” or “2 year*” or “two year*” or “3 year*” or “three year*” or “4 year*” or “four year*” or “5 year*” or “five year*” or “6 year*” or “six year*” or “7 year*” or “seven year*” or “8 year*” or “eight year*” or “9 year*” or “nine year*” or “10 year*” or “ten year*”)) |
| S79 | ((longterm or “long* term*”) N3 (asses* or effect* or impact* or outcome*)) |
| S78 | (sustain* N3 (effect* or impact)) |
| S77 | TI ("long* term" or maintenance or time or trajector* or posttreatment* or post-treatment*) |
| S76 | "year* later" |
| S75 | ("at" or after or later or "later than" or "longer than") N1 (“12 month*” or “twelve month*” or "365 days" or “13 month*” or “thirteen month*” or “14 month*” or “fourteen month*” or “15 month*” or “fifteen month*” or “16 month*” or “sixteen month*” or “17 month*” or “seventeen month*” or “18 month*” or “eighteen month*” or “19 month*” or “nineteen month*” or “20 month*” or “twenty month*” or “24 month*” or “twenty four month*” or “twentyfour month*” or “36 month*” or “thirtysix month*” or “thirty six month*” or “48 month*” or “fortyeight month*” or “forty eight month*” or “60 month*” or “sixty month*” or “1 year” or “one year” or “365 days” or “2 year*” or “two year*” or “3 year*” or “three year*” or “4 year*” or “four year*” or “5 year*” or “five year*” or “6 year*” or “six year*” or “7 year*” or “seven year*” or “8 year*” or “eight year*” or “9 year*” or “nine year*” or “10 year*” or “ten year*”)) |
| S74 | ((result or results or outcome or outcomes or measure* or effect or effects or “time point*” or timepoint or timepoints or treatment or treatments or posttreatment or posttreatments or therap* or baseline) N2 (“1 year” or “one year” or "365 days" or “2 year*” or “two year*” or “3 year*” or “three year*” or “4 year*” or “four year*” or “5 year*” or “five year*” or “6 year*” or “six year*” or “7 year*” or “seven year*” or “8 year*” or “eight year*” or “9 year*” or “nine year*” or “10 year*” or “ten year*”)) |
| S73 | ((result or results or outcome or outcomes or measure* or effect or effects or “time point*” or timepoint* or treatment or treatments or posttreatment or posttreatment* or therap* or baseline) N3 (“12 month*” or “twelve month*” or "365 days" or “13 month*” or “thirteen month*” or “14 month*” or “fourteen month*” or “15 month*” or “fifteen month*” or “16 month*” or “sixteen month*” or “17 month*” or “seventeen month*” or “18 month*” or “eighteen month*” or “19 month*” or “nineteen month*” or “20 month*” or “twenty month*” or “24 month*” or “twenty four month*” or “twentyfour month*” or “36 month*” or “thirtysix month*” or “thirty six month*” or “48 month*” or “fortyeight month*” or “forty eight month*” or “60 month*” or “sixty month*”)) |
| S72 | ((treatment N2 time) or (time N1 (effect* or segment?*))) |
| S71 | (followup* or "follow* up*") |
| S70 | DE "Outcomes of Education" OR DE "Longitudinal Studies" OR DE "Program Evaluation" |
| S69 | DE "Followup Studies" |
| S68 | ((control* N2 (trial or study or group)) and (placebo or waitlist* or wait* list* or ((treatment or care) N2 usual) or ("no intervention*" or "non intervention*" or non-intervention* or "without any intervention*"))) |
| S67 | ((single or double) N1 blind*) |
| S66 | (RCT or "at random" or (random* N3 (assign* or allocat* or control* or crossover or cross-over or design* or divide* or division or number))) |
| S65 | (randomi#ed or randomi#ation or randomi#ing) |
| S64 | (runaway or run-away or truant* or truancy) |
| S63 | (orphans or orphaned or orphanage* or (foster N1 (care or home*))) |
| S62 | ((youth or young) N2 (camp* or club* or detention or detainee* or group* or refugee* or immigrant*)) |
| S61 | SO (child* or adolescen* or pediatric* or paediatric*) |
| S60 | (child* or boy* or girl* or kids or juvenil* or minors or pediatric* or paediatric* or adolesc* or preadolesc* or pre-adolesc* or pubert* or pubescen* or prepube* or pre-pube* or teen* or (young* N1 (people or patient* or male* or female* or survivor* or offender* or minorit*)) or youth* or school* or classroom*) |
| S59 | (DE "High School Students" OR DE "Secondary School Students" OR DE "Middle School Students" OR DE "Junior High School Students" OR DE "Elementary School Students") OR (TI school* OR TI classroom*) |
| S58 | DE "Adolescents" OR DE "Early Adolescents" OR DE "Late Adolescents" OR DE "Youth" |
| S57 | DE "Children" OR DE "Migrant Children" OR DE "Latchkey Children" OR DE "Foster Care" OR DE "Minority Group Children" OR DE "Disadvantaged Youth" OR DE "Child Neglect" |
| S56 | (("high risk" or "at risk") N3 (population* or communit*)) |
| S55 | ( ((accident or emergency) N2 (department or ward)) ) AND ( (drinking or alcohol or drugs or crime or violence) ) AND brief |
| S54 | (((risk or risky) N2 (youth* or adolesc*)) or "risk taking" or "risk* behavi*") |
| S53 | TI poverty or disadvantaged or neglect* |
| S52 | ("disruptive behavi*" or "problem behavi*" or violence or violent or aggression or "aggressive behavi*" or conflict or conflicts or delinquen* or offend* or offence or convict* or criminal or recidivis* or probation or court-adjudicated) |
| S51 | DE "Refugees" OR DE "war" |
| S50 | DE "Civil Disobedience" OR DE "Social Discrimination" |
| S49 | DE "Poverty" OR DE "Disadvantaged Environment" OR DE "Economically Disadvantaged" OR DE "Homeless People" OR DE "Poverty Areas" OR DE "Poverty Programs" OR DE "Slums" |
| S48 | DE "At Risk Persons" OR DE "At Risk Students" |
| S47 | DE "Antisocial Behavior" OR DE "Aggression" OR DE "Bullying" OR DE "Child Abuse" OR DE "Child Neglect" OR DE "Crime" OR DE "Homicide" OR DE "Sexual Abuse" OR DE "Sexual Harassment" OR DE "Terrorism" OR DE "Vandalism" OR DE "Violence" |
| S46 | DE "Crime" OR DE "Delinquency" OR DE "Correctional Rehabilitation" OR DE "Criminals" OR DE "Family Violence" OR DE "Homicide" OR DE "Juvenile Courts" OR DE "Rape" OR DE "Terrorism" OR DE "Vandalism" |
| S45 | DE "Social Problems" OR DE "Conflict" |
| S44 | (divorce* or bereav* or grief or grieving) |
| S43 | DE "Family Problems" OR DE "Divorce" OR DE "Family Violence" OR DE "Runaways" |
| S42 | DE "Grief" |
| S41 | ("legal high*" or ((recreational or party or illicit*) N2 (drug* or substance*))) |
| S40 | ((addict* or dependen* or abuse or abuser or misuse*) N2 (aerosol* or alcohol* or amphetamin* or cannabi* or cocaine* or codeine or ecstasy or gases or GHB or heroin* or LSD or marihuana* or marijuana* or MDMA or methadone or methamphetamin* or morphine* or narcotic* or opiate* or opiod* or opium or psilocybin or psychedelic* or psychoactive* or psychostimulant* or solvent* or steroid* or stimulant*)) |
| S39 | ((alcohol or drug* or substance) N2 (abus* or misus* or use or user* or depend* or disorder)) |
| S38 | TI addict* |
| S37 | DE "Substance Abuse" OR DE "Alcohol Abuse" OR DE "Drug Abuse" OR DE "Addictive Behavior" |
| S36 | (asperger* or autis* or ((kanners or rett or pervasive development) N2 (child* or disorder* or syndrom* or symptom*)) or "childhood schizophrenia") |
| S35 | DE "Pervasive Developmental Disorders" OR DE "Asperger Syndrome" OR DE "Autism" |
| S34 | "personality disorder*" |
| S33 | DE "Personality Problems" |
| S32 | ((conduct or behavi* or personalit*) N2 (aggressi* or agressi* or antisocial or anti-social or dyssocial or defian* or deliquen* or disturb* or disrupt* or internalizing or internalising or externalizing or externalising or problem*)) |
| S31 | ((conduct or behavi* or antisocial or anti-social or dyssocial or emotional* or internalizing or internalising or externalizing or externalising) N2 (disorder* or problem* or difficult* or disturb* or psychopath*)) |
| S30 | (oppositional N3 (defian* or disorder*)) |
| S29 | DE "Behavior Disorders" OR DE "Behavior Problems" |
| S28 | ADHD or "attention defici*" or "minimal brain dysfunction" |
| S27 | DE "Attention Deficit Hyperactivity Disorder" OR DE "Attention Deficit Disorders" OR DE "Hyperactivity" |
| S26 | (somatoform or somatization or somatisation or "medical* unexplained" or MUPS or "body dysmorphi*") |
| S25 | "self injur*" or "self mutilat*" or suicide* or suicidal or parasuicid* or para-suicid* |
| S24 | DE "Suicide" |
| S23 | ( gambling or gambler* or trichotillomani* or firesetting or "fire setting" ) OR ( ((addicti* or impuls* or compulsi*) N2 (behavi* or disorder*)) ) |
| S22 | DE "Self Destructive Behavior" |
| S21 | ((psychologic* or school or social) N2 (adapt* or adjust*)) |
| S20 | ((mental* or psychologic*) N2 (health or well*)) |
| S19 | TI well-being OR TI wellbeing |
| S18 | well-being OR wellbeing |
| S17 | DE "Adjustment (to Environment)" OR DE "Social Adjustment" |
| S16 | ((selective or elective) N2 mutism) |
| S15 | (school N2 (refusal or dropout or drop-out)) |
| S14 | (emotional N2 (debrief* or stress* or trauma*)) |
| S13 | (psychological N2 (debrief* or stress* or trauma*)) |
| S12 | ((psychological or emotional) N2 (debrief* or stress* or trauma*)) |
| S11 | ((acute or chronic) N2 stress*) |
| S10 | ("anxiety disorder*" or "social* anxiety" or phobi* or agoraphobi* or ADNOS or "health anxiety" or hypochondri* or obsess* or compulsi* or panic or PTSD or "post traumatic stress" or "posttraumatic stress" or "stress disorder*" or neurosis or neuroses or neurotic) |
| S9 | TI anxiety |
| S8 | ((child* or adolesc* or teen* or youth or general*) N2 anxi*) |
| S7 | DE "Anxiety" |
| S6 | DE "Anxiety Disorders" |
| S5 | DE "Separation Anxiety" OR DE "Fear" OR DE "Posttraumatic Stress Disorder" OR DE "School Phobia" |
| S4 | mood* or depress* or dysthymi* or "affective disorder*" or "affective symptom*" |
| S3 | DE "Depression (Psychology)" |
| S2 | ("eating disorder*" or ` (eat* N3 mood*) or EDNOS or anorexi* or orthorexi* or bulimi* or diabulimi* or (bing* N3 eat*) or (bing N3 purg*)) |
| S1 | DE "Eating Disorders" |
