# Supplementary material for: Long-term outcomes of psychological interventions on children and young people’s mental health: A systematic review and meta-analysis
Source: PLoS One. 2020 Nov 16;15(11):e0236525. doi: 10.1371/journal.pone.0236525 (PMC7668611; doi:10.1371/journal.pone.0236525)
Supplement: S2 File — (DOCX) [file pone.0236525.s003.docx]

**S2 Fig: Extraction and data analysis guidelines**

**Scale Selection**

Select the most disorder specific outcome. For example, for anxiety if a study reports an anxiety specific measure and a depression measure, extract both measures but use the anxiety measure for anxiety analysis in CMA. Do not enter depression in CMA software for the outcome anxiety.

If there is not a disorder specific measure, select the best measure for that mental health condition. For example, for anxiety if the participant sample is a group of participants with OCD then we would use a specific OCD scale if available, however if some participants have OCD and others do not, this is a combined sample scale therefore an OCD scale would not be appropriate.

**Rater Selection**

**Conduct disorder = objective rater:** If a study reports both measures of self-report and objective rater (clinician/ independent rater/ parent/teacher), use the *objective rater over the self-report.* **In this case, select the best observer in the context of the study design.** If the study only includes self-report please include the self-report data.

**Anxiety, depression, PTSD = self-report:** If a study reports both measures of self-report and objective rater (clinician/ independent rater/ parent/teacher), use the *self-report data over the objective report.* If the study only includes clinician/ independent rater/parent/teacher report please include the independent report data (selecting most appropriate, as above).

**Substance misuse:** (1) objective measurement, for example urine, (2) self-report (3) clinician/independent (4) parent.

**Eating disorder:** (1) objective measurement, for example weight (anorexia/bulimia), (2) self-report (3) clinician/independent and (4) parent.

**School based interventions:** if selecting an independent observer, select teacher ratings if available.

A full hierarchy of preferred outcome measures is available from the corresponding author on request.
