# Supplementary material for: Long-term outcomes of psychological interventions on children and young people’s mental health: A systematic review and meta-analysis
Source: PLoS One. 2020 Nov 16;15(11):e0236525. doi: 10.1371/journal.pone.0236525 (PMC7668611; doi:10.1371/journal.pone.0236525)
Supplement: S3 File — (PDF) [file pone.0236525.s004.pdf]

	Study name	Comparison	Time point	Std diff in means	Standard error	Sample size	Effect direction	Std diff in means
1	Arnarson 2011	Depression Prevention Program v Assessment Only	Baseline-12	1.030	0.430	113	Positive	1.030
2	Augimeri 2007	SNAP v Recreational	Baseline-12	0.930	0.430	24	Positive	0.930
3	August 2004	CORE + COREFLEX v Control	Baseline-12	0.082	0.117	327	Positive	0.082
4	August 2004	CORE + COREFLEX v Control	Baseline-End	0.118	0.117	327	Positive	0.118
5	Barrett 1996	CBT + FAM v CBT	Baseline-12	-0.151	0.276	53	Negative	-0.151
6	Barrett 1996	CBT + FAM v CBT	Baseline-End	0.276	0.276	53	Positive	0.276
7	Barrett 1998	Group CBT + FAM v Group CBT	Baseline-12	1.887	0.436	31	Positive	1.887
8	Barrett 1998	Group CBT + FAM v Group CBT	Baseline-End	0.756	0.357	34	Positive	0.756
9	Barrett 2005	Individual CBT v Group CBT	Baseline-12	0.179	0.290	48	Positive	0.179
10	Barrett 2005	Individual CBT v Group CBT	Baseline-End	0.457	0.286	51	Positive	0.457
11	Barrington 2005	CBT v TAU	Baseline-12	-0.061	0.291	48	Negative	-0.061
12	Barrington 2005	CBT v TAU	Baseline-End	0.020	0.291	48	Positive	0.020
13	Bayer 2018	Cool little kids intervention v TAU	Baseline-12	0.327	0.086	545	Positive	0.327
14	Beardslee 2013	Cognitive-behavioural prevention program v Usual	Baseline-12	0.242	0.126	316	Positive	0.242
15	Bernal 1980	Parent Trainng v Parent Counselling	Baseline-12	0.950	0.360	34	Positive	0.950
16	Bernstein 2008	CBT v Control	Baseline-12	0.035	0.347	39	Positive	0.035
17	Bernstein 2008	CBT v Control	Baseline-End	-0.148	0.270	56	Negative	-0.148
18	Bjorseth 2016	PCIT v TAU	Baseline-12	0.562	0.253	65	Positive	0.562
19	Burke 2015	SNAP v Standard Beh. Service	Baseline-12	0.310	0.140	211	Positive	0.310
20	Butler 2011	MST v YOT	Baseline-12	0.461	0.202	101	Positive	0.461
21	Catwright-Hatton 2011	CBT Parenting Intervention v Waitlist control	Baseline-12	0.433	0.247	67	Positive	0.433
22	Catwright-Hatton 2011	CBT Parenting Intervention v Waitlist control	Baseline-End	0.827	0.255	67	Positive	0.827
23	Cavell 2000	Primetime v TAU	Baseline-12	-0.187	0.266	57	Negative	-0.187
24	Cavell 2000	Primetime v TAU	Baseline-End	0.247	0.259	60	Positive	0.247
25	Clark 1994	FIAP vs standard care	Baseline-12	0.450	0.200	109	Positive	0.450
26	Clark 2010	SUCCESS vs Control	Baseline-12	-0.022	0.049	1730	Negative	-0.022
27	Clark 2010	SUCCESS vs Control	Baseline-End	0.065	0.049	1730	Positive	0.065
28	Clarke 1995	Group CBT v TAU	Baseline-12	0.334	0.192	110	Positive	0.334
29	Clarke 1995	Group CBT v TAU	Baseline-End	0.828	0.199	110	Positive	0.828
30	Clarke 2001	Group cognitive therapy vs usual-care	Baseline-12	0.976	0.218	94	Positive	0.976
31	Clarke 2001	Group cognitive therapy vs usual-care	Baseline-End	0.872	0.216	94	Positive	0.872
32	Clarke 2002	Group CBT v TAU	Baseline-12	0.213	0.214	88	Positive	0.213
33	Clarke 2002	Group CBT v TAU	Baseline-End	-0.114	0.214	88	Negative	-0.114
34	Clarke 2016	TAU plus brief CBT v TAU	Baseline-12	0.115	0.137	212	Positive	0.115
35	Clarke 2016	TAU plus brief CBT v TAU	Baseline-End	0.666	0.141	212	Positive	0.666
36	Cobham 1998	CBT + Parent Anxiety Manafement v CBT	Baseline-12	0.397	0.454	20	Positive	0.397
37	Cobham 1998	CBT + Parent Anxiety Manafement v CBT	Baseline-End	0.752	0.465	20	Positive	0.752
38	Cohen 2005	Trauma Focused CBT v NST	Baseline-12	0.583	0.226	82	Positive	0.583
39	Cohen 2005	Trauma Focused CBT v NST	Baseline-End	0.249	0.222	82	Positive	0.249

	Std Err	Hedges's g	Std Err	ID	Setting	EOT	Agent	Severity	Contrast	Intensity	Disorder_code1	Disorder_Code2	Disorder_code3	Age	Manual
1	0.430	1.023	0.427	225	2	1	0	1	2	1	Blank	Blank	2	1	0
2	0.430	0.899	0.416	214	1	1	0	1	1	1	Blank	Blank	1	0	0
3	0.117	0.082	0.117	1	1	0	1	1	3	2	Conduct	1.	1	0	0
4	0.117	0.118	0.117	2	1	0	1	1	3	2	Conduct	1.	1	0	0
5	0.276	-0.148	0.272	3	0	0	0	1	0	1	Anxiety	4.	0	0	0
6	0.276	0.272	0.272	4	0	0	0	1	0	1	Anxiety	4.	0	0	0
7	0.436	1.840	0.425	5	0	0	0	1	0	1	Anxiety	4.	0	0	0
8	0.357	0.739	0.349	6	0	0	0	1	0	1	Anxiety	4.	0	0	0
9	0.290	0.176	0.286	7	0	0	0	1	0	1	Anxiety	4.	0	0	0
10	0.286	0.450	0.282	8	0	0	0	1	0	1	Anxiety	4.	0	0	0
11	0.291	-0.060	0.286	9	0	0	1	1	2	1	Anxiety	4.	0	0	0
12	0.291	0.020	0.286	10	0	0	1	1	2	1	Anxiety	4.	0	0	0
13	0.086	0.327	0.086		1	1	0	0	2	0	Anxiety	4.	0	0	0
14	0.126	0.241	0.126	226	0	1	0	1	2	1	Blank	Blank	2	1	0
15	0.360	0.928	0.352	217	0	1	0	1	0	0	Blank	Blank	1	0	0
16	0.347	0.034	0.340	11	2	0	0	1	3	1	Anxiety	4.	0	0	0
17	0.270	-0.146	0.267	12	2	0	0	1	3	1	Anxiety	4.	0	0	0
18	0.253	0.555	0.250	212	0	1	1	1	2	2	Conduct	1.	1	1	0
19	0.140	0.309	0.139	215	0	1	1	1	1	1	Blank	Blank	1	0	0
20	0.202	0.457	0.200	215	1	1	0	1	2	2	Conduct	1.	1	1	0
21	0.247	0.428	0.244	13	0	0	0	1	3	0	Anxiety	4.	0	0	0
22	0.255	0.818	0.252	14	0	0	0	1	3	0	Anxiety	4.	0	0	0
23	0.266	-0.185	0.262	15	2	0	1	1	2	2	Conduct	1.	1	0	0
24	0.259	0.244	0.256	16	2	0	1	1	2	2	Conduct	1.	1	0	0
25	0.200	0.447	0.199	218	1	1	0	0	2	2	Blank	Blank	1	0	1
26	0.049	-0.022	0.048	17	2	0	0	0	2	0	Substance	2.	5	1	0
27	0.049	0.065	0.048	18	2	0	0	0	2	0	Substance	2.	5	1	0
28	0.192	0.332	0.191	19	2	0	0	0	1	1	Depression	3.	2	1	0
29	0.199	0.822	0.198	20	2	0	0	0	1	1	Depression	3.	2	1	0
30	0.218	0.968	0.217	21	0	0	0	1	2	1	Depression	3.	2	1	0
31	0.216	0.865	0.214	22	0	0	0	1	2	1	Depression	3.	2	1	0
32	0.214	0.211	0.212	23	0	0	0	1	3	1	Depression	3.	2	1	0
33	0.214	-0.113	0.212	24	0	0	0	1	3	1	Depression	3.	2	1	0
34	0.137	0.115	0.137	25	0	0	0	1	2	0	Depression	3.	2	1	0
35	0.141	0.664	0.141	26	0	0	0	1	2	0	Depression	3.	2	1	0
36	0.454	0.382	0.436	27	0	0	0	1	0	1	Anxiety	4.	0	0	0
37	0.465	0.722	0.446	28	0	0	0	1	0	1	Anxiety	4.	0	0	0
38	0.226	0.578	0.223	29	0	0	0	1	0	1	PTSD	6. PTSD	4	0	0
39	0.222	0.247	0.220	30	0	0	0	1	0	1	PTSD	6. PTSD	4	0	0

	Nationality	Fidelity	Modality	Date	Date_Cat	Prevention	Format	ModalityBH vNBH	AF	AG	AH	AI	AJ	AK	AL
1	1	0	2	2011	2	Blank	0	Blank							
2	0	1	7	2007	1	Blank	0	Blank							
3	0	1	7	2004	1	1	0	0							
4	0	1	7	2004	1	1	0	0							
5	1	1	7	1996	0	0	1	0							
6	1	1	7	1996	0	0	1	0							
7	1	1	7	1998	0	0	0	0							
8	1	1	7	1998	0	0	0	0							
9	1	1	3	2005	1	0	1	0							
10	1	1	3	2005	1	0	1	0							
11	1	1	7	2005	1	0	0	0							
12	1	1	7	2005	1	0	0	0							
13	1	0	4	2018	2	Blank	0	1							
14	0	1	2	2013	2	Blank	0	Blank							
15	0	1	4	1980	0	Blank	1	Blank							
16	0	0	2	2008	1	0	0	1							
17	0	0	2	2008	1	0	0	1							
18	1	1	4	2016	2	0	1	1							
19	0	0	7	2015	2	Blank	0	Blank							
20	1	1	4	2011	2	0	1	1							
21	1	1	4	2011	2	0	0	1							
22	1	1	4	2011	2	0	0	1							
23	0	0	7	2000	1	1	0	0							
24	0	0	7	2000	1	1	0	0							
25	0	0	8	1994	0	Blank	1	Blank							
26	0	0	7	2010	2	1	0	0							
27	0	0	7	2010	2	1	0	0							
28	0	1	2	1995	0	1	0	1							
29	0	1	2	1995	0	1	0	1							
30	0	1	2	2001	1	1	0	1							
31	0	1	2	2001	1	1	0	1							
32	0	1	2	2002	1	0	0	1							
33	0	1	2	2002	1	0	0	1							
34	0	1	1	2016	2	0	1	1							
35	0	1	1	2016	2	0	1	1							
36	1	1	7	2010	2	0	0	0							
37	1	1	7	2010	2	0	0	0							
38	0	1	1	2005	1	0	1	1							
39	0	1	1	2005	1	0	1	1							

	AM	AN	AO	AP	AQ	AR	AS
1							
2							
3							
4							
5							
6							
7							
8							
9							
10							
11							
12							
13							
14							
15							
16							
17							
18							
19							
20							
21							
22							
23							
24							
25							
26							
27							
28							
29							
30							
31							
32							
33							
34							
35							
36							
37							
38							
39							

	Study name	Comparison	Time point	Std diff in means	Standard error	Sample size	Effect direction	Std diff in means
40	Conrod 2010	Preventure vs Control	Baseline-12	0.261	0.077	691	Positive	0.261
41	Conrod 2011	Preventure vs Control	Baseline-12	0.141	0.108	347	Positive	0.141
42	Creswell 2015	CCBT + MCBT v CCBT	Baseline-12	0.222	0.187	211	Positive	0.222
43	Creswell 2015	CCBT + MCI v CCBT	Baseline-12	0.124	0.185	211	Positive	0.124
44	Cunningham 2012a	Computer v Control	Baseline-12	0.060	0.110	403	Positive	0.060
45	Cunningham 2012a	Therapist Group v Control	Baseline-12	0.286	0.111	406	Positive	0.286
46	Cunningham 2012b	Computer v Control	Baseline-12	0.148	0.118	403	Positive	0.148
47	Cunningham 2012b	Therapist Group v Control	Baseline-12	-0.062	0.114	406	Negative	-0.062
48	Dakof 2015	Multidimensional Family Treatment Vs Adolescent	Baseline-12	-0.242	0.249	66	Negative	-0.242
49	Dakof 2015	Multidimensional Family Treatment Vs Adolescent	Baseline-End	0.114	0.198	102	Positive	0.114
50	Damico 2018	CHAT brief motivational interviewing intervention v	Baseline-12	0.093	0.117	294	Positive	0.093
51	Deblinger 1999	Child only v control	Baseline-12	0.365	0.439	28	Positive	0.365
52	Deblinger 1999	Child only v control	Baseline-End	1.009	0.457	28	Positive	1.009
53	Deblinger 1999	Mother and child treatment v control	Baseline-12	1.356	0.480	26	Positive	1.356
54	Deblinger 1999	Mother and child treatment v control	Baseline-End	1.583	0.494	26	Positive	1.583
55	Deblinger 2006	Trauma focused CBT V Child focused therapy	Baseline-12	0.250	0.162	153	Positive	0.250
56	Deblinger 2006	Trauma focused CBT V Child focused therapy	Baseline-End	0.635	0.153	180	Positive	0.635
57	Dishion 1995	Parent teen focus v Self directed change (Control	Baseline-12	0.041	0.276	53	Positive	0.041
58	Dishion 1995	Parent teen focus v Self directed change (Control	Baseline-End	-0.258	0.280	52	Negative	-0.258
59	Duong 2016	PTA v ISP	Baseline-12	0.435	0.203	100	Positive	0.435
60	Duong 2016	PTA v ISP	Baseline-End	0.649	0.195	111	Positive	0.649
61	Estrada 2019	eHealth Familias Unidas v TAU	Baseline-12	-0.124	0.132	230	Negative	-0.124
62	Estrada 2019	eHealth Familias Unidas v TAU	Baseline-End	-0.054	0.132	230	Negative	-0.054
63	Flannery-Shroeder 2005	Individual CBT v Group CBT	Baseline-12	0.647	0.461	20	Positive	0.647
64	Flannery-Shroeder 2005	Individual CBT v Group CBT	Baseline-End	-0.038	0.400	25	Negative	-0.038
65	Foa 2013	Polonged Exposure v Supportive Counselling	Baseline-12	0.913	0.269	61	Positive	0.913
66	Foa 2013	Polonged Exposure v Supportive Counselling	Baseline-End	1.103	0.275	61	Positive	1.103
67	Forgatch 1999	Parent training groups v Control	Baseline-12	-0.049	0.166	157	Negative	-0.049
68	Garcia-Lopez 2014	Therapy with Parental Involvement v	Baseline-12	1.044	0.303	52	Positive	1.044
69	Garcia-Lopez 2014	Therapy with Parental Involvement v	Baseline-End	1.452	0.319	52	Positive	1.452
70	Ghaderi 2018	iComet internet parent training v family check up	Baseline-12	-0.303	0.133	231	Negative	-0.303
71	Ghaderi 2018	iComet internet parent training v family check up	Baseline-End	-0.415	0.133	231	Negative	-0.415
72	Godley 2010	Chestnut Bloomington Output with Assertive	Baseline-12	0.118	0.208	161	Positive	0.118
73	Godley 2014	Assertive Continuing Care (CC) + Continguency	Baseline-12	0.155	0.158	161	Positive	0.155
74	Goodyer 2017	CBT v Control	Baseline-12	0.123	0.139	232	Positive	0.123
75	Goodyer 2017	CBT v Control	Baseline-End	0.613	0.142	232	Positive	0.613
76	Goodyer 2017	Short Term psychoanalytic therapy v Control	Baseline-12	0.096	0.139	234	Positive	0.096
77	Goodyer 2017	Short Term psychoanalytic therapy v Control	Baseline-End	0.294	0.139	234	Positive	0.294
78	Goossens 2016	Preventure v Control	Baseline-12	0.111	0.087	530	Positive	0.111

	Std Err	Hedges's g	Std Err	ID	Setting	EOT	Agent	Severity	Contrast	Intensity	Disorder_code1	Disorder_Code2	Disorder_code3	Age	Manual
40	0.077	0.261	0.077	216	2	1	0	0	3	0	Substance	2.	5	1	0
41	0.108	0.141	0.108	217	2	1	0	0	2	0	Substance	2.	5	1	0
42	0.187	0.221	0.186	232	1	1	0	1	0	1	Blank	Blank	0	0	0
43	0.185	0.124	0.184	231	1	1	0	1	0	1	Blank	Blank	0	0	0
44	0.110	0.060	0.110	223	0	1	1	0	1	0	Blank	Blank	1	1	0
45	0.111	0.285	0.111	222	0	1	0	0	1	0	Blank	Blank	1	1	0
46	0.118	0.148	0.118	221	0	1	1	0	1	0	Blank	Blank	5	1	0
47	0.114	-0.062	0.114	220	0	1	0	0	1	0	Blank	Blank	5	1	0
48	0.249	-0.239	0.246	31	0	0	0	1	0	2	Substance	2.	5	1	0
49	0.198	0.113	0.197	32	0	0	0	1	0	2	Substance	2.	5	1	0
50	0.117	0.093	0.117		0	1	1	1	2	0	Substance	2.	5	1	1
51	0.439	0.354	0.427	33	0	0	0	1	2	1	PTSD	6. PTSD	4	0	0
52	0.457	0.980	0.444	34	0	0	0	1	2	1	PTSD	6. PTSD	4	0	0
53	0.480	1.314	0.466	35	0	0	0	1	2	1	PTSD	6. PTSD	4	0	0
54	0.494	1.535	0.479	36	0	0	0	1	2	1	PTSD	6. PTSD	4	0	0
55	0.162	0.248	0.162	37	0	0	0	1	0	1	PTSD	6. PTSD	4	0	0
56	0.153	0.633	0.152	38	0	0	0	1	0	1	PTSD	6. PTSD	4	0	0
57	0.276	0.040	0.272	40	1	0	1	0	0	2	Conduct	1.	1	1	0
58	0.280	-0.254	0.276	39	1	0	1	0	0	2	Conduct	1.	1	1	0
59	0.203	0.431	0.201	41	2	0	1	1	0	1	Depression	3.	2	1	0
60	0.195	0.645	0.194	42	2	0	1	1	0	1	Depression	3.	2	1	0
61	0.132	-0.123	0.132		1	0	1	0	2	1	Substance	2.	5	1	1
62	0.132	-0.054	0.131		1	0	1	0	2	1	Substance	2.	5	1	1
63	0.461	0.621	0.443	43	0	0	1	1	0	1	Anxiety	4.	0	0	0
64	0.400	-0.037	0.388	44	0	0	1	1	0	1	Anxiety	4.	0	0	0
65	0.269	0.901	0.266	45	0	0	0	1	0	1	PTSD	6. PTSD	4	1	0
66	0.275	1.089	0.271	46	0	0	0	1	0	1	PTSD	6. PTSD	4	1	0
67	0.166	-0.049	0.165	218	1	1	1	0	3	1	Conduct	1.	1	0	0
68	0.303	1.028	0.298	47	2	0	1	1	0	1	Anxiety	4.	0	1	0
69	0.319	1.430	0.314	48	2	0	1	1	0	1	Anxiety	4.	0	1	0
70	0.133	-0.302	0.132		1	0	0	1	0	0	Conduct	1.	1	0	0
71	0.133	-0.413	0.133		1	0	0	1	0	0	Conduct	1.	1	0	0
72	0.208	0.117	0.207	227	1	1	1	1	0	2	Blank	Blank	5	1	0
73	0.158	0.154	0.157	219	1	1	0	1	2	0	Substance	2.	5	1	0
74	0.139	0.122	0.139	49	0	0	0	1	2	2	Depression	3.	2	1	0
75	0.142	0.611	0.141	50	0	0	0	1	2	2	Depression	3.	2	1	0
76	0.139	0.095	0.138	52	0	0	0	1	2	2	Depression	3.	2	1	0
77	0.139	0.293	0.139	51	0	0	0	1	2	2	Depression	3.	2	1	0
78	0.087	0.111	0.087	220	2	1	0	0	3	1	Conduct	1.	1	1	0

	Nationality	Fidelity	Modality	Date	Date_Cat	Prevention	Format	ModalityBH vNBH	AF	AG	AH	AI	AJ	AK	AL
40	1	0	8	2010	2	1	0	0							
41	1	0	8	2011	2	1	0	0							
42	1	1	7	2015	2	Blank	0	Blank							
43	1	1	7	2015	2	Blank	0	Blank							
44	0	0	8	2012	2	Blank	1	Blank							
45	0	0	8	2012	2	Blank	1	Blank							
46	0	1	8	2012	2	Blank	1	Blank							
47	0	0	8	2012	2	Blank	1	Blank							
48	0	1	3	2015	2	1	1	0							
49	1	1	3	2015	2	0	1	0							
50	0	1	8	2018	2	Blank	1	0							
51	0	0	1	1999	0	0	1	1							
52	0	0	1	1999	0	0	1	1							
53	0	0	3	1999	0	0	1	0							
54	0	0	3	1999	0	0	1	0							
55	0	1	7	2006	1	0	1	0							
56	0	1	7	2006	1	0	1	0							
57	0	0	3	1995	0	1	0	0							
58	0	0	3	1995	0	1	0	0							
59	0	1	2	2016	2	1	0	1							
60	0	1	2	2016	2	1	0	1							
61	0	1	3	2019	2	Blank	0	0							
62	0	1	3	2019	2	Blank	0	0							
63	0	0	1	2005	1	0	1	1							
64	0	0	1	2005	1	0	1	1							
65	0	1	1	2013	2	0	1	1							
66	0	1	1	2013	2	0	1	1							
67	0	1	4	1999	0	1	0	1							
68	1	1	7	2014	2	0	0	0							
69	1	1	7	2014	2	0	0	0							
70	1	1	4	2018	2	Blank	1	1							
71	1	1	4	2018	2	Blank	1	1							
72	0	1	7	2010	2	Blank	0	Blank							
73	0	0	1	2014	2	0	1	1							
74	1	1	1	2017	2	0	1	1							
75	1	1	1	2017	2	0	1	1							
76	1	1	6	2017	2	0	1	0							
77	1	1	6	2017	2	0	1	0							
78	1	0	8	2016	2	1	0	0							

	AM	AN	AO	AP	AQ	AR	AS
40							
41							
42							
43							
44							
45							
46							
47							
48							
49							
50							
51							
52							
53							
54							
55							
56							
57							
58							
59							
60							
61							
62							
63							
64							
65							
66							
67							
68							
69							
70							
71							
72							
73							
74							
75							
76							
77							
78							

	Study name	Comparison	Time point	Std diff in means	Standard error	Sample size	Effect direction	Std diff in means
79	Gowers 2007	Specialised out-patient v TAU	Baseline-12	-0.121	0.198	102	Negative	-0.121
80	Hagen 2011	PMTO v Regular Services	Baseline-12	0.504	0.192	112	Positive	0.504
81	Hagen 2011	PMTO v Regular Services	Baseline-End	0.382	0.191	112	Positive	0.382
82	Halldorsdottir 2016	Exposure treatment v educational support	Baseline-12	0.322	0.226	83	Positive	0.322
83	Halldorsdottir 2016	Exposure treatment v educational support	Baseline-End	1.075	0.239	83	Positive	1.075
84	Hautmann 2018	Behavioural v non beahvioural guided self help	Baseline-12	0.240	0.192	110	Positive	0.240
85	Hautmann 2018	Behavioural v non beahvioural guided self help	Baseline-End	0.401	0.193	110	Positive	0.401
86	Humayun 2017	Functional Family Therapy v TAU	Baseline-12	-0.069	0.193	111	Negative	-0.069
87	Hurlbert 2013	Head Start + Incredible Years v just Head start	Baseline-12	0.091	0.110	378	Positive	0.091
88	Hurlbert 2013	Head Start + Incredible Years v just Head start	Baseline-End	0.217	0.111	378	Positive	0.217
89	Jouriles 2009	Poject Support v Existing Services (TAU)	Baseline-12	0.630	0.300	66	Positive	0.630
90	Kazdin 1992	PSST+ Parent Management Training (PMT) v PMT	Baseline-12	1.501	0.335	47	Positive	1.501
91	Kazdin 1992	PSST+ Parent Management Training (PMT) v PMT	Baseline-End	0.553	0.292	50	Positive	0.553
92	Kendall 2008	FCBT v FESA (Control)	Baseline-12	3.149	0.352	81	Positive	3.149
93	Kendall 2008	FCBT v FESA (Control)	Baseline-End	5.695	0.508	81	Positive	5.695
94	Kendall 2008	ICBT v FESA (Control)	Baseline-12	2.290	0.305	80	Positive	2.290
95	Kendall 2008	ICBT v FESA (Control)	Baseline-End	4.729	0.438	80	Positive	4.729
96	Lammers 2015	Coping Skills Intervention v No intervention control	Baseline-12	0.140	0.124	322	Positive	0.140
97	Larsson 2009	Parent Training + Child Training v Parent Training only	Baseline-12	0.240	0.195	106	Positive	0.240
98	Larsson 2009	Parent Training + Child Training v Parent Training only	Baseline-End	0.198	0.195	106	Positive	0.198
99	Le Grange 2015	Family Treatment v CBT	Baseline-12	0.191	0.192	109	Positive	0.191
100	Le Grange 2015	Family Treatment v CBT	Baseline-End	0.123	0.192	109	Positive	0.123
101	Le Grange 2016	Parent Intervention v Family Treatment	Baseline-12	-0.180	0.195	106	Negative	-0.180
102	Le Grange 2016	Parent Intervention v Family Treatment	Baseline-End	-0.326	0.196	106	Negative	-0.326
103	Lee_2016/Bernstein_2005	CBT/ CBT + parental training v Control	Baseline-12	0.584	0.267	61	Positive	0.584
104	Lee_2016/Bernstein_2005	CBT/ CBT + parental training v Control	Baseline-End	-0.148	0.262	61	Negative	-0.148
105	Letourneau 2013	MST v TAU	Baseline-12	-0.018	0.180	124	Negative	-0.018
106	Lewinsohn 1990	Child + Parent CBT v Child CBT	Baseline-12	-0.005	0.372	29	Negative	-0.005
107	Lewinsohn 1990	Child + Parent CBT v Child CBT	Baseline-End	0.266	0.318	40	Positive	0.266
108	Liddle 2001	Multidimensional Family Treatment Vs Adolescent	Baseline-12	0.761	0.266	61	Positive	0.761
109	Liddle 2001	Multidimensional Family Treatment Vs Adolescent	Baseline-End	1.492	0.290	61	Positive	1.492
110	Liddle 2008	MDFT Vs CBT	Baseline-12	0.683	0.137	224	Positive	0.683
111	Liddle 2008	MDFT Vs CBT	Baseline-End	0.678	0.137	224	Positive	0.678
112	Lochman 2004	Child and parent intervention v control	Baseline-12	0.392	0.221	92	Positive	0.392
113	Lochman 2004	Child intervention v control	Baseline-12	-0.134	0.219	92	Negative	-0.134
114	Lochman 2014	Coping Power v Control	Baseline-12	0.547	0.196	126	Positive	0.547
115	Lochman 2014	Coping Power v Control	Baseline-End	0.271	0.182	141	Positive	0.271
116	Lochman 2015	Individual coping power v Group coping power	Baseline-12	0.592	0.121	285	Positive	0.592
117	Lochman 2015	Individual coping power v Group coping power	Baseline-End	-0.002	0.110	329	Negative	-0.002

	Std Err	Hedges's g	Std Err	ID	Setting	EOT	Agent	Severity	Contrast	Intensity	Disorder_code1	Disorder_Code2	Disorder_code3	Age	Manual
79	0.198	-0.120	0.197	221	0	1	0	1	2	2	Eating	5.	3	1	0
80	0.192	0.501	0.191	53	1	0	0	1	2	1	Conduct	1.	1	0	0
81	0.191	0.380	0.190	54	1	0	0	1	2	1	Conduct	1.	1	0	0
82	0.226	0.319	0.224	55	0	0	0	1	0	0	Anxiety	4.	0	0	0
83	0.239	1.065	0.237	56	0	0	0	1	0	0	Anxiety	4.	0	0	0
84	0.192	0.239	0.191		1	0	0	1	0	1	Conduct	1.	1	0	0
85	0.193	0.398	0.192		1	0	0	1	0	1	Conduct	1.	1	0	0
86	0.193	-0.069	0.191	222	0	1	0	1	2	1	Conduct	1.	1	1	1
87	0.110	0.090	0.110	58	1	0	1	0	2	0	Conduct	1.	1	0	0
88	0.111	0.217	0.110	59	1	0	1	0	2	0	Conduct	1.	1	0	0
89	0.300	0.623	0.297	216	1	1	0	0	2	1	Blank	Blank	1	0	0
90	0.335	1.477	0.330	60	0	0	0	1	0	2	Conduct	1.	1	0	0
91	0.292	0.544	0.287	57	0	0	0	1	0	2	Conduct	1.	1	0	0
92	0.352	3.119	0.349	61	0	0	0	1	1	1	Anxiety	4.	0	0	0
93	0.508	5.642	0.503	62	0	0	0	1	1	1	Anxiety	4.	0	0	0
94	0.305	2.268	0.302	63	0	0	0	1	1	1	Anxiety	4.	0	0	0
95	0.438	4.684	0.434	64	0	0	0	1	1	1	Anxiety	4.	0	0	0
96	0.124	0.139	0.124	228	2	1	0	0	3	0	Blank	Blank	5	1	0
97	0.195	0.238	0.194	70	0	0	0	1	0	2	Conduct	1.	1	0	0
98	0.195	0.196	0.193	69	0	0	0	1	0	2	Conduct	1.	1	0	0
99	0.192	0.190	0.191	65	0	0	0	1	0	1	Eating	5.	3	1	0
100	0.192	0.122	0.191	66	0	0	0	1	0	1	Eating	5.	3	1	0
101	0.195	-0.179	0.193	67	0	0	0	1	0	1	Eating	5.	3	1	0
102	0.196	-0.324	0.194	68	0	0	0	1	0	1	Eating	5.	3	1	0
103	0.267	0.577	0.264	71	2	0	0	1	3	0	Anxiety	4.	0	0	0
104	0.262	-0.146	0.259	73	2	0	0	1	3	0	Anxiety	4.	0	0	0
105	0.180	-0.018	0.179	223	1	1	0	1	2	2	Conduct	1.	1	1	0
106	0.372	-0.005	0.362	72	0	0	1	1	0	0	Depression	3.	2	1	0
107	0.318	0.260	0.312	74	0	0	1	1	0	0	Depression	3.	2	1	0
108	0.266	0.751	0.263	75	0	0	0	1	0	1	Substance	2.	5	1	0
109	0.290	1.473	0.287	76	1	0	0	1	0	1	Substance	2.	5	1	0
110	0.137	0.681	0.137	77	0	0	0	1	0	1	Substance	2.	5	1	0
111	0.137	0.676	0.137	78	0	0	0	1	0	1	Substance	2.	5	1	0
112	0.221	0.389	0.219	225	1	1	1	1	3	1	Conduct	1.	1	0	0
113	0.219	-0.133	0.217	224	1	1	1	1	3	2	Conduct	1.	1	0	0
114	0.196	0.544	0.195	79	2	0	1	0	2	0	Conduct	1.	1	0	0
115	0.182	0.269	0.181	80	2	0	1	0	2	0	Conduct	1.	1	0	0
116	0.121	0.590	0.121	81	2	0	1	0	0	2	Conduct	1.	1	0	0
117	0.110	-0.002	0.110	82	2	0	1	0	0	2	Conduct	1.	1	0	0

	Nationality	Fidelity	Modality	Date	Date_Cat	Prevention	Format	ModalityBH vNBH	AF	AG	AH	AI	AJ	AK	AL
79	1	1	7	2007	1	0	1	0							
80	1	1	4	2011	2	0	1	1							
81	1	1	4	2011	2	0	1	1							
82	1	0	1	2016	2	0	1	1							
83	1	0	1	2016	2	0	1	1							
84	1	0	4	2018	2	Blank	1	1							
85	1	0	4	2018	2	Blank	1	1							
86	1	0	3	2017	2	0	1	0							
87	0	1	4	2013	2	1	0	1							
88	0	1	4	2013	2	1	0	1							
89	0	0	4	2009	1	Blank	1	Blank							
90	0	0	7	1992	0	0	1	0							
91	0	0	7	1992	0	0	1	0							
92	0	1	3	2008	1	0	1	0							
93	0	1	3	2008	1	0	1	0							
94	0	1	1	2008	1	0	1	1							
95	0	1	1	2008	1	0	1	1							
96	1	0	8	2015	2	Blank	0	Blank							
97	1	0	7	2009	1	0	0	0							
98	1	0	7	2009	1	0	0	0							
99	0	0	3	2015	2	0	1	0							
100	0	0	3	2015	2	0	1	0							
101	1	0	4	2016	2	0	1	1							
102	1	0	4	2016	2	0	1	1							
103	0	0	2	2005	1	0	0	1							
104	0	0	2	2005	1	0	0	1							
105	0	1	4	2013	2	0	1	1							
106	0	1	7	1990	0	0	0	0							
107	0	1	7	1990	0	0	0	0							
108	0	0	3	2001	1	0	1	0							
109	0	0	3	2001	1	0	1	0							
110	0	1	3	2008	1	0	1	0							
111	0	1	3	2008	1	0	1	0							
112	0	0	1	2004	1	0	0	1							
113	0	0	7	2004	1	0	0	0							
114	0	0	2	2014	2	1	0	1							
115	0	0	2	2014	2	1	0	1							
116	0	0	1	2015	2	1	1	1							
117	0	0	1	2015	2	1	1	1							

	AM	AN	AO	AP	AQ	AR	AS
79							
80							
81							
82							
83							
84							
85							
86							
87							
88							
89							
90							
91							
92							
93							
94							
95							
96							
97							
98							
99							
100							
101							
102							
103							
104							
105							
106							
107							
108							
109							
110							
111							
112							
113							
114							
115							
116							
117							

	Study name	Comparison	Time point	Std diff in means	Standard error	Sample size	Effect direction	Std diff in means
118	Lock 2010	Family Therapy v Individual Therapy	Baseline-12	-0.039	0.208	93	Negative	-0.039
119	Lock 2010	Family Therapy v Individual Therapy	Baseline-End	-0.012	0.205	96	Negative	-0.012
120	Mahu 2015	Adventure vs Control	Baseline-12	0.438	0.068	900	Positive	0.438
121	Mannarino 2012/Deblinger 2011	CBT + Trauma Narrative v CBT no trauma narrative	Baseline-12	-0.150	0.265	57	Negative	-0.150
122	Mannassis 2010a	CBT v Control	Baseline-12	-0.147	0.167	145	Negative	-0.147
123	Mannassis 2010a	CBT v Control	Baseline-End	-0.129	0.166	145	Negative	-0.129
124	Mannassis 2010b	CBT v Control	Baseline-12	-0.241	0.167	145	Negative	-0.241
125	Mannassis 2010b	CBT v Control	Baseline-End	-0.517	0.169	145	Negative	-0.517
126	McGrath 2011a	Strongest Families v Usual Care	Baseline-12	0.420	0.270	80	Positive	0.420
127	McGrath 2011b	Strongest Families v Usual Care	Baseline-12	0.507	0.257	90	Positive	0.507
128	Newton 2016	Preventure vs Control	Baseline-12	0.714	0.113	344	Positive	0.714
129	Ogden 2006	MST v TAU	Baseline-12	0.413	0.239	75	Positive	0.413
130	Olivares 2014	Experienced therapist v inexperienced	Baseline-12	0.717	0.238	75	Positive	0.717
131	Olivares 2014	Experienced therapist v inexperienced	Baseline-End	0.474	0.234	75	Positive	0.474
132	Olivares-Olivares 2008	IAFS + Individual tratment v IAFS only	Baseline-12	1.106	0.353	37	Positive	1.106
133	Olivares-Olivares 2008	IAFS + Individual tratment v IAFS only	Baseline-End	1.333	0.364	37	Positive	1.333
134	Olthius 2018	Strongest families v usual care	Baseline-12	0.184	0.153	172	Positive	0.184
135	Olthius 2018	Strongest families v usual care	Baseline-End	0.554	0.155	172	Positive	0.554
136	O'Shea 2015	Individual Psychotherapy v Group	Baseline-12	0.405	0.324	39	Positive	0.405
137	O'Shea 2015	Individual Psychotherapy v Group	Baseline-End	-0.045	0.320	39	Negative	-0.045
138	Ost 2001	Exposure (parent) v Exposure (child only)	Baseline-12	0.316	0.260	60	Positive	0.316
139	Ost 2001	Exposure (parent) v Exposure (child only)	Baseline-End	0.190	0.259	60	Positive	0.190
140	Ost 2015	Exposure Therapy (ET) + Group SST + Parenting v	Baseline-12	0.029	0.278	52	Positive	0.029
141	Ost 2015	Exposure therapy and group social skills training +	Baseline-End	0.202	0.279	52	Positive	0.202
142	Pella 2017/ Ginsburg 2015	CAPS v Information monitoring control	Baseline-12	0.168	0.172	136	Positive	0.168
143	Pella 2017/ Ginsburg 2015	CAPS v Information monitoring control	Baseline-End	-0.029	0.172	136	Negative	-0.029
144	Poppelaars 2016	SPARX & OVK v Contol	Baseline-12	0.049	0.194	107	Positive	0.049
145	Poppelaars 2016	SPARX & OVK v Contol	Baseline-End	-0.001	0.194	107	Negative	-0.001
146	Rasing 2018a	'Een Sprong Vooruit' (A Leap Forward) v waitlist	Baseline-12	0.201	0.168	142	Positive	0.201
147	Rasing 2018a	'Een Sprong Vooruit' (A Leap Forward) v waitlist	Baseline-End	0.062	0.168	142	Positive	0.062
148	Rasing 2018b	'Een Sprong Vooruit' (A Leap Forward) v waitlist	Baseline-12	0.129	0.168	142	Positive	0.129
149	Rasing 2018b	'Een Sprong Vooruit' (A Leap Forward) v waitlist	Baseline-End	0.086	0.168	142	Positive	0.086
150	Robin 1995	BFST v EOIT	Baseline-12	1.866	0.538	20	Positive	1.866
151	Robin 1995	BFST v EOIT	Baseline-End	1.552	0.486	22	Positive	1.552
152	Robin 1999	BFST v EOIT	Baseline-12	1.957	0.406	36	Positive	1.957
153	Robin 1999	BFST v EOIT	Baseline-End	1.756	0.393	36	Positive	1.756
154	Rohde 2004a	CWD-A v Life Skills	Baseline-12	-1.098	0.230	87	Negative	-1.098
155	Rohde 2004a	CWD-A v Life Skills	Baseline-End	0.010	0.210	91	Positive	0.010
156	Rohde 2004b	CWD-A v Life Skills	Baseline-12	-0.155	0.215	87	Negative	-0.155

	Std Err	Hedges's g	Std Err	ID	Setting	EOT	Agent	Severity	Contrast	Intensity	Disorder_code1	Disorder_Code2	Disorder_code3	Age	Manual
118	0.208	-0.038	0.206	83	0	0	0	1	0	2	Eating	5.	3	1	0
119	0.205	-0.012	0.203	84	0	0	0	1	0	2	Eating	5.	3	1	0
120	0.068	0.438	0.068	226	2	1	0	1	2	0	Substance	2.	5	1	0
121	0.265	-0.148	0.262	233	0	1	0	1	0	1	Blank	Blank	4	0	0
122	0.167	-0.146	0.166	85	2	0	0	1	1	1	Anxiety	4.	0	0	0
123	0.166	-0.128	0.166	86	2	0	0	1	1	1	Anxiety	4.	0	0	0
124	0.167	-0.240	0.166	87	2	0	0	0	2	1	Depression	3.	2	0	0
125	0.169	-0.514	0.168	88	2	0	0	0	2	1	Depression	3.	2	0	0
126	0.270	0.416	0.267	224	1	1	1	1	2	1	Conduct	Blank	1	0	0
127	0.257	0.503	0.254	235	1	1	1	1	2	1	Anxiety	Blank	0	0	0
128	0.113	0.713	0.113	227	2	1	0	0	1	0	Substance	2.	5	1	0
129	0.239	0.409	0.237	228	1	1	0	1	2	2	Conduct	1.	1	1	0
130	0.238	0.709	0.236	89	2	0	0	1	0	1	Anxiety	4.	0	1	0
131	0.234	0.469	0.232	90	2	0	0	1	0	1	Anxiety	4.	0	1	0
132	0.353	1.083	0.346	91	2	0	0	1	0	2	Anxiety	4.	0	1	0
133	0.364	1.305	0.356	92	2	0	0	1	0	2	Anxiety	4.	0	1	0
134	0.153	0.184	0.152		1	0	Blank	1	2	1	Conduct	1.	1	0	1
135	0.155	0.552	0.155		1	0	Blank	1	2	1	Conduct	1.	1	0	1
136	0.324	0.397	0.317	93	2	0	0	1	0	1	Depression	3.	2	1	0
137	0.320	-0.044	0.314	94	2	0	0	1	0	1	Depression	3.	2	1	0
138	0.260	0.312	0.256	95	1	0	0	1	0	0	Anxiety	4.	0	0	0
139	0.259	0.188	0.255	96	1	0	0	1	0	0	Anxiety	4.	0	0	0
140	0.278	0.029	0.274	97	0	0	0	1	0	2	Anxiety	4.	0	0	0
141	0.279	0.199	0.275	98	0	0	0	1	0	2	Anxiety	4.	0	0	0
142	0.172	0.167	0.171	99	0	0	0	0	1	0	Anxiety	4.	0	0	0
143	0.172	-0.029	0.171	100	0	0	0	0	1	0	Anxiety	4.	0	0	0
144	0.194	0.048	0.192	101	1	0	0	1	3	0	Depression	3.	2	1	0
145	0.194	-0.001	0.192	102	1	0	0	1	3	0	Depression	3.	2	1	0
146	0.168	0.200	0.167		Blank	0	0	1	3	0	Depression	3.	2	1	1
147	0.168	0.061	0.167		Blank	0	0	1	3	0	Depression	3.	2	1	1
148	0.168	0.128	0.167		Blank	0	0	1	3	0	Anxiety	4.	0	1	1
149	0.168	0.086	0.167		Blank	0	0	1	3	0	Anxiety	4.	0	1	1
150	0.538	1.791	0.516	103	0	0	0	1	0	2	Eating	5.	3	1	0
151	0.486	1.496	0.469	104	0	0	0	1	0	2	Eating	5.	3	1	0
152	0.406	1.915	0.397	105	0	0	0	1	0	2	Eating	5.	3	1	0
153	0.393	1.718	0.384	106	0	0	0	1	0	2	Eating	5.	3	1	0
154	0.230	-1.089	0.228	107	0	0	1	1	0	1	Conduct	1.	1	1	0
155	0.210	0.010	0.208	108	0	0	1	1	0	1	Conduct	1.	1	1	0
156	0.215	-0.153	0.213	109	1	0	1	1	0	1	Depression	3.	2	1	0

	Nationality	Fidelity	Modality	Date	Date_Cat	Prevention	Format	ModalityBH vNBH	AF	AG	AH	AI	AJ	AK	AL
118	0	0	3	2010	2	0	1	0							
119	0	0	3	2010	2	0	1	0							
120	1	0	8	2015	2	1	0	0							
121	0	1	1	2012	2	Blank	1	Blank							
122	1	1	2	2010	2	1	0	1							
123	1	1	2	2010	2	1	0	1							
124	1	1	2	2010	2	1	0	1							
125	1	1	2	2010	2	1	0	1							
126	0	1	4	2011	2	Blank	1	Blank							
127	0	1	4	2011	2	Blank	1	Blank							
128	1	1	8	2016	2	1	0	0							
129	1	1	4	2006	1	0	1	1							
130	1	0	2	2014	2	0	0	1							
131	1	0	2	2014	2	0	0	1							
132	1	0	7	2008	1	0	0	0							
133	1	0	7	2008	1	0	0	0							
134	1	1	4	2018	2	Blank	1	1							
135	1	1	4	2018	2	Blank	1	1							
136	1	1	5	2015	2	0	1	0							
137	1	1	5	2015	2	0	1	0							
138	1	1	1	2001	1	0	1	1							
139	1	1	1	2001	1	0	1	1							
140	1	0	7	2015	2	0	0	0							
141	1	0	7	2015	2	0	0	0							
142	0	1	3	2015	2	1	1	0							
143	0	1	3	2015	2	1	1	0							
144	1	1	2	2016	2	1	0	1							
145	1	1	2	2016	2	1	0	1							
146	1	0	2	2018	2	Blank	0	1							
147	1	0	2	2018	2	Blank	0	1							
148	1	0	2	2018	2	Blank	0	1							
149	1	0	2	2018	2	Blank	0	1							
150	0	1	3	1995	0	0	1	0							
151	0	1	3	1995	0	0	1	0							
152	0	1	3	1999	0	0	1	0							
153	0	1	3	1999	0	0	1	0							
154	0	1	2	2004	1	0	0	1							
155	0	1	2	2004	1	0	0	1							
156	0	1	2	2004	1	0	0	1							

	AM	AN	AO	AP	AQ	AR	AS
118							
119							
120							
121							
122							
123							
124							
125							
126							
127							
128							
129							
130							
131							
132							
133							
134							
135							
136							
137							
138							
139							
140							
141							
142							
143							
144							
145							
146							
147							
148							
149							
150							
151							
152							
153							
154							
155							
156							

	Study name	Comparison	Time point	Std diff in means	Standard error	Sample size	Effect direction	Std diff in means
157	Rohde 2004b	CWD-A v Life Skills	Baseline-End	0.382	0.212	91	Positive	0.382
158	Rohde 2014a	Combined Vs consecutive FFT + CWD	Baseline-12	0.742	0.314	44	Positive	0.742
159	Rohde 2014a	Combined Vs consecutive FFT + CWD	Baseline-End	0.762	0.315	44	Positive	0.762
160	Rohde 2014b	Combined Vs consecutive FFT + CWD	Baseline-12	-0.088	0.302	45	Negative	-0.088
161	Rohde 2014b	Combined Vs consecutive FFT + CWD	Baseline-End	0.912	0.317	45	Positive	0.912
162	Rohde 2015	CB Bibliotherapy v Brochure control	Baseline-12	0.067	0.155	190	Positive	0.067
163	Rohde 2015	CB Bibliotherapy v Brochure control	Baseline-End	0.278	0.155	190	Positive	0.278
164	Rohde 2015	CB Group Depression Intervention v	Baseline-12	-0.039	0.155	188	Negative	-0.039
165	Rohde 2015	CB Group Depression Intervention v	Baseline-End	0.398	0.156	188	Positive	0.398
166	Ruggiero 2015	BBN + ASH v Control	Baseline-12	0.037	0.110	337	Positive	0.037
167	Salerno_2016/Rhind_2014	ECHO v TAU	Baseline-12	-0.280	0.174	149	Negative	-0.280
168	Salloum 2012	GTI-CN v GTI-C	Baseline-12	0.568	0.255	64	Positive	0.568
169	Salloum 2012	GTI-CN v GTI-C	Baseline-End	0.040	0.251	64	Positive	0.040
170	Saltzer 2018	CBTv PDT	Baseline-12	-0.214	0.235	73	Negative	-0.214
171	Saltzer 2018	CBTv PDT	Baseline-End	0.007	0.235	73	Positive	0.007
172	Sandler 2019a	new beginnings program v low dosage attentional	Baseline-12	0.083	0.070	830	Positive	0.083
173	Sandler 2019a	new beginnings program v low dosage attentional	Baseline-End	0.256	0.070	830	Positive	0.256
174	Sandler 2019b	new beginnings program v low dosage attentional	Baseline-12	0.171	0.070	830	Positive	0.171
175	Sandler 2019b	new beginnings program v low dosage attentional	Baseline-End	0.315	0.070	830	Positive	0.315
176	Santacruz 2006	Bibliotherapy and games v no treatment	Baseline-12	1.585	0.391	39	Positive	1.585
177	Santacruz 2006	Bibliotherapy and games v no treatment	Baseline-End	1.119	0.369	39	Positive	1.119
178	Santacruz 2006	Emotive performance v no treatment	Baseline-12	3.065	0.486	40	Positive	3.065
179	Santacruz 2006	Emotive performance v no treatment	Baseline-End	3.133	0.492	40	Positive	3.133
180	Saulsberry 2013	Motivational Interview v Brief Advice	Baseline-12	-0.267	0.221	83	Negative	-0.267
181	Saulsberry 2013	Motivational Interview v Brief Advice	Baseline-End	-0.448	0.222	83	Negative	-0.448
182	Schaeffer 2014	Community Apprentiship-focused training vs Control	Baseline-12	0.069	0.203	97	Positive	0.069
183	Schaeffer 2014	Community Apprentiship-focused training vs Control	Baseline-End	0.021	0.203	97	Positive	0.021
184	Schneider 2013	Separation Anxiety CBT v Generalised	Baseline-12	0.000	0.309	42	Positive	0.000
185	Schneider 2013	Separation Anxiety CBT v Generalised	Baseline-End	0.084	0.309	42	Positive	0.084
186	Scott 2010	Incredible years + Spoke v Control	Baseline-12	-0.046	0.153	172	Negative	-0.046
187	Sheffield 2006	Indicated Intervention v Control	Baseline-12	-0.155	0.131	235	Negative	-0.155
188	Sheffield 2006	Indicated Intervention v Control	Baseline-End	0.087	0.131	235	Positive	0.087
189	Silk 2018	CBTv CCT	Baseline-12	0.512	0.247	133	Positive	0.512
190	Silverman 1999	Continguency v control	Baseline-12	0.590	0.399	41	Positive	0.590
191	Silverman 1999	Continguency v control	Baseline-End	0.374	0.396	41	Positive	0.374
192	Silverman 1999	Self control v control	Baseline-12	0.255	0.396	40	Positive	0.255
193	Silverman 1999	Self control v control	Baseline-End	0.686	0.403	40	Positive	0.686
194	Silverman 2009	CBT Y + P v CBT Y	Baseline-12	0.182	0.272	55	Positive	0.182
195	Silverman 2009	CBT Y + P v CBT Y	Baseline-End	-0.137	0.241	70	Negative	-0.137

	Std Err	Hedges's g	Std Err	ID	Setting	EOT	Agent	Severity	Contrast	Intensity	Disorder_code1	Disorder_Code2	Disorder_code3	Age	Manual
157	0.212	0.379	0.210	110	1	0	1	1	0	1	Depression	3.	2	1	0
158	0.314	0.729	0.309	111	0	0	0	1	0	1	Substance	2.	5	1	0
159	0.315	0.748	0.309	112	0	0	0	1	0	1	Substance	2.	5	1	0
160	0.302	-0.087	0.297	113	0	0	0	1	0	1	Depression	3.	2	1	0
161	0.317	0.897	0.311	114	0	0	0	1	0	1	Depression	3.	2	1	0
162	0.155	0.067	0.154	115	2	0	1	1	1	0	Depression	3.	2	1	0
163	0.155	0.277	0.155	116	2	0	1	1	1	0	Depression	3.	2	1	0
164	0.155	-0.039	0.155	117	2	0	1	1	1	0	Depression	3.	2	1	0
165	0.156	0.396	0.156	118	2	0	1	1	1	0	Depression	3.	2	1	0
166	0.110	0.037	0.110	229	0	1	0	1	1	0	PTSD	6. PTSD	4	1	1
167	0.174	-0.278	0.173	230	0	1	0	0	2	0	Eating	5.	3	1	0
168	0.255	0.562	0.252	119	2	0	0	1	0	1	PTSD	6. PTSD	4	0	0
169	0.251	0.039	0.248	120	2	0	0	1	0	1	PTSD	6. PTSD	4	0	0
170	0.235	-0.212	0.233		0	0	0	1	0	2	Anxiety	4.	0	1	0
171	0.235	0.006	0.232		0	0	0	1	0	2	Anxiety	4.	0	1	0
172	0.070	0.083	0.070		1	0	1	0	0	1	Conduct	1.	1	1	0
173	0.070	0.255	0.070		1	0	1	0	0	1	Conduct	1.	1	1	0
174	0.070	0.171	0.070		1	0	1	0	0	1	Depression	3.	2	1	0
175	0.070	0.315	0.070		1	0	1	0	0	1	Depression	3.	2	1	0
176	0.391	1.553	0.383	121	1	0	1	1	3	1	Anxiety	4.	0	0	1
177	0.369	1.097	0.362	122	1	0	1	1	3	1	Anxiety	4.	0	0	1
178	0.486	3.006	0.477	123	1	0	1	1	3	1	Anxiety	4.	0	0	1
179	0.492	3.072	0.482	124	1	0	1	1	3	1	Anxiety	4.	0	0	1
180	0.221	-0.265	0.219	125	1	0	0	1	0	0	Depression	3.	2	1	1
181	0.222	-0.444	0.220	126	1	0	0	1	0	0	Depression	3.	2	1	1
182	0.203	0.068	0.202	127	1	0	1	0	2	2	Substance	2.	5	1	0
183	0.203	0.021	0.202	129	1	0	1	0	2	2	Substance	2.	5	1	1
184	0.309	0.000	0.303	128	0	0	0	1	0	1	Anxiety	4.	0	0	0
185	0.309	0.082	0.303	130	0	0	0	1	0	1	Anxiety	4.	0	0	0
186	0.153	-0.046	0.152	231	2	1	1	1	2	1	Conduct	1.	1	0	0
187	0.131	-0.154	0.131	131	2	0	1	1	2	0	Depression	3.	2	1	0
188	0.131	0.087	0.130	133	2	0	1	1	2	0	Depression	3.	2	1	0
189	0.247	0.509	0.246		Blank	0	0	1	0	0	Anxiety	4.	0	0	0
190	0.399	0.579	0.392	132	0	0	0	1	1	0	Anxiety	4.	0	0	0
191	0.396	0.367	0.389	134	0	0	0	1	1	0	Anxiety	4.	0	0	0
192	0.396	0.250	0.389	135	0	0	0	1	1	0	Anxiety	4.	0	0	0
193	0.403	0.673	0.395	136	0	0	0	1	1	0	Anxiety	4.	0	0	0
194	0.272	0.179	0.269	137	0	0	1	1	0	1	Anxiety	4.	0	0	0
195	0.241	-0.136	0.238	138	0	0	1	1	0	1	Anxiety	4.	0	0	0

	Nationality	Fidelity	Modality	Date	Date_Cat	Prevention	Format	ModalityBHvNBH	AF	AG	AH	AI	AJ	AK	AL
157	0	1	2	2004	1	0	0	1							
158	0	1	7	2014	2	1	0	0							
159	0	1	7	2014	2	1	0	0							
160	0	1	7	2014	2	1	0	0							
161	0	1	7	2014	2	1	0	0							
162	0	1	1	2015	2	1	1	1							
163	0	1	1	2015	2	1	1	1							
164	0	1	2	2015	2	1	0	1							
165	0	1	2	2015	2	1	0	1							
166	0	1	1	2015	2	0	1	1							
167	1	0	4	2014	2	0	1	1							
168	0	0	2	2012	2	0	0	1							
169	0	0	2	2012	2	0	0	1							
170	1	1	1	2018	2	Blank	1	1							
171	1	1	1	2018	2	Blank	1	1							
172	0	1	4	2019	2	Blank	0	1							
173	0	1	4	2019	2	Blank	0	1							
174	0	1	4	2019	2	Blank	0	1							
175	0	1	4	2019	2	Blank	0	1							
176	1	0	1	2006	1	0	1	1							
177	1	0	1	2006	1	0	1	1							
178	1	0	1	2006	1	0	1	1							
179	1	0	1	2006	1	0	1	1							
180	0	0	8	2013	2	1	1	0							
181	0	0	8	2013	2	1	1	0							
182	0	0	8	2014	2	0	0	0							
183	0	0	8	2014	2	0	0	0							
184	1	1	7	2013	2	0	1	0							
185	1	1	7	2013	2	0	1	0							
186	1	1	4	2010	2	0	0	1							
187	1	1	2	2006	1	1	0	1							
188	1	1	2	2006	1	1	0	1							
189	0	1	1	2019	2	Blank	1	1							
190	0	1	1	1999	0	0	1	1							
191	0	1	1	1999	0	0	1	1							
192	0	1	1	1999	0	0	1	1							
193	0	1	1	1999	0	0	1	1							
194	0	1	1	2009	1	0	1	1							
195	0	1	1	2009	1	0	1	1							

	AM	AN	AO	AP	AQ	AR	AS
157							
158							
159							
160							
161							
162							
163							
164							
165							
166							
167							
168							
169							
170							
171							
172							
173							
174							
175							
176							
177							
178							
179							
180							
181							
182							
183							
184							
185							
186							
187							
188							
189							
190							
191							
192							
193							
194							
195							

	Study name	Comparison	Time point	Std diff in means	Standard error	Sample size	Effect direction	Std diff in means
196	Simon 2011	CFI v Control	Baseline-12	-0.092	0.230	86	Negative	-0.092
197	Simon 2011	CFI v PFI	Baseline-12	0.091	0.178	97	Positive	0.091
198	Slesnick 2009	EBT vs Services as usual	Baseline-12	1.142	0.293	58	Positive	1.142
199	Slesnick 2009	EBT vs Services as usual	Baseline-End	-0.135	0.274	58	Negative	-0.135
200	Slesnick 2009	FFT vs Services as usual	Baseline-12	1.141	0.289	61	Positive	1.141
201	Slesnick 2009	FFT vs Services as usual	Baseline-End	0.754	0.278	61	Positive	0.754
202	Slesnick 2013	CRA Vs Motivational Interviewing	Baseline-12	0.272	0.182	122	Positive	0.272
203	Slesnick 2013	CRA Vs Motivational Interviewing	Baseline-End	0.006	0.181	122	Positive	0.006
204	Solantaus 2010	Family Talk v Lets talk about the children	Baseline-12	0.169	0.195	106	Positive	0.169
205	Solantaus 2010	Family Talk v Lets talk about the children	Baseline-End	0.687	0.200	106	Positive	0.687
206	Somech 2012	Hitkashrut v Control	Baseline-12	0.720	0.164	182	Positive	0.720
207	Somech 2012	Hitkashrut v Control	Baseline-End	1.048	0.156	209	Positive	1.048
208	Sourander 2016	SFSW v Educational Control	Baseline-12	0.488	0.094	464	Positive	0.488
209	Spence 2000	Group CBT + Parent V Group CBT no Parent	Baseline-12	0.372	0.362	31	Positive	0.372
210	Spence 2000	Group CBT + Parent V Group CBT no Parent	Baseline-End	0.107	0.360	31	Positive	0.107
211	Spence 2006	Group CBT v Group CBT internet delivery	Baseline-12	0.730	0.320	42	Positive	0.730
212	Spence 2006	Group CBT v Group CBT internet delivery	Baseline-End	0.371	0.303	45	Positive	0.371
213	Spence 2011	Clinic v Online CBT	Baseline-12	0.266	0.214	88	Positive	0.266
214	Spence 2011	Clinic v Online CBT	Baseline-End	-0.232	0.214	88	Negative	-0.232
215	Spijkers 2013	PCTP v UC	Baseline-12	0.404	0.247	67	Positive	0.404
216	Spirito 2004	Motivational interviewing vs Standard Care	Baseline-12	0.247	0.180	124	Positive	0.247
217	Spirito 2004	Motivational interviewing vs Standard Care	Baseline-End	0.114	0.180	124	Positive	0.114
218	Spirito 2011	IMI + FCU Vs IMI	Baseline-End	-0.090	0.206	97	Negative	-0.090
219	Spirito 2011	IMI vs IMI + FCU	Baseline-12	0.501	0.225	83	Positive	0.501
220	Sportel 2013	CBM v Control	Baseline-12	0.316	0.202	121	Positive	0.316
221	Sportel 2013	CBM v Control	Baseline-End	0.173	0.201	121	Positive	0.173
222	Sportel 2013	CBT v Control	Baseline-12	0.203	0.202	119	Positive	0.203
223	Sportel 2013	CBT v Control	Baseline-End	-0.281	0.202	119	Negative	-0.281
224	Stefini 2017	CBT v Psychodynamic	Baseline-12	0.123	0.256	81	Positive	0.123
225	Stewart-Brown 2004	Webster-Stratton parenting program v Control	Baseline-12	0.053	0.186	116	Positive	0.053
226	Stice 2006	Dissonance v Assessmnet	Baseline-12	0.246	0.157	178	Positive	0.246
227	Stice 2006	Dissonance v Assessmnet	Baseline-End	0.873	0.163	178	Positive	0.873
228	Stice 2006	Healthy Weight v Assessment	Baseline-12	0.185	0.157	180	Positive	0.185
229	Stice 2006	Healthy Weight v Assessment	Baseline-End	0.452	0.158	180	Positive	0.452
230	Stice 2009	Dissonance v educational	Baseline-12	0.395	0.116	306	Positive	0.395
231	Stice 2009	Dissonance v educational	Baseline-End	0.438	0.116	306	Positive	0.438
232	Stice 2010	CB bibliotherapy v Brochure control	Baseline-12	0.073	0.217	109	Positive	0.073
233	Stice 2010	CB bibliotherapy v Brochure control	Baseline-End	0.098	0.220	108	Positive	0.098
234	Stice 2010	Group CBT v Brochure control	Baseline-12	0.241	0.217	117	Positive	0.241

	Std Err	Hedges's g	Std Err	ID	Setting	EOT	Agent	Severity	Contrast	Intensity	Disorder_code1	Disorder_Code2	Disorder_code3	Age	Manual
196	0.230	-0.091	0.228	232	2	1	0	1	3	0	Anxiety	4.	0	0	0
197	0.178	0.090	0.177	233	2	1	0	1	3	0	Anxiety	4.	0	0	0
198	0.293	1.127	0.289	139	1	0	0	1	1	1	Substance	2.	5	1	0
199	0.274	-0.133	0.270	141	1	0	0	1	1	1	Substance	2.	5	1	0
200	0.289	1.127	0.285	140	0	0	0	1	1	1	Substance	2.	5	1	0
201	0.278	0.745	0.275	142	0	0	0	1	1	1	Substance	2.	5	1	0
202	0.182	0.270	0.181	143	1	0	0	1	0	1	Substance	2.	5	1	0
203	0.181	0.006	0.180	144	1	0	0	1	0	1	Substance	2.	5	1	0
204	0.195	0.168	0.193	145	0	0	0	0	0	0	Depression	3.	2	1	0
205	0.200	0.682	0.198	146	0	0	0	0	0	0	Depression	3.	2	1	0
206	0.164	0.717	0.164	147	0	0	0	1	2	1	Conduct	1.	1	0	0
207	0.156	1.044	0.155	148	0	0	0	1	2	1	Conduct	1.	1	0	0
208	0.094	0.488	0.094	234	0	1	1	1	1	2	Conduct	1.	1	0	1
209	0.362	0.363	0.353	149	0	0	0	1	0	1	Anxiety	4.	0	0	0
210	0.360	0.105	0.351	150	0	0	0	1	0	1	Anxiety	4.	0	0	1
211	0.320	0.717	0.314	151	0	0	0	1	0	1	Anxiety	4.	0	0	0
212	0.303	0.365	0.297	152	0	0	0	1	0	1	Anxiety	4.	0	0	0
213	0.214	0.264	0.212	154	0	0	0	1	0	0	Anxiety	4.	0	1	0
214	0.214	-0.230	0.212	153	0	0	0	1	0	0	Anxiety	4.	0	1	0
215	0.247	0.399	0.244	235	0	1	1	1	2	0	Conduct	1.	1	0	0
216	0.180	0.245	0.179	155	0	0	1	0	2	0	Substance	2.	5	1	0
217	0.180	0.113	0.179	156	0	0	1	0	2	0	Substance	2.	5	1	0
218	0.206	-0.089	0.204	158	1	0	0	0	0	0	Substance	2.	5	1	0
219	0.225	0.496	0.223	157	1	0	0	0	0	0	Substance	2.	5	1	0
220	0.202	0.314	0.200	159	1	0	1	0	3	1	Anxiety	4.	0	1	0
221	0.201	0.172	0.200	160	1	0	1	0	3	1	Anxiety	4.	0	1	1
222	0.202	0.202	0.200	162	2	0	0	0	3	0	Anxiety	4.	0	1	0
223	0.202	-0.280	0.201	161	2	0	0	0	3	0	Anxiety	4.	0	1	1
224	0.256	0.122	0.254	229	0	1	0	1	0	2	Blank	Blank	3	1	0
225	0.186	0.053	0.185	236	1	1	1	1	3	0	Conduct	1.	1	0	0
226	0.157	0.245	0.157	171	1	0	1	0	3	0	Eating	5.	3	1	0
227	0.163	0.869	0.163	172	1	0	1	0	3	0	Eating	5.	3	1	0
228	0.157	0.184	0.156	173	1	0	1	0	3	0	Eating	5.	3	1	0
229	0.158	0.451	0.157	174	1	0	1	0	3	0	Eating	5.	3	1	0
230	0.116	0.394	0.116	169	1	0	1	0	1	0	Eating	5.	3	1	0
231	0.116	0.437	0.116	170	1	0	1	0	1	0	Eating	5.	3	1	0
232	0.217	0.072	0.215	163	2	0	0	0	1	0	Depression	3.	2	1	0
233	0.220	0.097	0.218	164	2	0	0	0	1	0	Depression	3.	2	1	0
234	0.217	0.240	0.216	165	2	0	0	0	1	0	Depression	3.	2	1	0

	Nationality	Fidelity	Modality	Date	Date_Cat	Prevention	Format	ModalityBH vNBH	AF	AG	AH	AI	AJ	AK	AL
196	1	1	2	2011	2	1	0	1							
197	1	1	4	2011	2	1	0	1							
198	0	1	3	2009	1	0	1	0							
199	0	1	3	2009	1	0	1	0							
200	0	1	3	2009	1	0	1	0							
201	0	1	3	2009	1	0	1	0							
202	0	1	1	2013	2	0	1	1							
203	0	1	1	2013	2	0	1	1							
204	1	0	3	2010	2	1	1	0							
205	1	0	3	2010	2	1	1	0							
206	1	1	4	2012	2	1	0	1							
207	1	1	4	2012	2	1	0	1							
208	1	1	4	2016	2	0	1	1							
209	1	0	7	2000	1	0	0	0							
210	1	0	7	2000	1	0	0	0							
211	1	1	2	2006	1	0	0	1							
212	1	1	2	2006	1	0	0	1							
213	1	1	1	2011	2	0	1	1							
214	1	1	1	2011	2	0	1	1							
215	1	0	4	2013	2	1	1	1							
216	0	1	8	2004	1	0	1	0							
217	0	1	8	2004	1	0	1	0							
218	0	1	8	2011	2	0	1	0							
219	0	1	8	2011	2	0	1	0							
220	1	1	1	2013	2	0	1	1							
221	1	1	1	2013	2	0	1	1							
222	1	1	2	2013	2	0	1	1							
223	1	1	2	2013	2	0	1	1							
224	1	1	1	2017	2	Blank	1	Blank							
225	1	0	4	2004	1	0	0	1							
226	0	1	2	2006	1	1	0	1							
227	0	1	2	2006	1	1	0	1							
228	0	1	2	2006	1	1	0	1							
229	0	1	2	2006	1	1	0	1							
230	0	1	1	2009	1	1	0	1							
231	0	1	2	2009	1	1	0	1							
232	0	0	1	2010	2	1	1	1							
233	0	0	1	2010	2	1	1	1							
234	0	1	2	2010	2	1	0	1							

	AM	AN	AO	AP	AQ	AR	AS
196							
197							
198							
199							
200							
201							
202							
203							
204							
205							
206							
207							
208							
209							
210							
211							
212							
213							
214							
215							
216							
217							
218							
219							
220							
221							
222							
223							
224							
225							
226							
227							
228							
229							
230							
231							
232							
233							
234							

	Study name	Comparison	Time point	Std diff in means	Standard error	Sample size	Effect direction	Std diff in means
235	Stice 2010	Group CBT v Brochure control	Baseline-End	0.921	0.225	117	Positive	0.921
236	Stice 2010	Group supportive expressive	Baseline-12	0.457	0.219	116	Positive	0.457
237	Stice 2010	Group supportive expressive	Baseline-End	0.363	0.218	116	Positive	0.363
238	Stolberg 1994a	Transfer, Skills and Support v control	Baseline-12	0.960	0.312	46	Positive	0.960
239	Stolberg 1994a	Transfer, Skills and Support v control	Baseline-End	0.338	0.287	50	Positive	0.338
240	Stolberg 1994b	Transfer, Skills and Support v control	Baseline-12	0.702	0.304	46	Positive	0.702
241	Stolberg 1994b	Transfer, Skills and Support v control	Baseline-End	0.205	0.286	50	Positive	0.205
242	Sussman 2012	Project Toward No Drug Abuse v Standard Care	Baseline-12	0.211	0.069	1182	Positive	0.211
243	Szapocznik 1989	PCT v Control	Baseline-12	-0.262	0.438	28	Negative	-0.262
244	Szapocznik 1989	PCT v Control	Baseline-End	-0.213	0.388	35	Negative	-0.213
245	Szapocznik 1989	SFT v Control	Baseline-12	0.681	0.441	30	Positive	0.681
246	Szapocznik 1989	SFT v Control	Baseline-End	0.579	0.393	35	Positive	0.579
247	Tanofsky-Kraff 2016	IPT v Healthy Eating	Baseline-12	2.610	0.290	88	Positive	2.610
248	Turner 2014	CBT v Telephone CBT	Baseline-12	0.224	0.236	72	Positive	0.224
249	Turner 2014	CBT v Telephone CBT	Baseline-End	-0.050	0.236	72	Negative	-0.050
250	Van Manen 2004	Social Cognitive Intervention v Social Skills Training	Baseline-12	-0.319	0.222	82	Negative	-0.319
251	Van Manen 2004	Social Cognitive Intervention v Social Skills Training	Baseline-End	-0.718	0.228	82	Negative	-0.718
252	Walker 2016	Motivational check-in vs Assessment only check in	Baseline-12	0.124	0.132	231	Positive	0.124
253	Walton 2013	Computerised Brief Intervention vs Control	Baseline-12	0.271	0.174	146	Positive	0.271
254	Walton 2013	Computerised Brief Intervention vs Control	Baseline-End	-0.102	0.172	148	Negative	-0.102
255	Walton 2013	Therapist Brief intervention vs Control	Baseline-12	0.258	0.175	137	Positive	0.258
256	Walton 2013	Therapist Brief intervention vs Control	Baseline-End	-0.276	0.175	137	Negative	-0.276
257	Waters 2009	Child + Parent CBT v Child CBT	Baseline-12	0.050	0.242	69	Positive	0.050
258	Waters 2009	Child + Parent CBT v Child CBT	Baseline-End	0.248	0.243	69	Positive	0.248
259	Webster- Stratton 1984	Individualised Therapy v Video group discussion	Baseline-12	0.610	0.368	31	Positive	0.610
260	Webster- Stratton 1984	Individualised Therapy v Video group discussion	Baseline-End	0.198	0.360	31	Positive	0.198
261	Webster-Stratton 1997	Parent training + Child training v Parent Training	Baseline-12	0.402	0.293	48	Positive	0.402
262	Webster-Stratton 1997	Parent training + Child training v Parent Training	Baseline-End	0.313	0.291	48	Positive	0.313
263	Webster-Stratton 2004	Parent + Child + Teacher Training v Parent Training	Baseline-12	0.281	0.319	40	Positive	0.281
264	Webster-Stratton 2004	Parent + Child + Teacher Training v Parent Training	Baseline-End	0.277	0.280	53	Positive	0.277
265	Weiss 1999a	Child Psychotherapy v academic	Baseline-12	0.581	0.162	160	Positive	0.581
266	Weiss 1999a	Child Psychotherapy v academic	Baseline-End	0.166	0.159	160	Positive	0.166
267	Weiss 1999b	Child Psychotherapy v academic	Baseline-12	0.243	0.159	160	Positive	0.243
268	Weiss 1999b	Child Psychotherapy v academic	Baseline-End	-0.019	0.158	160	Negative	-0.019
269	Weiss 2013	MST v TAU	Baseline-12	0.475	0.158	164	Positive	0.475
270	Weiss 2013	MST v TAU	Baseline-End	0.579	0.159	164	Positive	0.579
271	Wergeland 2014	Individual CBT v Group CBT	Baseline-12	-0.005	0.150	158	Negative	-0.005
272	Winters 2014	Adolescent + Parent Vs Adolescent Brief Intervention	Baseline-12	0.000	0.130	236	Positive	0.000
273	Wood 2009	Child + Parent CBT v Child CBT	Baseline-12	1.619	0.390	35	Positive	1.619

	Std Err	Hedges's g	Std Err	ID	Setting	EOT	Agent	Severity	Contrast	Intensity	Disorder_code1	Disorder_Code2	Disorder_code3	Age	Manual
235	0.225	0.915	0.223	166	2	0	0	0	1	0	Depression	3.	2	1	0
236	0.219	0.454	0.218	167	2	0	0	0	1	0	Depression	3.	2	1	0
237	0.218	0.360	0.217	168	2	0	0	0	1	0	Depression	3.	2	1	0
238	0.312	0.944	0.306	175	2	0	1	0	3	1	Anxiety	4.	0	0	0
239	0.287	0.333	0.282	177	2	0	1	0	3	1	Anxiety	4.	0	0	0
240	0.304	0.690	0.299	176	2	0	1	0	3	1	Depression	3.	2	0	0
241	0.286	0.202	0.281	178	2	0	1	0	3	1	Depression	3.	2	0	0
242	0.069	0.211	0.068	230	2	1	1	0	2	1	Blank	Blank	5	1	0
243	0.438	-0.254	0.426	179	0	0	0	1	1	1	Conduct	1.	1	0	0
244	0.388	-0.208	0.379	180	0	0	0	1	1	1	Conduct	1.	1	0	0
245	0.441	0.664	0.429	181	0	0	0	1	1	1	Conduct	1.	1	0	0
246	0.393	0.566	0.384	182	0	0	0	1	1	1	Conduct	1.	1	0	0
247	0.290	2.587	0.287	219	0	1	0	0	1	1	Blank	Blank	3	1	0
248	0.236	0.221	0.234	184	0	0	0	1	0	1	Anxiety	4.	0	1	0
249	0.236	-0.049	0.233	183	0	0	0	1	0	1	Anxiety	4.	0	1	0
250	0.222	-0.316	0.220	185	0	0	0	1	0	1	Conduct	1.	1	0	0
251	0.228	-0.711	0.226	186	0	0	0	1	0	1	Conduct	1.	1	0	0
252	0.132	0.124	0.131	214	2	1	0	1	1	2	Substance	2.	5	1	1
253	0.174	0.270	0.173	187	0	0	1	0	1	0	Substance	2.	5	1	0
254	0.172	-0.102	0.171	190	0	0	1	0	1	0	Substance	2.	5	1	0
255	0.175	0.256	0.174	188	0	0	0	0	1	0	Substance	2.	5	1	0
256	0.175	-0.274	0.174	191	0	0	0	0	1	0	Substance	2.	5	1	0
257	0.242	0.050	0.239	189	0	0	0	1	0	1	Anxiety	4.	0	0	1
258	0.243	0.245	0.240	192	0	0	0	1	0	1	Anxiety	4.	0	0	0
259	0.368	0.595	0.358	193	0	0	0	1	0	0	Conduct	1.	1	0	1
260	0.360	0.193	0.351	194	0	0	0	1	0	0	Conduct	1.	1	0	1
261	0.293	0.396	0.288	195	0	0	0	1	0	2	Conduct	1.	1	0	0
262	0.291	0.308	0.287	197	0	0	0	1	0	2	Conduct	1.	1	0	0
263	0.319	0.276	0.313	196	0	0	0	1	0	2	Conduct	1.	1	0	0
264	0.280	0.273	0.276	198	0	0	0	1	0	2	Conduct	1.	1	0	0
265	0.162	0.578	0.161	199	2	0	0	1	1	2	Depression	3.	2	0	1
266	0.159	0.166	0.158	200	2	0	0	1	1	2	Depression	3.	2	0	1
267	0.159	0.242	0.158	201	2	0	0	1	2	2	Conduct	1.	1	0	1
268	0.158	-0.019	0.158	202	2	0	0	1	2	2	Conduct	1.	1	0	1
269	0.158	0.473	0.158	203	2	0	0	1	2	0	Conduct	1.	1	1	0
270	0.159	0.576	0.159	205	2	0	0	1	2	0	Conduct	1.	1	1	0
271	0.150	-0.005	0.149	234	0	1	1	1	0	0	Blank	Blank	4	0	0
272	0.130	0.000	0.130	237	2	1	0	1	0	0	Substance	2.	5	1	0
273	0.390	1.583	0.381	204	0	0	0	1	0	1	Anxiety	4.	0	0	0

	Nationality	Fidelity	Modality	Date	Date_Cat	Prevention	Format	ModalityBH vNBH	AF	AG	AH	AI	AJ	AK	AL
235	0	1	2	2010	2	1	0	1							
236	0	1	6	2010	2	1	0	0							
237	0	1	6	2010	2	1	0	0							
238	0	0	5	1994	0	1	0	0							
239	0	0	5	1994	0	1	0	0							
240	0	0	5	1994	0	1	0	0							
241	0	0	5	1994	0	1	0	0							
242	0	1	5	2012	2	Blank	0	Blank							
243	0	0	6	1989	0	0	1	0							
244	0	0	6	1989	0	0	1	0							
245	0	0	3	1989	0	0	1	0							
246	0	0	3	1989	0	0	1	0							
247	0	1	6	2016	2	Blank	0	Blank							
248	1	1	1	2014	2	0	1	1							
249	1	1	1	2014	2	0	1	1							
250	1	0	1	2004	1	0	0	1							
251	1	0	1	2004	1	0	0	1							
252	0	1	8	2016	2	0	1	0							
253	0	0	8	2013	2	0	1	0							
254	0	0	8	2013	2	0	1	0							
255	0	0	8	2013	2	0	1	0							
256	1	0	8	2013	2	0	1	0							
257	1	1	7	2009	1	0	0	0							
258	1	1	7	2009	1	0	0	0							
259	0	0	4	1984	0	0	1	1							
260	0	0	4	1984	0	0	1	1							
261	0	1	4	1997	0	0	1	1							
262	0	1	4	1997	0	0	1	1							
263	0	1	7	2004	1	0	0	0							
264	0	1	7	2004	1	0	0	0							
265	0	0	6	1999	0	0	1	0							
266	0	0	6	1999	0	0	1	0							
267	0	0	6	1999	0	0	1	0							
268	0	0	6	1999	0	0	1	0							
269	0	1	4	2013	2	0	1	1							
270	0	1	4	2013	2	0	1	1							
271	1	1	1	2014	2	Blank	1	Blank							
272	0	1	7	2014	2	0	1	0							
273	0	1	3	2009	1	0	1	0							

	AM	AN	AO	AP	AQ	AR	AS
235							
236							
237							
238							
239							
240							
241							
242							
243							
244							
245							
246							
247							
248							
249							
250							
251							
252							
253							
254							
255							
256							
257							
258							
259							
260							
261							
262							
263							
264							
265							
266							
267							
268							
269							
270							
271							
272							
273							

	Study name	Comparison	Time point	Std diff in means	Standard error	Sample size	Effect direction	Std diff in means
274	Wood 2009	Child + Parent CBT v Child CBT	Baseline-End	1.141	0.365	35	Positive	1.141
275	Woods 2011	Kiwi ACE v Control	Baseline-12	2.469	0.542	24	Positive	2.469
276	Woods 2011	Kiwi ACE v Control	Baseline-End	1.116	0.439	24	Positive	1.116
277	Young 2009	Teen talk v School Counselling (TAU)	Baseline-12	0.279	0.331	41	Positive	0.279
278	Young 2009	Teen talk v School Counselling (TAU)	Baseline-End	1.385	0.363	41	Positive	1.385
279	Young 2012	Interpersonal Psychotherapy v TAU	Baseline-12	0.471	0.215	96	Positive	0.471
280	Young 2012	Interpersonal Psychotherapy v TAU	Baseline-End	1.277	0.234	94	Positive	1.277

	Std Err	Hedges's g	Std Err	ID	Setting	EOT	Agent	Severity	Contrast	Intensity	Disorder_code1	Disorder_Code2	Disorder_code3	Age	Manual
274	0.365	1.115	0.357	206	0	0	0	1	0	1	Anxiety	4.	0	0	0
275	0.542	2.388	0.524	207	2	0	1	1	1	0	Depression	3.	2	1	0
276	0.439	1.080	0.424	208	2	0	1	1	1	0	Depression	3.	2	1	0
277	0.331	0.274	0.325	209	2	0	1	0	2	0	Depression	3.	2	1	1
278	0.363	1.359	0.356	210	2	0	1	0	2	0	Depression	3.	2	1	1
279	0.215	0.467	0.213	211	2	0	1	1	2	0	Depression	3.	2	1	1
280	0.234	1.267	0.232	213	2	0	1	1	2	0	Depression	3.	2	1	1

	Nationality	Fidelity	Modality	Date	Date_Cat	Prevention	Format	ModalityBH vNBH	AF	AG	AH	AI	AJ	AK	AL
274	0	1	3	2009	1	0	1	0							
275	1	0	2	2011	2	0	0	1							
276	1	0	2	2011	2	0	0	1							
277	0	0	6	2009	1	1	0	0							
278	0	0	6	2009	1	1	0	0							
279	0	0	6	2012	2	1	0	0							
280	0	0	6	2012	2	1	0	0							

	AM	AN	AO	AP	AQ	AR	AS
274							
275							
276							
277							
278							
279							
280							
