# Supplementary material for: Long-term outcomes of psychological interventions on children and young people’s mental health: A systematic review and meta-analysis
Source: PLoS One. 2020 Nov 16;15(11):e0236525. doi: 10.1371/journal.pone.0236525 (PMC7668611; doi:10.1371/journal.pone.0236525)
Supplement: S4 File — (DOCX) [file pone.0236525.s005.docx]

**List of reports of studies included in the review**

1. Arnarson EO, Craighead WE. Prevention of depression among Icelandic adolescents. Behaviour Research and Therapy. 2009;47(7):577-85.
2. Arnarson EO, Craighead WE. Prevention of depression among Icelandic adolescents: a 12-month follow-up. Behaviour Research and Therapy. 2011;49(3):170-4.
3. Augimeri LK, Farrington DP, Koegl CJ, Day DM. The SNAP™ Under 12 Outreach Project: Effects of a Community Based Program for Children with Conduct Problems. Journal of Child and Family Studies. 2007;16(6):799-807.
4. August GJ, Lee SS, Bloomquist ML, Realmuto GM, Hektner JM. Maintenance effects of an evidence-based prevention innovation for aggressive children living in culturally diverse urban neighborhoods: The Early Risers Effectiveness Study. Journal of Emotional and Behavioral Disorders. 2004;12(4):194-205.
5. Barrett P, Farrell L, Dadds M, Boulter N. Cognitive-behavioral family treatment of childhood obsessive-compulsive disorder: long-term follow-up and predictors of outcome. Journal of the American Academy of Child and Adolescent Psychiatry. 2005;44(10):1005-14.
6. Barrett PM. Evaluation of cognitive-behavioral group treatments for childhood anxiety disorders. Journal of Clinical Child Psychology. 1998;27(4):459-68.
7. Barrett PM, Dadds MR, Rapee RM. Family treatment of childhood anxiety: a controlled trial. Journal of Consulting and Clinical Psychology. 1996;64(2):333-42.
8. Barrington J, Prior M, Richardson M, Allen K. Effectiveness of CBT versus standard treatment for childhood anxiety disorders in a community clinic setting. Behaviour Change. 2005;22(1):29-43.
9. Bayer JK, Beatson R, Bretherton L, Hiscock H, Wake M, Gilbertson T, Mihalopoulos C, Prendergast LA, Rapee RM. Translational delivery of Cool Little Kids to prevent child internalising problems: Randomised controlled trial. Australian & New Zealand Journal of Psychiatry. 2018 Feb;52(2):181-91.
10. Beardslee WR, Brent DA, Weersing VR, Clarke GN, Porta G, Hollon SD, et al. Prevention of depression in at-risk adolescents: longer-term effects. JAMA Psychiatry. 2013;70(11):1161-70.
11. Bernal ME, Klinnert MD, Schultz LA. Outcome evaluation of behavioral parent training and client-centered parent counseling for children with conduct problems. Journal of Applied Behavior Analysis. 1980;13(4):677-91.
12. Bernstein GA, Bernat DH, Victor AM, Layne AE. School-based interventions for anxious children: 3-, 6-, and 12-month follow-ups. Journal of the American Academy of Child and Adolescent Psychiatry. 2008;47(9):1039-47.
13. Bjørseth A, Wichstrom L. Effectiveness of parent-child interaction therapy (PCIT) in the treatment of young children's behavior problems. A randomized controlled study. PLoS One. 2016;11(9):e0159845.
14. Burke JD, Loeber R. The effectiveness of the Stop Now and Plan (SNAP) program for boys at risk for violence and delinquency. Prevention Science. 2015;16(2):242-53.
15. Butler S, Baruch G, Hickey N, Fonagy P. A randomized controlled trial of multisystemic therapy and a statutory therapeutic intervention for young offenders. Journal of the American Academy of Child and Adolescent Psychiatry. 2011;50(12):1220-35 e2.
16. Cartwright-Hatton S, McNally D, Field AP, Rust S, Laskey B, Dixon C, et al. A new parenting-based group intervention for young anxious children: results of a randomized controlled trial. Journal of the American Academy of Child and Adolescent Psychiatry. 2011;50(3):242-51 e6.
17. Cavell TA, Hughes JN. Secondary prevention as context for assessing change processes in aggressive children. Journal of School Psychology. 2000;38(3):199-235.
18. Clark HB, Prange ME, Lee B, Boyd LA, McDonald BA, Stewart ES. Improving adjustment outcomes for foster children with emotional and behavioral disorders: early findings from a controlled study on individualized services. Journal of Emotional and Behavioral Disorders. 1994;2(4):207-18.
19. Clark HK, Ringwalt CL, Hanley S, Shamblen SR, Flewelling RL, Hano MC. Project SUCCESS' effects on the substance use of alternative high school students. Addictive Behaviors. 2010;35(3):209-17.
20. Clarke G, DeBar LL, Pearson JA, Dickerson JF, Lynch FL, Gullion CM, et al. Cognitive behavioral therapy in primary care for youth declining antidepressants: a randomized trial. Pediatrics. 2016;137(5):1-13.
21. Clarke GN, Hawkins W, Murphy M, Sheeber LB, Lewinsohn PM, Seeley JR. Targeted prevention of unipolar depressive disorder in an at-risk sample of high school adolescents: a randomized trial of a group cognitive intervention. Journal of the American Academy of Child and Adolescent Psychiatry. 1995;34(3):312-21.
22. Clarke GN, Hornbrook M, Lynch F, Polen M, Gale J, Beardslee W, et al. A randomized trial of a group cognitive intervention for preventing depression in adolescent offspring of depressed parents. Archives of General Psychiatry. 2001;58(12):1127-34.
23. Clarke GN, Hornbrook M, Lynch F, Polen M, Gale J, O'Connor E, et al. Group cognitive-behavioral treatment for depressed adolescent offspring of depressed parents in a health maintenance organization. Journal of the American Academy of Child and Adolescent Psychiatry. 2002;41(3):305-13.
24. Cobham VE, Dadds MR, Spence SH. The role of parental anxiety in the treatment of childhood anxiety. Journal of Consulting and Clinical Psychology. 1998;66(6):893-905.
25. Cohen JA, Mannarino AP, Knudsen K. Treating sexually abused children: 1 year follow-up of a randomized controlled trial. Child Abuse & Neglect. 2005;29(2):135-45.
26. Conrod PJ, Castellanos-Ryan N, Mackie C. Long-term effects of a personality-targeted intervention to reduce alcohol use in adolescents. Journal of Consulting and Clinical Psychology. 2011;79(3):296-306.
27. Conrod PJ, Castellanos-Ryan N, Strang J. Brief, personality-targeted coping skills interventions and survival as a non-drug user over a 2-year period during adolescence. Archives of General Psychiatry. 2010;67(1):85-93.
28. Creswell C, Cruddace S, Gerry S, Gitau R, McIntosh E, Mollison J, et al. Treatment of childhood anxiety disorder in the context of maternal anxiety disorder: a randomised controlled trial and economic analysis. Health Technology Assessment. 2015;19(38):1-184, vii-viii.
29. Cunningham RM, Chermack ST, Zimmerman MA, Shope JT, Bingham CR, Blow FC, et al. Brief motivational interviewing intervention for peer violence and alcohol use in teens: one-year follow-up. Pediatrics. 2012;129(6):1083-90.
30. D'Amico EJ, Parast L, Shadel WG, Meredith LS, Seelam R, Stein BD. Brief motivational interviewing intervention to reduce alcohol and marijuana use for at-risk adolescents in primary care. Journal of consulting and clinical psychology. 2018 Sep;86(9):775.
31. Dakof GA, Henderson CE, Rowe CL, Boustani M, Greenbaum PE, Wang W, et al. A randomized clinical trial of family therapy in juvenile drug court. Journal of Family Psychology. 2015;29(2):232-41.
32. Deblinger E, Mannarino AP, Cohen JA, Runyon MK, Steer RA. Trauma-focused cognitive behavioral therapy for children: impact of the trauma narrative and treatment length. Depression and Anxiety. 2011;28(1):67-75.
33. Deblinger E, Mannarino AP, Cohen JA, Steer RA. A follow-up study of a multisite, randomized, controlled trial for children with sexual abuse-related PTSD symptoms. Journal of the American Academy of Child and Adolescent Psychiatry. 2006;45(12):1474-84.
34. Deblinger E, Steer RA, Lippmann J. Two-year follow-up study of cognitive behavioral therapy for sexually abused children suffering post-traumatic stress symptoms. Child Abuse & Neglect. 1999;23(12):1371-8.
35. Dishion TJ, Andrews DW. Preventing escalation in problem behaviors with high-risk young adolescents: immediate and 1-year outcomes. Journal of Consulting and Clinical Psychology. 1995;63(4):538-48.
36. Duong MT, Cruz RA, King KM, Violette HD, McCarty CA. Twelve-month outcomes of a randomized trial of the Positive Thoughts and Action program for depression among early adolescents. Prevention Science. 2016;17(3):295-305.
37. Estrada Y, Lee TK, Wagstaff R, Rojas LM, Tapia MI, Velázquez MR, Sardinas K, Pantin H, Sutton MY, Prado G. eHealth Familias Unidas: efficacy trial of an evidence-based intervention adapted for use on the internet with Hispanic families. Prevention Science. 2019 Jan 15;20(1):68-77.
38. Flannery-Schroeder E, Choudhury MS, Kendall PC. Group and individual cognitive-behavioral treatments for youth with anxiety disorders: 1-year follow-up. Cognitive Therapy and Research. 2005;29(2):253-9.
39. Foa EB, McLean CP, Capaldi S, Rosenfield D. Prolonged exposure vs supportive counseling for sexual abuse-related PTSD in adolescent girls: a randomized clinical trial. JAMA. 2013;310(24):2650-7.
40. Forgatch MS, DeGarmo DS. Parenting through change: an effective prevention program for single mothers. Journal of Consulting and Clinical Psychology. 1999;67(5):711-24.
41. Garcia-Lopez LJ, M. D-CM, Muela-Martinez JA, Espinosa-Fernandez L. Can parent training for parents with high levels of expressed emotion have a positive effect on their child's social anxiety improvement? Journal of Anxiety Disorders. 2014;28(8):812-22.
42. Ghaderi A, Kadesjö C, Björnsdotter A, Enebrink P. Randomized effectiveness trial of the family check-up versus internet-delivered parent training (iComet) for families of children with conduct problems. Scientific reports. 2018 Jul 31;8(1):1-5.
43. Godley MD, Godley SH, Dennis ML, Funk RR, Passetti LL, Petry NM. A randomized trial of assertive continuing care and contingency management for adolescents with substance use disorders. Journal of Consulting and Clinical Psychology. 2014;82(1):40-51.
44. Godley SH, Garner BR, Passetti LL, Funk RR, Dennis ML, Godley MD. Adolescent outpatient treatment and continuing care: main findings from a randomized clinical trial. Drug and Alcohol Dependence. 2010;110(1-2):44-54.
45. Goodyer IM, Reynolds S, Barrett B, Byford S, Dubicka B, Hill J, et al. Cognitive behavioural therapy and short-term psychoanalytical psychotherapy versus a brief psychosocial intervention in adolescents with unipolar major depressive disorder (IMPACT): a multicentre, pragmatic, observer-blind, randomised controlled superiority trial. Lancet Psychiatry. 2017;4(2):109-19.
46. Goossens FX, Lammers J, Onrust SA, Conrod PJ, de Castro BO, Monshouwer K. Effectiveness of a brief school-based intervention on depression, anxiety, hyperactivity, and delinquency: a cluster randomized controlled trial. European Child & Adolescent Psychiatry. 2016;25(6):639-48.
47. Gowers SG, Clark A, Roberts C, Griffiths A, Edwards V, Bryan C, et al. Clinical effectiveness of treatments for anorexia nervosa in adolescents: randomised controlled trial. British Journal of Psychiatry. 2007;191(5):427-35.
48. Hagen KA, Ogden T, Bjornebekk G. Treatment outcomes and mediators of parent management training: a one-year follow-up of children with conduct problems. Journal of Clinical Child & Adolescent Psychology. 2011;40(2):165-78.
49. Halldorsdottir T, Ollendick TH. Long-term outcomes of brief, intensive CBT for specific phobias: The negative impact of ADHD symptoms. Journal of Consulting and Clinical Psychology. 2016;84(5):465-71.
50. Hautmann C, Dose C, Duda-Kirchhof K, Greimel L, Hellmich M, Imort S, Katzmann J, Pinior J, Scholz K, Schürmann S, Metternich-Kaizman TW. Behavioral versus nonbehavioral guided self-help for parents of children with externalizing disorders in a randomized controlled trial. Behavior therapy. 2018 Nov 1;49(6):951-65.
51. Humayun S, Herlitz L, Chesnokov M, Doolan M, Landau S, Scott S. Randomized controlled trial of Functional Family Therapy for offending and antisocial behavior in UK youth. Journal of Child Psychology and Psychiatry. 2017;58(9):1023-32.
52. Hurlburt MS, Nguyen K, Reid J, Webster-Stratton C, Zhang J. Efficacy of the Incredible Years group parent program with families in Head Start who self-reported a history of child maltreatment. Child Abuse & Neglect. 2013;37(8):531-43.
53. Jouriles EN, McDonald R, Rosenfield D, Stephens N, Corbitt-Shindler D, Miller PC. Reducing conduct problems among children exposed to intimate partner violence: a randomized clinical trial examining effects of Project Support. Journal of Consulting and Clinical Psychology. 2009;77(4):705-17.
54. Kazdin AE, Siegel TC, Bass D. Cognitive problem-solving skills training and parent management training in the treatment of antisocial behavior in children. Journal of Consulting and Clinical Psychology. 1992;60(5):733-47.
55. Kendall PC, Hudson JL, Gosch E, Flannery-Schroeder E, Suveg C. Cognitive-behavioral therapy for anxiety disordered youth: a randomized clinical trial evaluating child and family modalities. Journal of Consulting and Clinical Psychology. 2008;76(2):282-97.
56. Lammers J, Goossens F, Conrod P, Engels R, Wiers RW, Kleinjan M. Effectiveness of a selective intervention program targeting personality risk factors for alcohol misuse among young adolescents: results of a cluster randomized controlled trial. Addiction. 2015;110(7):1101-9.
57. Larsson B, Fossum S, Clifford G, Drugli MB, Handegard BH, Morch WT. Treatment of oppositional defiant and conduct problems in young Norwegian children : results of a randomized controlled trial. European Child & Adolescent Psychiatry. 2009;18(1):42-52.
58. Le Grange D, Hughes EK, Court A, Yeo M, Crosby RD, Sawyer SM. Randomized clinical trial of parent-focused treatment and family-based treatment for adolescent anorexia nervosa. Journal of the American Academy of Child and Adolescent Psychiatry. 2016;55(8):683-92.
59. Le Grange D, Lock J, Agras WS, Bryson SW, Jo B. Randomized clinical trial of family-based treatment and cognitive-behavioral therapy for adolescent bulimia nervosa. Journal of the American Academy of Child and Adolescent Psychiatry. 2015;54(11):886-94 e2.
60. Lee SS, Victor AM, James MG, Roach LE, Bernstein GA. School-based interventions for anxious children: Long-term follow-up. Child Psychiatry & Human Development. 2016;47(2):183-93.
61. Letourneau EJ, Henggeler SW, McCart MR, Borduin CM, Schewe PA, Armstrong KS. Two-year follow-up of a randomized effectiveness trial evaluating MST for juveniles who sexually offend. Journal of Family Psychology. 2013;27(6):978-85.
62. Lewinsohn PM, Clarke GN, Hops H, Andrews J. Cognitive-behavioral treatment for depressed adolescents. Behavior Therapy. 1990;21(4):385-401.
63. Liddle HA, Dakof GA, Parker K, Diamond GS, Barrett K, Tejeda M. Multidimensional family therapy for adolescent drug abuse: results of a randomized clinical trial. American Journal of Drug and Alcohol Abuse. 2001;27(4):651-88.
64. Liddle HA, Dakof GA, Turner RM, Henderson CE, Greenbaum PE. Treating adolescent drug abuse: a randomized trial comparing multidimensional family therapy and cognitive behavior therapy. Addiction. 2008;103(10):1660-70.
65. Lochman JE, Baden RE, Boxmeyer CL, Powell NP, Qu L, Salekin KL, et al. Does a booster intervention augment the preventive effects of an abbreviated version of the coping power program for aggressive children? Journal of Abnormal Child Psychology. 2014;42(3):367-81.
66. Lochman JE, Dishion TJ, Powell NP, Boxmeyer CL, Qu L, Sallee M. Evidence-based preventive intervention for preadolescent aggressive children: One-year outcomes following randomization to group versus individual delivery. Journal of Consulting and Clinical Psychology. 2015;83(4):728-35.
67. Lochman JE, Wells KC. The Coping Power Program for preadolescent aggressive boys and their parents: outcome effects at the 1-year follow-up. Journal of Consulting and Clinical Psychology. 2004;72(4):571-8.
68. Lock J, Le Grange D, Agras WS, Moye A, Bryson SW, Jo B. Randomized clinical trial comparing family-based treatment with adolescent-focused individual therapy for adolescents with anorexia nervosa. Archives of General Psychiatry. 2010;67(10):1025-32.
69. Mahu IT, Doucet C, O'Leary-Barrett M, Conrod PJ. Can cannabis use be prevented by targeting personality risk in schools? Twenty-four-month outcome of the adventure trial on cannabis use: a cluster-randomized controlled trial. Addiction. 2015;110(10):1625-33.
70. Manassis K, Wilansky-Traynor P, Farzan N, Kleiman V, Parker K, Sanford M. The feelings club: randomized controlled evaluation of school-based CBT for anxious or depressive symptoms. Depression and Anxiety. 2010;27(10):945-52.
71. Mannarino AP, Cohen JA, Deblinger E, Runyon MK, Steer RA. Trauma-focused cognitive-behavioral therapy for children: sustained impact of treatment 6 and 12 months later. Child Maltreatment. 2012;17(3):231-41.
72. McGrath PJ, Lingley-Pottie P, Thurston C, MacLean C, Cunningham C, Waschbusch DA, et al. Telephone-based mental health interventions for child disruptive behavior or anxiety disorders: randomized trials and overall analysis. Journal of the American Academy of Child and Adolescent Psychiatry. 2011;50(11):1162-72.
73. Newton NC, Conrod PJ, Slade T, Carragher N, Champion KE, Barrett EL, et al. The long-term effectiveness of a selective, personality-targeted prevention program in reducing alcohol use and related harms: a cluster randomized controlled trial. Journal of Child Psychology and Psychiatry. 2016;57(9):1056-65.
74. Ogden T, Hagen KA. Multisystemic treatment of serious behaviour problems in youth: sustainability of effectiveness two years after intake. Child and Adolescent Mental Health. 2006;11(3):142-9.
75. Olivares J, Olivares-Olivares PJ, Rosa-Alcazar AI, Montesinos L, Macia D. The contribution of the therapist's competence in the treatment of adolescents with generalized social phobia. Psicothema. 2014;26(4):483-9.
76. Olivares-Olivares PJ, Rosa-Alcázar AI, Olivares-Rodríguez J. Does individual attention improve the effect of group treatment of adolescents with social phobia? International Journal of Clinical and Health Psychology. 2008;8(2):465-81.
77. Olthuis JV, McGrath PJ, Cunningham CE, Boyle MH, Lingley-Pottie P, Reid GJ, Bagnell A, Lipman EL, Turner K, Corkum P, Stewart SH. Distance-delivered parent training for childhood disruptive behavior (Strongest Families™): a randomized controlled trial and economic analysis. Journal of abnormal child psychology. 2018 Nov 1;46(8):1613-29.
78. O'Shea G, Spence SH, Donovan CL. Group versus individual interpersonal psychotherapy for depressed adolescents. Behavioural and Cognitive Psychotherapy. 2015;43(1):1-19.
79. Öst LG, Cederlund R, Reuterskiold L. Behavioral treatment of social phobia in youth: does parent education training improve the outcome? Behaviour Research and Therapy. 2015;67:19-29.
80. Öst LG, Svensson L, Hellstrom K, Lindwall R. One-session treatment of specific phobias in youths: a randomized clinical trial. Journal of Consulting and Clinical Psychology. 2001;69(5):814-24.
81. Pella JE, Drake KL, Tein JY, Ginsburg GS. Child anxiety prevention study: impact on functional outcomes. Child Psychiatry & Human Development. 2017;48(3):400-10.
82. Poppelaars M, Tak YR, Lichtwarck-Aschoff A, Engels RC, Lobel A, Merry SN, et al. A randomized controlled trial comparing two cognitive-behavioral programs for adolescent girls with subclinical depression: a school-based program (Op Volle Kracht) and a computerized program (SPARX). Behaviour Research and Therapy. 2016;80:33-42.
83. Rasing S, Creemers DH, Vermulst AA, Janssens JM, Engels RC, Scholte RH. Outcomes of a randomized controlled trial on the effectiveness of depression and anxiety prevention for adolescents with a high familial risk. International journal of environmental research and public health. 2018 Jul;15(7):1457.
84. Robin AL, Siegel PT, Moye A. Family versus individual therapy for anorexia: impact on family conflict. International Journal of Eating Disorders. 1995;17(4):313-22.
85. Robin AL, Siegel PT, Moye AW, Gilroy M, Dennis AB, Sikand A. A controlled comparison of family versus individual therapy for adolescents with anorexia nervosa. Journal of the American Academy of Child and Adolescent Psychiatry. 1999;38(12):1482-9.
86. Rohde P, Clarke GN, Mace DE, Jorgensen JS, Seeley JR. An efficacy/effectiveness study of cognitive-behavioral treatment for adolescents with comorbid major depression and conduct disorder. Journal of the American Academy of Child and Adolescent Psychiatry. 2004;43(6):660-8.
87. Rohde P, Stice E, Shaw H, Gau JM. Effectiveness trial of an indicated cognitive-behavioral group adolescent depression prevention program versus bibliotherapy and brochure control at 1- and 2-year follow-up. Journal of Consulting and Clinical Psychology. 2015;83(4):736-47.
88. Rohde P, Waldron HB, Turner CW, Brody J, Jorgensen J. Sequenced versus coordinated treatment for adolescents with comorbid depressive and substance use disorders. Journal of Consulting and Clinical Psychology. 2014;82(2):342-8.
89. Ruggiero KJ, Price M, Adams Z, Stauffacher K, McCauley J, Danielson CK, et al. Web intervention for adolescents affected by disaster: population-based randomized controlled trial. Journal of the American Academy of Child and Adolescent Psychiatry. 2015;54(9):709-17.
90. Salerno L, Rhind C, Hibbs R, Micali N, Schmidt U, Gowers S, et al. A longitudinal examination of dyadic distress patterns following a skills intervention for carers of adolescents with anorexia nervosa. European Child & Adolescent Psychiatry. 2016;25(12):1337-47.
91. Salloum A, Overstreet S. Grief and trauma intervention for children after disaster: exploring coping skills versus trauma narration. Behaviour Research and Therapy. 2012;50(3):169-79.
92. Salzer, S., et al. (2018). "Cognitive-Behavioral and Psychodynamic Therapy in Adolescents with Social Anxiety Disorder: A Multicenter Randomized Controlled Trial." Psychotherapy & Psychosomatics 87(4): 223-233.
93. Sandler, I., et al. (2019). "Randomized Effectiveness Trial of the New Beginnings Program for Divorced Families with Children and Adolescents." Journal of Clinical Child & Adolescent Psychology: 1-19.
94. Santacruz I, Méndez FJ, Sánchez-Meca J. Play therapy applied by parents for children with darkness phobia: comparison of two programmes. Child & Family Behavior Therapy. 2006;28(1):19-35.
95. Saulsberry A, Marko-Holguin M, Blomeke K, Hinkle C, Fogel J, Gladstone T, et al. Randomized clinical trial of a primary care internet-based intervention to prevent adolescent depression: one-year outcomes. Journal of the Canadian Academy of Child and Adolescent Psychiatry. 2013;22(2):106-17.
96. Schaeffer CM, Henggeler SW, Ford JD, Mann M, Chang R, Chapman JE. RCT of a promising vocational/employment program for high-risk juvenile offenders. Journal of Substance Abuse Treatment. 2014;46(2):134-43.
97. Schneider S, Blatter-Meunier J, Herren C, In-Albon T, Adornetto C, Meyer A, et al. The efficacy of a family-based cognitive-behavioral treatment for separation anxiety disorder in children aged 8-13: a randomized comparison with a general anxiety program. Journal of Consulting and Clinical Psychology. 2013;81(5):932-40.
98. Scott S, O'Connor TG, Futh A, Matias C, Price J, Doolan M. Impact of a parenting program in a high-risk, multi-ethnic community: the PALS trial. Journal of Child Psychology and Psychiatry. 2010;51(12):1331-41.
99. Sheffield JK, Spence SH, Rapee RM, Kowalenko N, Wignall A, Davis A, et al. Evaluation of universal, indicated, and combined cognitive-behavioral approaches to the prevention of depression among adolescents. Journal of Consulting and Clinical Psychology. 2006;74(1):66-79.
100. Silk, J. S., et al. (2018). "A randomized clinical trial comparing individual cognitive behavioral therapy and child-centered therapy for child anxiety disorders." Journal of Clinical Child and Adolescent Psychology 47(4): 542-554.
101. Silverman WK, Kurtines WM, Ginsburg GS, Weems CF, Rabian B, Serafini LT. Contingency management, self-control, and education support in the treatment of childhood phobic disorders: a randomized clinical trial. Journal of Consulting and Clinical Psychology. 1999;67(5):675-87.
102. Silverman WK, Kurtines WM, Jaccard J, Pina AA. Directionality of change in youth anxiety treatment involving parents: an initial examination. Journal of Consulting and Clinical Psychology. 2009;77(3):474-85.
103. Simon E, Bogels SM, Voncken JM. Efficacy of child-focused and parent-focused interventions in a child anxiety prevention study. Journal of Clinical Child & Adolescent Psychology. 2011;40(2):204-19.
104. Slesnick N, Erdem G, Bartle-Haring S, Brigham GS. Intervention with substance-abusing runaway adolescents and their families: results of a randomized clinical trial. Journal of Consulting and Clinical Psychology. 2013;81(4):600-14.
105. Slesnick N, Prestopnik JL. Comparison of family therapy outcome with alcohol-abusing, runaway adolescents. Journal of Marital and Family Therapy. 2009;35(3):255-77.
106. Solantaus T, Paavonen EJ, Toikka S, Punamaki RL. Preventive interventions in families with parental depression: children's psychosocial symptoms and prosocial behaviour. European Child & Adolescent Psychiatry. 2010;19(12):883-92.
107. Somech LY, Elizur Y. Promoting self-regulation and cooperation in pre-kindergarten children with conduct problems: a randomized controlled trial. Journal of the American Academy of Child and Adolescent Psychiatry. 2012;51(4):412-22.
108. Sourander A, McGrath PJ, Ristkari T, Cunningham C, Huttunen J, Lingley-Pottie P, et al. Internet-assisted parent training intervention for disruptive behavior in 4-year-old children: a randomized clinical trial. JAMA Psychiatry. 2016;73(4):378-87.
109. Spence SH, Donovan C, Brechman-Toussaint M. The treatment of childhood social phobia: the effectiveness of a social skills training-based, cognitive-behavioural intervention, with and without parental involvement. Journal of Child Psychology and Psychiatry. 2000;41(6):713-26.
110. Spence SH, Donovan CL, March S, Gamble A, Anderson RE, Prosser S, et al. A randomized controlled trial of online versus clinic-based CBT for adolescent anxiety. Journal of Consulting and Clinical Psychology. 2011;79(5):629-42.
111. Spence SH, Holmes JM, March S, Lipp OV. The feasibility and outcome of clinic plus internet delivery of cognitive-behavior therapy for childhood anxiety. Journal of Consulting and Clinical Psychology. 2006;74(3):614-21.
112. Spijkers W, Jansen DE, Reijneveld SA. Effectiveness of Primary Care Triple P on child psychosocial problems in preventive child healthcare: a randomized controlled trial. BMC Medicine. 2013;11(1):240.
113. Spirito A, Monti PM, Barnett NP, Colby SM, Sindelar H, Rohsenow DJ, et al. A randomized clinical trial of a brief motivational intervention for alcohol-positive adolescents treated in an emergency department. Journal of Pediatrics. 2004;145(3):396-402.
114. Spirito A, Sindelar-Manning H, Colby SM, Barnett NP, Lewander W, Rohsenow DJ, et al. Individual and family motivational interventions for alcohol-positive adolescents treated in an emergency department: results of a randomized clinical trial. Archives of Pediatrics & Adolescent Medicine. 2011;165(3):269-74.
115. Sportel BE, de Hullu E, de Jong PJ, Nauta MH. Cognitive bias modification versus CBT in reducing adolescent social anxiety: a randomized controlled trial. PLoS One. 2013;8(5):e64355.
116. Stefini A, Salzer S, Reich G, Horn H, Winkelmann K, Bents H, et al. Cognitive-behavioral and psychodynamic therapy in female adolescents with bulimia nervosa: a randomized controlled trial. Journal of the American Academy of Child and Adolescent Psychiatry. 2017;56(4):329-35.
117. Stewart-Brown S, Patterson J, Mockford C, Barlow J, Klimes I, Pyper C. Impact of a general practice based group parenting programme: quantitative and qualitative results from a controlled trial at 12 months. Archives of Disease in Childhood. 2004;89(6):519-25.
118. Stice E, Rohde P, Gau J, Shaw H. An effectiveness trial of a dissonance-based eating disorder prevention program for high-risk adolescent girls. Journal of Consulting and Clinical Psychology. 2009;77(5):825-34.
119. Stice E, Rohde P, Gau JM, Wade E. Efficacy trial of a brief cognitive-behavioral depression prevention program for high-risk adolescents: effects at 1- and 2-year follow-up. Journal of Consulting and Clinical Psychology. 2010;78(6):856-67.
120. Stice E, Shaw H, Burton E, Wade E. Dissonance and healthy weight eating disorder prevention programs: a randomized efficacy trial. Journal of Consulting and Clinical Psychology. 2006;74(2):263-75.
121. Stolberg AL, Mahler J. Enhancing treatment gains in a school-based intervention for children of divorce through skill training, parental involvement, and transfer procedures. Journal of Consulting and Clinical Psychology. 1994;62(1):147-56.
122. Sussman S, Sun P, Rohrbach LA, Spruijt-Metz D. One-year outcomes of a drug abuse prevention program for older teens and emerging adults: evaluating a motivational interviewing booster component. Health Psychology. 2012;31(4):476-85.
123. Szapocznik J, Rio A, Murray E, Cohen R, Scopetta M, Rivas-Vazquez A, et al. Structural family versus psychodynamic child therapy for problematic Hispanic boys. Journal of Consulting and Clinical Psychology. 1989;57(5):571-8.
124. Tanofsky-Kraff M, Crosby RD, Vannucci A, Kozlosky M, Shomaker LB, Brady SM, et al. Effect of adapted interpersonal psychotherapy versus health education on mood and eating in the laboratory among adolescent girls with loss of control eating. International Journal of Eating Disorders. 2016;49(5):490-8.
125. Turner CM, Mataix-Cols D, Lovell K, Krebs G, Lang K, Byford S, et al. Telephone cognitive-behavioral therapy for adolescents with obsessive-compulsive disorder: a randomized controlled non-inferiority trial. Journal of the American Academy of Child and Adolescent Psychiatry. 2014;53(12):1298-307 e2.
126. van Manen TG, Prins PJ, Emmelkamp PM. Reducing aggressive behavior in boys with a social cognitive group treatment: results of a randomized, controlled trial. Journal of the American Academy of Child and Adolescent Psychiatry. 2004;43(12):1478-87.
127. Walker DD, Stephens RS, Blevins CE, Banes KE, Matthews L, Roffman RA. Augmenting brief interventions for adolescent marijuana users: The impact of motivational check-ins. Journal of Consulting and Clinical Psychology. 2016;84(11):983-92.
128. Walton MA, Bohnert K, Resko S, Barry KL, Chermack ST, Zucker RA, et al. Computer and therapist based brief interventions among cannabis-using adolescents presenting to primary care: one year outcomes. Drug and Alcohol Dependence. 2013;132(3):646-53.
129. Waters AM, Ford LA, Wharton TA, Cobham VE. Cognitive-behavioural therapy for young children with anxiety disorders: Comparison of a Child + Parent condition versus a Parent Only condition. Behaviour Research and Therapy. 2009;47(8):654-62.
130. Webster-Stratton C. Randomized trial of two parent-training programs for families with conduct-disordered children. Journal of Consulting and Clinical Psychology. 1984;52(4):666-78.
131. Webster-Stratton C, Hammond M. Treating children with early-onset conduct problems: a comparison of child and parent training interventions. Journal of Consulting and Clinical Psychology. 1997;65(1):93-109.
132. Webster-Stratton C, Reid MJ, Hammond M. Treating children with early-onset conduct problems: intervention outcomes for parent, child, and teacher training. Journal of Clinical Child & Adolescent Psychology. 2004;33(1):105-24.
133. Weiss B, Catron T, Harris V, Phung TM. The effectiveness of traditional child psychotherapy. Journal of Consulting and Clinical Psychology. 1999;67(1):82-94.
134. Weiss B, Han S, Harris V, Catron T, Ngo VK, Caron A, et al. An independent randomized clinical trial of multisystemic therapy with non-court-referred adolescents with serious conduct problems. Journal of Consulting and Clinical Psychology. 2013;81(6):1027-39.
135. Wergeland GJ, Fjermestad KW, Marin CE, Haugland BS, Bjaastad JF, Oeding K, et al. An effectiveness study of individual vs. group cognitive behavioral therapy for anxiety disorders in youth. Behaviour Research and Therapy. 2014;57:1-12.
136. Winters KC, Lee S, Botzet A, Fahnhorst T, Nicholson A. One-year outcomes and mediators of a brief intervention for drug abusing adolescents. Psychology of Addictive Behaviors. 2014;28(2):464-74.
137. Wood JJ, McLeod BD, Piacentini JC, Sigman M. One-year follow-up of family versus child CBT for anxiety disorders: exploring the roles of child age and parental intrusiveness. Child Psychiatry & Human Development. 2009;40(2):301-16.
138. Woods B, Jose PE. Effectiveness of a school-based indicated early intervention program for Māori and Pacific adolescents. Journal of Pacific Rim Psychology. 2012;5(01):40-50.
139. Young JF, Gallop R, Mufson L. Mother-child conflict and its moderating effects on depression outcomes in a preventive intervention for adolescent depression. Journal of Clinical Child & Adolescent Psychology. 2009;38(5):696-704.
140. Young JF, Makover HB, Cohen JR, Mufson L, Gallop RJ, Benas JS. Interpersonal psychotherapy-adolescent skills training: anxiety outcomes and impact of comorbidity. Journal of Clinical Child & Adolescent Psychology. 2012;41(5):640-53.
