# Supplementary material for: Long-term outcomes of psychological interventions on children and young people’s mental health: A systematic review and meta-analysis
Source: PLoS One. 2020 Nov 16;15(11):e0236525. doi: 10.1371/journal.pone.0236525 (PMC7668611; doi:10.1371/journal.pone.0236525)
Supplement: S1 Table — (DOCX) [file pone.0236525.s006.docx]

**S3 Table: Observed frequencies for each study variable by disorder and associated chi-squared tests**

|  |  |  | **Anxiety** | **Conduct** | **Depressive** | **Eating** | **PTSD** | **Substance** | **Total** |
| --- | --- | --- | --- | --- | --- | --- | --- | --- | --- |
| Population |  |  |  |  |  |  |  |  |  |
|  | Age | < 12 | 34 | 31 | 3 | 0 | 7 | 0 | 75 |
|  |  | > 12 | 9 | 11 | 26 | 12 | 2 | 27 | 87 |
|  |  | Total | 43 | 42 | 29 | 12 | 9 | 27 | 162 |
|  |  |  |  |  |  |  | χ2(5) = | 90.63 | *p* < .001 |
|  |  |  |  |  |  |  |  |  |  |
|  |  |  |  |  |  |  |  |  |  |
|  | Nationality | US | 13 | 28 | 20 | 8 | 8 | 22 | 99 |
|  |  | Non-US | 30 | 16 | 10 | 4 | 1 | 5 | 66 |
|  |  | Total | 43 | 44 | 30 | 12 | 9 | 27 | 165 |
|  |  |  |  |  |  |  | χ2(5) = | 25.55 | *p* < .001 |
|  | Severity |  |  |  |  |  |  |  |  |
|  |  | Selected | 9 | 14 | 8 | 3 | 4 | 6 | 44 |
|  |  | Indicated | 34 | 30 | 22 | 9 | 5 | 21 | 121 |
|  |  | Total | 43 | 44 | 30 | 12 | 9 | 27 | 165 |
|  |  |  |  |  |  |  | χ2(5) = | 3.06 | *p* = .690 |
| Intervention |  |  |  |  |  |  |  |  |  |
|  | Modality | Indi. CBT | 14 | 3 | 4 | 2 | 6 | 2 | 31 |
|  |  | Group CBT | 8 | 2 | 14 | 2 | 1 | 0 | 27 |
|  |  | Family | 4 | 3 | 1 | 4 | 1 | 6 | 19 |
|  |  | Parent | 4 | 22 | 1 | 2 | 0 | 0 | 29 |
|  |  | Psychoeducation | 1 | 0 | 2 | 0 | 0 | 1 | 4 |
|  |  | Psychotherapy | 0 | 2 | 5 | 1 | 0 | 0 | 8 |
|  |  | Multiple | 12 | 8 | 2 | 1 | 1 | 4 | 28 |
|  |  | Other | 0 | 4 | 1 | 0 | 0 | 14 | 19 |
|  |  | Total | 43 | 44 | 30 | 12 | 9 | 27 | 165 |
|  |  |  |  |  |  |  | χ2(35) = | 164.53 | *p* < .001 |
|  |  |  |  |  |  |  |  |  |  |
|  | Format | Group/Mixed | 21 | 19 | 21 | 4 | 1 | 11 | 77 |
|  |  | Individual | 22 | 25 | 9 | 8 | 8 | 16 | 88 |
|  |  | Total | 43 | 44 | 30 | 12 | 9 | 27 | 165 |
|  |  |  |  |  |  |  | χ2 (5) = | 15.73 | *p =* .027 |
|  |  |  |  |  |  |  |  |  |  |
|  | Intensity | Low | 14 | 10 | 15 | 4 | 2 | 15 | 60 |
|  |  | Moderate | 26 | 18 | 12 | 3 | 7 | 8 | 74 |
|  |  | High | 3 | 16 | 3 | 5 | 0 | 4 | 31 |
|  |  | Total | 43 | 44 | 30 | 12 | 9 | 27 | 165 |
|  |  |  |  |  |  |  | χ2(10) = | 31.41 | *p* = .001 |
|  |  |  |  |  |  |  |  |  |  |
|  | Manualisation | Manualised | 15 | 30 | 20 | 8 | 8 | 20 | 101 |
|  |  | Non-manualised | 28 | 14 | 10 | 4 | 1 | 7 | 64 |
|  |  | Total | 43 | 44 | 30 | 12 | 9 | 27 | 165 |
|  |  |  |  |  |  |  | χ2 (5) = | 19.00 | *p* = .002 |
|  |  |  |  |  |  |  |  |  |  |
|  | Fidelity check | Absent | 13 | 23 | 10 | 4 | 3 | 11 | 64 |
|  |  | Present | 30 | 21 | 20 | 8 | 6 | 16 | 101 |
|  |  | Total | 43 | 44 | 30 | 12 | 9 | 27 | 165 |
|  |  |  |  |  |  |  | χ2 (5) = | 5.52 | *p* = .372 |
| Design and setting |  |  |  |  |  |  |  |  |  |
|  | Control type | Active | 23 | 13 | 8 | 6 | 6 | 8 | 64 |
|  |  | Attentional | 6 | 7 | 8 | 2 | 1 | 8 | 32 |
|  |  | TAU | 3 | 18 | 10 | 2 | 2 | 9 | 44 |
|  |  | Waitlist | 11 | 6 | 4 | 2 | 0 | 2 | 25 |
|  |  | Total | 43 | 44 | 30 | 12 | 9 | 27 | 165 |
|  |  |  |  |  |  |  | χ2 (15) = | 27.58 | *p* = 0.024 |
|  |  |  |  |  |  |  |  |  |  |
|  | Setting | Clinic | 23 | 18 | 9 | 9 | 8 | 11 | 78 |
|  |  | Community | 8 | 19 | 4 | 3 | 0 | 7 | 41 |
|  |  | School | 10 | 7 | 16 | 0 | 1 | 9 | 43 |
|  |  | Total | 41 | 44 | 29 | 12 | 9 | 27 | 162 |
|  |  |  |  |  |  |  | χ2 (10) = | 33.25 | *p* < .001 |
|  |  |  |  |  |  |  |  |  |  |
|  | Agent | Professional | 34 | 24 | 19 | 9 | 8 | 19 | 113 |
|  |  | Paraprofessional | 9 | 19 | 11 | 3 | 1 | 8 | 51 |
|  |  | Total | 43 | 43 | 30 | 12 | 9 | 27 | 164 |
|  |  |  |  |  |  |  | χ2 (5) = | 8.16 | *p* = .164 |
|  |  |  |  |  |  |  |  |  |  |
|  | Date | 1985-1999 | 5 | 10 | 4 | 2 | 2 | 0 | 23 |
|  |  | 2000-2009 | 16 | 12 | 5 | 4 | 2 | 5 | 44 |
|  |  | 2009-2018 | 22 | 22 | 21 | 6 | 5 | 22 | 98 |
|  |  | Total | 43 | 44 | 30 | 12 | 9 | 27 | 165 |
|  |  |  |  |  |  |  | χ2 (10) = | 14.87 | *p* = .137 |
