# Supplementary material for: Long-term outcomes of psychological interventions on children and young people’s mental health: A systematic review and meta-analysis
Source: PLoS One. 2020 Nov 16;15(11):e0236525. doi: 10.1371/journal.pone.0236525 (PMC7668611; doi:10.1371/journal.pone.0236525)
Supplement: S2 Table — (DOCX) [file pone.0236525.s007.docx]

**S4 table: Risk of bias for studies included in the meta-analysis**

| **Study** | **Random sequence generation (selection bias)** | **Allocation concealment (selection bias)** | **Blinding of participants and personnel (performance bias)** | **Blinding of outcome assessment (detection bias)** | **Incomplete outcome data (attrition bias)** | **Selective reporting (reporting bias)** | **Other bias** |
| --- | --- | --- | --- | --- | --- | --- | --- |
| Arnarson 2011/2009 | ? | ? | + | ? | - | ? | + |
| Augimeri 2007 | ? | + | - | - | ? | ? | + |
| August 2004 | ? | ? | - | ? | - | ? | + |
| Bai 2018 | + | + | - | - | + | + | + |
| Barrett 1996 | ? | ? | - | - | + | ? | + |
| Barrett 1998 | ? | ? | - | - | + | ? | + |
| Barrett 2005 | + | ? | - | + | + | ? | + |
| Barrington 2005 | ? | ? | - | - | + | - | + |
| Bayer 2018 | + | + | - | + | - | - | + |
| Beardslee 2013 | + | + | + | ? | + | ? | + |
| Bernal 1980 | ? | ? | - | - | + | ? | + |
| Bernstein 2008 | ? | + | - | - | + | + | + |
| Bjorseth 2016 | + | + | + | - | - | + | + |
| Burke 2015 | + | + | - | + | + | ? | + |
| Butler 2011 | + | ? | - | + | + | ? | + |
| Cartwright-Hatton-2011 | + | + | - | - | + | + | + |
| Cavell & Hughes 2000 | ? | ? | - | - | + | - | + |
| Clark 1994 | + | + | - | ? | + | ? | + |
| Clark 2010 | ? | ? | - | - | + | ? | + |
| Clarke 1995 | ? | ? | - | ? | + | ? | - |
| Clarke 2001 | + | + | - | - | - | ? | + |
| Clarke 2002 | ? | ? | - | + | + | ? | + |
| Clarke 2016 | ? | ? | - | + | + | ? | + |
| Cobham 1998 | + | ? | - | + | - | ? | - |
| Cohen 2005 | + | + | - | - | - | ? | - |
| Conrod 2010 | + | ? | - | - | - | ? | + |
| Conrod 2011/Conrod 2008 | + | ? | - | - | - | ? | + |
| Cottrell 2018 | + | ? | - | - | + | + | + |
| Creswell 2015 | + | + | ? | + | + | ? | + |
| Cunningham 2012 | + | + | - | - | + | + | + |
| Dakof 2015 | + | ? | - | + | - | ? | + |
| Damico 2018 | ? | ? | - | - | - | ? | + |
| Deblinger 1999 | ? | ? | - | ? | - | ? | + |
| Deblinger 2006 | ? | ? | - | + | - | ? | + |
| Dishion 1995 | + | + | - | - | + | ? | + |
| Duong 2016 | + | ? | - | + | + | ? | + |
| Estrada 2019 | ? | ? | - | - | + | ? | + |
| Flannery-Schroeder 2005 | + | ? | - | ? | - | ? | + |
| Foa 2013 | + | + | - | - | + | ? | + |
| Forgatch 1999 | ? | ? | - | - | + | ? | + |
| Garcia-Lopez 2014 | ? | ? | - | - | ? | ? | + |
| Ghaderi 2018 | ? | + | - | - | - | + | + |
| Godley 2010 | + | - | - | + | + | + | + |
| Godley 2014 | + | ? | - | - | + | ? | + |
| Goodyer 2017 | + | + | - | - | - | + | + |
| Goossens 2016 | ? | + | - | - | - | + | + |
| Gowers 2007 | + | + | - | + | - | + | + |
| Hagen 2011 | + | ? | - | - | - | ? | + |
| Hautmann 2018 | + | ? | - | - | ? | ? | + |
| Halldorsdottir 2016 | + | + | - | + | - | ? | + |
| Humayun 2017 | + | + | - | ? | - | ? | + |
| Hurlbert 2013 | + | + | - | - | - | ? | + |
| Jouriles 2009 | + | + | - | - | + | ? | - |
| Kazdin 1992 | ? | ? | - | - | - | ? | + |
| Kendall 2008 | + | + | + | - | - | ? | + |
| Lammers 2015 | + | + | - | - | - | + | + |
| Larssson 2009 | ? | ? | - | - | + | ? | + |
| Le Grange 2015 | ? | ? | - | + | - | ? | + |
| Le Grange 2016 | + | + | - | + | - | + | + |
| Lee 2016 | ? | ? | - | + | - | ? | + |
| Letourneau 2013 | + | ? | - | - | + | ? | + |
| Lewisohn 1990 | ? | ? | - | - | - | ? | + |
| Liddle 2001 | ? | ? | - | + | - | ? | - |
| Liddle 2008 | + | ? | - | - | - | ? | - |
| Lochman 2004 | ? | + | - | - | - | ? | + |
| Lochman 2014 | ? | ? | - | ? | - | ? | + |
| Lochman 2015 | ? | ? | - | - | + | ? | + |
| Lock 2010 | + | - | - | + | - | ? | + |
| Mahu 2015 | + | ? | ? | - | + | ? | + |
| Mannarino 2012/Deblinger 2011 | ? | ? | - | - | - | ? | + |
| Mannassis 2010 | + | + | + | - | + | - | + |
| McGrath 2011 | + | + | - | + | + | + | + |
| Newton 2016 | + | ? | ? | - | - | + | + |
| Ogden 2006 | ? | ? | - | - | + | - | + |
| Olivares 2014 | ? | ? | - | + | + | ? | + |
| Olivares-Olivares 2008 | + | ? | - | - | ? | ? | + |
| Olthuis 2018 | + | + | - | - | - | - | - |
| O'Shea 2015 | ? | ? | - | + | - | ? | + |
| Ost 2001 | ? | ? | ? | - | + | ? | + |
| Ost 2015 | + | + | - | + | + | ? | + |
| Pella 2017 | + | ? | - | ? | + | ? | + |
| Poppelaars 2016 | + | + | - | + | - | ? | + |
| Rasing 2018 | + | + | - | - | + | - | + |
| Robin 1995 | ? | ? | - | + | + | ? | + |
| Robin 1999 | ? | ? | - | + | + | ? | + |
| Rohde 2004 | + | ? | - | + | + | ? | + |
| Rohde 2014 | ? | ? | ? | - | - | ? | + |
| Rohde 2015 | + | ? | - | + | + | ? | + |
| Ruggiero 2015 | ? | ? | - | + | - | ? | + |
| Salerno 2016 | ? | + | - | - | - | + | + |
| Salloum 2012 | + | ? | - | - | - | + | + |
| Salzer 2018 | + | + | - | + | - | ? | + |
| Sandler 2019 | + | ? | - | - | ? | ? | + |
| Santacruz 2006 | ? | ? | - | - | + | ? | + |
| Saulsberry 2013 | + | + | - | - | + | ? | + |
| Schaeffer 2014 | + | + | - | - | + | ? | + |
| Schneider 2013 | + | + | ? | + | - | ? | + |
| Scott 2010 | + | + | - | + | + | ? | + |
| Sheffield 2006 | + | + | - | - | + | ? | + |
| Silk 2018 | ? | ? | - | - | - | - | + |
| Silverman 1999 | ? | ? | - | - | - | ? | + |
| Silverman 2009 | ? | + | - | - | - | - | + |
| Simon 2011 | ? | ? | - | ? | - | ? | + |
| Slesnick 2009 | + | + | - | - | - | ? | - |
| Slesnick 2013 | + | ? | - | ? | + | ? | - |
| Solantaus 2010 | + | ? | - | - | - | ? | + |
| Somech 2012 | + | + | - | + | - | ? | + |
| Sourander 2016 | + | + | - | - | - | + | + |
| Spence 2000 | ? | ? | - | - | - | ? | + |
| Spence 2006 | + | + | - | - | + | ? | + |
| Spence 2011 | + | + | - | + | + | ? | + |
| Spijkers 2013 | + | ? | - | - | - | + | + |
| Spirito 2004 | + | ? | - | - | + | ? | + |
| Spirito 2011 | + | + | - | - | - | ? | + |
| Sportel 2013 | + | ? | - | - | - | ? | + |
| Stefini 2017 | + | ? | - | + | + | ? | + |
| Stewart-Brown 2004 | ? | ? | - | - | - | - | + |
| Stice 2010 | + | + | - | + | - | ? | + |
| Stice 2009 | + | ? | - | + | ? | ? | + |
| Stice 2006 | ? | ? | - | - | + | + | + |
| Stolberg 1994 | ? | ? | - | - | + | ? | + |
| Sussman 2012 | + | ? | - | - | + | ? | + |
| Szapocznik 1989 | ? | - | ? | - | ? | ? | + |
| Tanofsky-Kraff 2016 | ? | ? | - | ? | - | + | + |
| Turner 2014 | + | + | - | + | - | - | - |
| Van Manen 2004 | ? | ? | + | - | ? | ? | + |
| Walker 2016 | ? | ? | - | - | + | ? | + |
| Walton 2013 | + | ? | - | - | + | + | + |
| Waters 2009 | ? | ? | - | + | - | ? | + |
| Webster-Stratton 1984 | ? | + | - | - | ? | ? | + |
| Webster-Stratton 1997 | ? | ? | - | - | + | ? | + |
| Webster Stratton 2004 | + | + | - | - | - | ? | - |
| Weiss 1999 | - | ? | - | ? | ? | ? | - |
| Weiss 2013 | + | + | - | - | + | ? | + |
| Wergeland 2014 | ? | ? | - | - | + | ? | + |
| Winters 2014 | + | + | - | + | + | ? | - |
| Wood 2009 | + | + | - | + | + | ? | + |
| Woods 2011 | + | ? | - | - | ? | ? | + |
| Young 2009 | + | ? | - | - | ? | ? | + |
| Young 2012 | + | + | - | + | + | ? | + |
