# Supplementary material for: Long-term outcomes of psychological interventions on children and young people’s mental health: A systematic review and meta-analysis
Source: PLoS One. 2020 Nov 16;15(11):e0236525. doi: 10.1371/journal.pone.0236525 (PMC7668611; doi:10.1371/journal.pone.0236525)
Supplement: S3 Table — a. Subgroup analysis at end of intervention for conduct and substance disorders. b. Subgroup analysis at end of intervention for depressive and anxiety disorders. (DOCX) [file pone.0236525.s008.docx]

**S5a table: Subgroup analysis at end of intervention for conduct and substance disorders**

|  |  | **Conduct Disorder** | | | | **Substance Misuse** | | | |
| --- | --- | --- | --- | --- | --- | --- | --- | --- | --- |
|  |  | **K** | **G (95% CI)** | **I^2^** | **Q(df)** | **K** | **G (95% CI)** | **I^2^** | **Q(df)** |
| **Population** |  |  |  |  |  |  |  |  |  |
| Age |  |  |  |  |  |  |  |  |  |
|  | Under 12 | 18 | 0.25 (0.08-0.42) | 72.54 |  |  |  |  |  |
|  | Over 12 | 3 | 0.15 (-0.36-0.65) | 77.48 | 0.93 (df=1) | 14 | 0.19 (0.01-0.38) | 78.47 | 0 (df=0) |
| Nationality |  |  |  |  |  |  |  |  |  |
|  | US | 16 | 0.19 (0.09-0.28) | 22.83 |  | 12 | 0.24 (0.04-0.45) | 80.11 |  |
|  | Non-US | 7 | 0.21 (-0.24-0.66) | 91.90 | 0.01 (df=1) | 2 | -0.09 (-0.47-0.64) | 54.00 | 2.29 (df=1) |
| Severity |  |  |  |  |  |  |  |  |  |
|  | Selected | 5 | 0.16 (0.02-0.30) | 40.38 |  | 7 | 0.02 (-0.06-0.10) | 0 |  |
|  | Indicated | 18 | 0.23 (0.02-0.44) | 80.40 | 0.29 (df=1) | 7 | 0.50 (0.12-0.87) | 80.78 | 5.95 (df=1)* |
| **Intervention** |  |  |  |  |  |  |  |  |  |
| Modality |  |  |  |  |  |  |  |  |  |
|  | Individual CBT / BT | 2 | -0.33 (-1.02-0.36) | 87.47 |  | 1 | 0.01 (-0.35-0.36) | 0 |  |
|  | Group CBT | 2 | 0.16 (-0.11-0.43) | 0 |  |  |  |  |  |
|  | Family-based | 2 | 0.11 (-0.69-0.91) | 66.74 |  | 6 | 0.45 (0.02-0.88) | 86.55 |  |
|  | Parenting training | 10 | 0.35 (0.11-0.59) | 84.75 |  |  |  |  |  |
|  | Psychoeducation/skills |  |  |  |  |  |  |  |  |
|  | Psychotherapy | 2 | -0.05 (-0.33-0.24) | 0 |  |  |  |  |  |
|  | Multiple intervention | 5 | 0.20 (0.03-0.36) | 0 |  | 2 | 0.34 (-0.32-0.99) | 78.97 |  |
|  | Other |  |  |  | 6.57 (df=5) | 5 | -0.07 (-0.23-0.09) | 0 | 5.94 (df=3) |
| Format |  |  |  |  |  |  |  |  |  |
|  | Group or mixed | 11 | 0.18 (-0.04-0.39) | 80.09 |  | 4 | 0.08 (-0.11-0.27) | 47.75 |  |
|  | Individual | 12 | 0.23 (0.01-0.45) | 74.05 | 0.10 (df=1) | 10 | 0.23 (-0.06-0.52) | 82.48 | 0.70 (df=1) |
| Intensity |  |  |  |  |  |  |  |  |  |
|  | Low | 5 | 0.16 (-0.20-0.52) | 84.55 |  | 5 | 0.00 (-0.11-0.12) | 15.36 |  |
|  | Moderate | 9 | 0.28 (-0.01-0.57) | 84.17 |  | 7 | 0.47 (0.07-0.86) | 85.38 |  |
|  | High | 9 | 0.10 (-0.21-0.21) | 0 | 1.36 (df=2) | 2 | 0.07 (-0.21-0.34) | 0 | 4.86 (df=2) |
| Manualisation |  |  |  |  |  |  |  |  |  |
|  | Manualised | 20 | 0.19 (0.03-0.35) | 78.08 |  | 12 | 0.24 (0.02-0.46) | 81.18 |  |
|  | Not manualised | 3 | 0.25 (-0.17-0.67) | 70.24 | 0.07 (df=1) | 2 | -0.03 (-0.25-0.18) | 0 | 2.92 (df=1) |
| Fidelity Check |  |  |  |  |  |  |  |  |  |
|  | Absent | 12 | 0.09 (-0.10-0.27) | 54.48 |  | 5 | 0.17 (-0.19-0.53) | 86.17 |  |
|  | Present | 11 | 0.30 (0.08-0.51) | 83.93 | 2.14 (df=1) | 9 | 0.22 (-0.02-0.45) | 71.44 | 0.04 (df=1) |
| **Design and setting** |  |  |  |  |  |  |  |  |  |
| Control type |  |  |  |  |  |  |  |  |  |
|  | Active | 12 | 0.05 (-0.15-0.25) | 73.45 |  | 6 | 0.46 (0.04-0.87) | 84.37 |  |
|  | Attentional | 2 | 0.18 (-0.58-0.94) | 51.46 |  | 4 | 0.02 (-0.37-0.41) | 70.67 |  |
|  | TAU | 8 | 0.41 (0.18-0.65) | 76.77 |  | 4 | 0.05 (-0.03-0.14) | 0 |  |
|  | Waitlist/no treatment | 1 | 0.12 (-0.11-0.35) | 0 | 5.70 (df=3) |  |  |  | 3.55 (df=2) |
| Setting |  |  |  |  |  |  |  |  |  |
|  | Clinic | 10 | 0.23 (-0.14-0.60) | 81.38 |  | 7 | 0.26 (-0.06-0.58) | 79.89 |  |
|  | Community | 8 | 0.17 (-0.04-0.37) | 78.23 |  | 6 | 0.17 (-0.20-0.54) | 80.92 |  |
|  | School | 5 | 0.20 (-0.04-0.44) | 62.95 | 0.10 (df=2) | 1 | 0.07 (-0.03-0.16) | 0 | 1.52 (df=2) |
| Agent |  |  |  |  |  |  |  |  |  |
|  | Professional | 14 | 0.22 (-0.05-0.50) | 83.82 |  | 10 | 0.29 (0.03-0.56) | 84.09 |  |
|  | Paraprofessional | 8 | 0.16 (0.07-0.25) | 8.22 | 5.89 (df=1) | 4 | -0.02 (-0.18-0.14) | 0 | 3.88 (df=1)* |
| Date |  |  |  |  |  |  |  |  |  |
|  | 1960-1999 | 7 | 0.12 (-0.11-0.35) | 18.17 |  |  |  |  |  |
|  | 2000-2009 | 6 | 0.02 (-0.25-0.29) | 63.02 |  | 5 | 0.56 (0.10-1.02) | 83.37 |  |
|  | 2010-2018 | 10 | 0.32 (0.10-0.54) | 86.26 | 3.08 (df=2) | 9 | 0.01 (-0.10-0.12) | 23.82 | 5.09 (df=1)* |

**S5b table: Subgroup analysis at end of intervention for depressive and anxiety disorders**

|  |  | **Depressive Disorders** | | | | **Anxiety Disorders** | | | |
| --- | --- | --- | --- | --- | --- | --- | --- | --- | --- |
|  |  | **K** | **G (95% CI)** | **I^2^** | **Q(df)** | **K** | **G (95% CI)** | **I^2^** | **Q(df)** |
| **Population** |  |  |  |  |  |  |  |  |  |
| Age |  |  |  |  |  |  |  |  |  |
|  | Under 12 | 3 | -0.07 (-0.56-0.43) | 80.12 |  | 27 | 0.74 (0.38-1.11) | 91.15 |  |
|  | Over 12 | 24 | 0.45 (0.29-0.61) | 74.54 | 4.42 (df=1) | 9 | 0.27 (-0.06-0.59) | 79.02 | 3.61 (df=1) |
| Nationality |  |  |  |  |  |  |  |  |  |
|  | US | 19 | 0.47 (0.30-0.64) | 74.60 |  | 11 | 1.07 (0.22-1.93) | 95.68 |  |
|  | Non-US | 9 | 0.21 (-0.06-0.48) | 80.13 | 2.52 (df=1) | 25 | 0.42 (0.21-0.64) | 77.38 | 2.11 (df=1) |
| Severity |  |  |  |  |  |  |  |  |  |
|  | Selected | 9 | 0.44 (0.13-0.74) | 84.36 |  | 4 | 0.01 (-0.22-0.25) | 26.79 |  |
|  | Indicated | 19 | 0.36 (0.19-0.53) | 74.20 | 0.17 (df=1) | 32 | 0.70 (0.39-1.01) | 90.43 | 11.72 (df=1)** |
| **Intervention** |  |  |  |  |  |  |  |  |  |
| Modality |  |  |  |  |  |  |  |  |  |
|  | Individual CBT / BT | 4 | 0.44 (0.20-0.69) | 59.27 |  | 13 | 0.78 (0.22-1.35) | 92.68 |  |
|  | Group CBT | 12 | 0.35 (0.09-0.61) | 82.45 |  | 7 | 0.01 (-0.19-0.21) | 33.57 |  |
|  | Family-based | 1 | 0.68 (0.29-1.07) | 0 |  | 4 | 1.74 (-0.06-3.55) | 97.42 |  |
|  | Parenting training | 1 | 0.32 (0.18-0.45) | 0 |  | 1 | 0.82 (0.32-1.31) | 0 |  |
|  | Psychoeducation/skills | 2 | 0.09 (-0.32-0.50) | 0 |  | 1 | 0.33 (-0.22-0.89) | 0 |  |
|  | Psychotherapy | 5 | 0.63 (0.20-1.06) | 83.04 |  |  |  |  |  |
|  | Multiple intervention | 2 | 0.58 (-0.05-1.20) | 51.01 |  | 10 | 0.48 (0.17-0.79) | 61.31 |  |
|  | Other | 1 | -0.44 (-0.88--0.01) | 0 | 20.15 (df=7)** |  |  |  | 18.98 (df=5)** |
| Format |  |  |  |  |  |  |  |  |  |
|  | Group or mixed | 19 | 0.44 (0.25-0.64) | 80.46 |  | 16 | 0.36 (0.14-0.58) | 65.63 |  |
|  | Individual | 9 | 0.29 (0.07-0.51) | 72.25 | 1.06 (df=1) | 20 | 0.84 (0.37-1.31) | 93.57 | 3.24 (df=1) |
| Intensity |  |  |  |  |  |  |  |  |  |
|  | Low | 15 | 0.43 (0.21-0.65) | 78.73 |  | 10 | 0.22 (-0.07-0.51) | 73.82 |  |
|  | Moderate | 10 | 0.34 (0.06-0.61) | 81.82 |  | 23 | 0.83 (0.41-1.26) | 92.31 |  |
|  | High | 3 | 0.36 (0.10-0.62) | 59.00 | 0.29 (df=2) | 3 | 0.46 (-0.25-1.17) | 79.32 | 5.52 (df=2) |
| Manualisation |  |  |  |  |  |  |  |  |  |
|  | Manualised | 23 | 0.38 (0.24-0.52) | 72.51 |  | 30 | 0.62 (0.31-0.93) | 90.03 |  |
|  | Not manualized | 5 | 0.45 (-0.14-1.03) | 90.07 | 0.05 (df=1) | 6 | 0.60 (-0.03-1.23) | 89.55 | 0.00 (df=1) |
| Fidelity |  |  |  |  |  |  |  |  |  |
|  | Low | 9 | 0.45 (0.08-0.82) | 83.43 |  | 12 | 0.56 (0.17-0.94) | 82.70 |  |
|  | High | 19 | 0.37 (0.21-0.52) | 75.22 | 0.17 (df=1) | 24 | 0.65 (0.28-1.01) | 91.64 | 0.11 (df=1) |
| **Design and setting** |  |  |  |  |  |  |  |  |  |
| Control type |  |  |  |  |  |  |  |  |  |
|  | Active | 8 | 0.34 (0.09-0.59) | 69.59 |  | 20 | 0.39 (0.18-0.60) | 65.47 |  |
|  | Attentional | 8 | 0.45 (0.24-0.67) | 59.31 |  | 6 | 1.82 (0.33-3.31) | 97.74 |  |
|  | TAU | 8 | 0.54 (0.17-0.91) | 89.64 |  | 1 | 0.02 (-0.54-0.58) | 0 |  |
|  | Waitlist/no treatment | 4 | 0.02 (-0.18-0.22) | 0 | 11.05 (df=3)* | 9 | 0.46 (0.02-0.90) | 86.07 | 5.29 (df=3) |
| Setting |  |  |  |  |  |  |  |  |  |
|  | Clinic | 8 | 0.52 (0.30-0.73) | 62.02 |  | 23 | 0.69 (0.29-1.09) | 91.39 |  |
|  | Community | 4 | 0.09 (-0.24-0.42) | 76.51 |  | 4 | 1.05 (0.05-2.06) | 91.49 |  |
|  | School | 15 | 0.44 (0.20-0.68) | 81.65 | 8.11 (df=2)* | 8 | 0.31 (-0.09-0.72) | 82.25 | 7.20 (df=2) |
| Agent |  |  |  |  |  |  |  |  |  |
|  | Professional | 17 | 0.31 (0.10-0.52) | 81.47 |  | 29 | 0.59 (0.29-0.89) | 90.17 |  |
|  | Paraprofessional | 11 | 0.49 (0.29-0.69) | 70.30 | 1.47 (df=1) | 8 | 0.68 (0.11-1.26) | 87.15 | 0.08 (df=1) |
| Date |  |  |  |  |  |  |  |  |  |
|  | 1984-1999 | 4 | 0.37 (0.02-0.72) | 59.44 |  | 5 | 0.44 (0.15-0.72) | 0 |  |
|  | 2000-2009 | 5 | 0.45 (0.02-0.89) | 82.13 |  | 16 | 1.06 (0.42-1.71) | 94.30 |  |
|  | 2010-2018 | 17 | 0.4 (0.18-0.61) | 81.66 | 0.08 (df=2) | 15 | 0.25 (0.02-0.48) | 74.60 | 5.78 (df=2) |
|  |  |  |  |  |  |  |  |  |  |
