# Supplementary material for: Long-term outcomes of psychological interventions on children and young people’s mental health: A systematic review and meta-analysis
Source: PLoS One. 2020 Nov 16;15(11):e0236525. doi: 10.1371/journal.pone.0236525 (PMC7668611; doi:10.1371/journal.pone.0236525)

**S6 Fig: Random effects funnel plots for each diagnostic group**

Random effects funnel plot for anxiety disorders (K=43)

Random effects funnel plot for conduct disorder (K=44)

Random effects funnel plot for depressive disorders (K=30)

Random effects funnel plot for substance misuse (K=27)


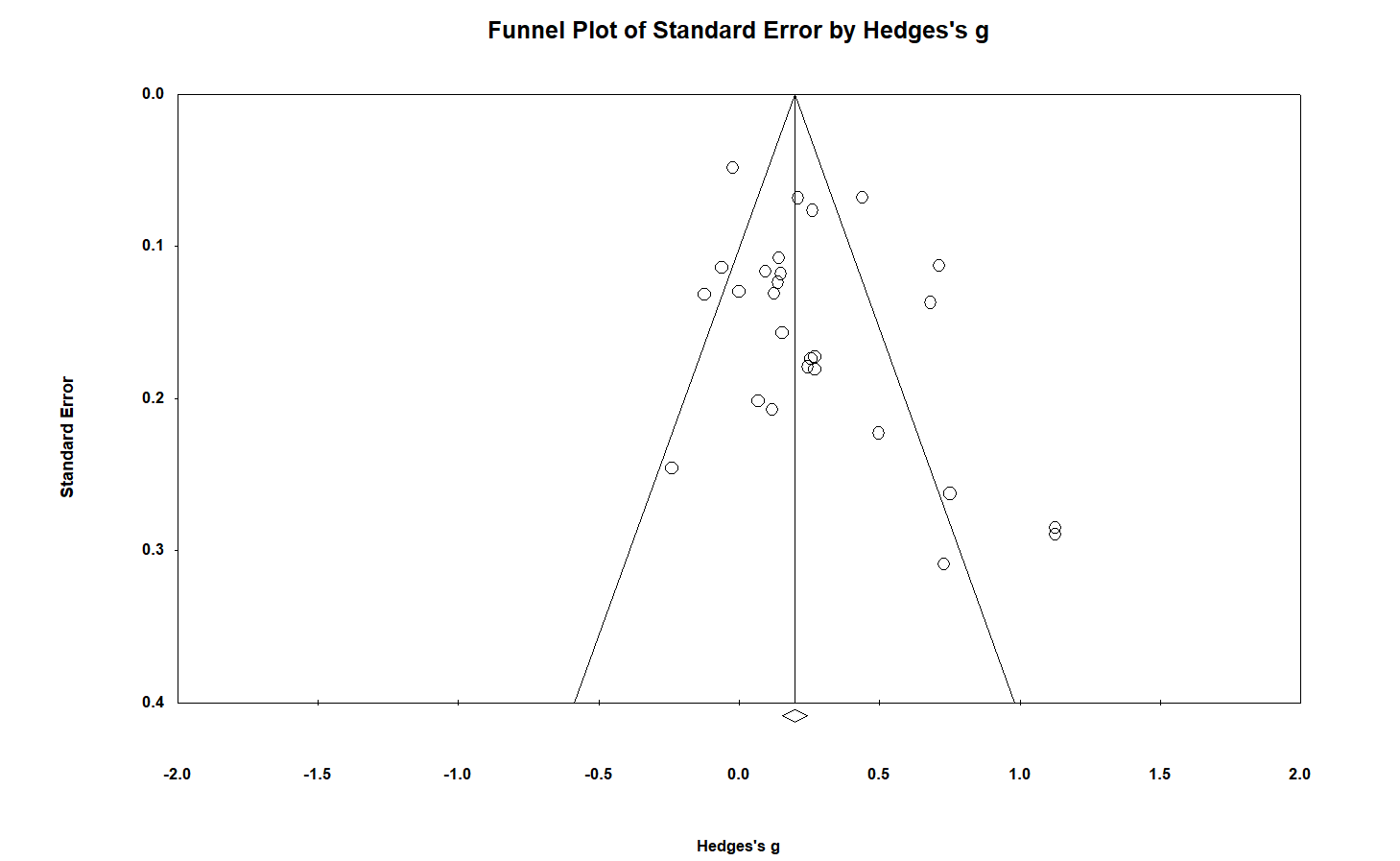

Supplement: S1 Fig — (DOCX) [file pone.0236525.s009.docx]
